# Formation of PVDF membranes with distinct pore morphologies interpreted through the framework of viscoelastic phase separation

**DOI:** 10.1038/s41598-026-50635-7

**Published:** 2026-05-09

**Authors:** Sven Johann Bohr, Bruno Richard Domnick, Clemens Alexowsky, Stéphan Barbe, Mathias Ulbricht

**Affiliations:** 1 Faculty of Applied Natural Sciences, Cologne University of Applied Sciences, Leverkusen, 51379 Germany; 2 Department of Technical Chemistry II, University of Duisburg-Essen, Essen, 45141 Germany; 3 Faculty of Process Engineering, Energy and Mechanical Systems, Cologne University of Applied Sciences, Cologne, 50679 Germany; 4 Evonik Operations GmbH, Essen, 45127 Germany

**Keywords:** Viscoelastic phase separation, Rheology, Membrane formation, VIPS, PVDF, Green solvent, Chemistry, Materials science

## Abstract

The formation of polyvinylidene fluoride (PVDF) membranes by vapor-induced phase separation (VIPS) is known to depend strongly on the dissolution temperature of the casting solution, yet the mechanism linking dissolution conditions to membrane morphology has remained unclear. Here we investigate this relationship by correlating dissolution temperature, solution rheology, polymer crystallization behavior, and membrane microstructure within the theoretical framework of viscoelastic phase separation (VPS). Rheological measurements, FTIR-ATR spectroscopy of solutions and membranes, and scanning electron microscopy of the resulting membranes reveal that PVDF/DMSO solutions exhibit a distinct temperature window bounded by the minimum and critical dissolution temperatures $$T_{d,min}$$ and $$T_{d,crit}$$. Below $$T_{d,crit}$$, incomplete dissolution leaves residual $$\alpha$$-polymorph microcrystallites in solution that act as thermoreversible multichain junctions. These junctions form a transient stress-bearing network that increases solution viscoelasticity and promotes elastic phase separation (EPS). Above $$T_{d,crit}$$ these junctions dissolve, reducing the viscoelastic constraint and allowing phase separation to proceed predominantly in the fluid phase separation (FPS) regime. Within the VPS framework, the Weissenberg number (*$Wi*$), defined as the ratio between the stress-relaxation time of the polymer-rich phase and the deformation time scale generated during phase separation, provides an experimentally accessible descriptor of this transition. A morphology regime diagram relating the initial Weissenberg number ($$Wi_i$$) to the observed membrane structures shows that bicontinuous morphologies occur for $$Wi_i \ge 1$$, whereas nodular structures form when $$Wi_i < 1$$. Remarkably, the condition $$Wi \approx 1$$ lies within the dissolution-temperature window previously associated with $$\alpha$$-polymorph prevalence, while higher dissolution temperatures favor $$\beta$$-phase formation due to increased chain mobility during demixing. These findings establish a mechanistic connection between dissolution temperature, transient microcrystalline junction networks, solution viscoelasticity, and membrane microstructure. More broadly, they demonstrate how viscoelastic phase-separation theory can provide a unified framework for understanding and rationally controlling membrane formation from semicrystalline polymer solutions.

## Introduction

Polyvinylidene fluoride (PVDF) is a semicrystalline fluoropolymer that occurs in different crystalline polymorphs. Of particular significance are the nonpolar $\alpha$-phase and the polar $\beta$-phase^1,2^. PVDF is frequently utilized in the fabrication of porous membranes^3,4^. The pore size and cross-section morphology of the membrane are critical factors that determine its suitability for specific applications^5^.

The fabrication of porous polymer membranes is typically accomplished by casting a film of a polymer solution that subsequently undergoes liquid nonsolvent-induced phase separation (NIPS) that will yield highly anisotropic pore morphologies, with a thin dense skin layer on top and a porous substructure^6,7^. Alternatively, nonsolvent vapor-induced phase separation (VIPS) may also be employed^8,9^. VIPS demonstrates distinct characteristics, namely a slower nonsolvent uptake rate and negligible solvent out-diffusion. These phenomena result in a substantial prolongation of phase separation processes, thereby facilitating the fabrication of skinless, isomorphic, porous membranes^10–13^.

PVDF dissolves in polar aprotic solvents, including dimethylformamide (DMF), dimethylacetamide (DMAc), and N-methyl-2-pyrrolidone (NMP)^14,15^. Emerging green solvents include dimethyl sulfoxide (DMSO) and triethyl phosphate (TEP)^16–21^. PVDF solutions exposed to VIPS conditions undergo solid-liquid demixing (polymer crystallization) and gelation prior to and/or concurrent with liquid-liquid phase separation (LLPS), due to the slow phase separation kinetics^22–25^.

Membrane morphology is tailored by modifying experimental parameters, such as PVDF concentration, dissolution temperature ($T_d$), VIPS exposure time, and relative humidity^26^. VIPS facilitates the formation of isotropic cross-sections devoid of macrovoids^27^. The microstructure can exhibit a range of characteristics, including lacy bicontinuous, sponge-like, and nodular^28,29^. In general, lacy bicontinuous structures are regarded as being superior to nodular structures due to the fact that nodular structures are lacking mechanical stability of the membrane^30,31^.

The correlation between $T_d$ and the microstructure of PVDF membranes prepared by VIPS is well documented^32–36^. Li et al.^37^ identified a critical dissolution temperature ($T_{d,crit}$) of PVDF, above which the membrane morphology undergoes a sudden transition from lacy bicontinuous to nodular. This transition was found to coincide with pronounced changes in both solution viscoelasticity and the crystalline polymorph ratio of the membrane. Membranes prepared from solutions dissolved below $T_{d,crit}$ predominantly contain the \mbox{$\alpha$-polymorph} and exhibit a lacy microstructure, whereas those prepared above $T_{d,crit}$ are enriched in the \mbox{$\beta$-polymorph} and display a nodular morphology. Importantly, the polymorph ratio is not merely a descriptor of the final solid state, but also reflects the crystallization pathway active during phase separation. In this sense, the fraction of $\alpha$-polymorph, $F_\alpha$, can be interpreted as an indicator of whether membrane formation is dominated by regrowth from surviving crystalline precursors or by conformational selection during demixing. Li et al. further proposed, as an extension of the self-seeding concept, that lower dissolution temperatures leave behind a higher density of crystalline nuclei or chain-fold aggregates in solution, which subsequently alter gelation and membrane formation. However, that study inferred the existence of such precursors from membrane morphology, polymorph selection, and water-triggered gelation behavior, and stated that further investigation was required to verify the proposed mechanism. In the present work, we address precisely this unresolved point by directly tracking $\alpha$-polymorph crystallites in PVDF solution, and their regrowth over time, in the absence of added water.

Annamalai et al.^38^ employed near-infrared spectroscopy to monitor water uptake during VIPS of PVDF solutions prepared at varying $T_d$. They showed that gelation occurs prior to LLPS and that this initial physical reorganization, induced by water absorption, is strongly influenced by $T_d$. For $T_d < T_{d,crit}$, early gelation gives rise to a viscoelastic network that restricts both water uptake and polymer chain contraction, thereby preserving bicontinuous morphologies. For $T_d > T_{d,crit}$, accelerated water uptake and increased polymer chain mobility delay gelation, enabling polymer chain contraction and favoring nodular structures. This distinction is also important for polymorph selection: early gelation mechanically constrains chain rearrangement and therefore limits the ability of solvent-nonsolvent interactions to alter chain conformation during phase separation, whereas delayed gelation leaves the chains mobile for longer and amplifies the influence of solvent and nonsolvent polarity on crystallization. Thus, the study established a direct link between viscoelasticity, mass-transfer kinetics, membrane pore morphology, and the molecular freedom required for polymorphic transition.

A mounting body of research with different polymers underscores the impact of viscoelasticity on membrane morphology. Tsai et al.^28^ demonstrated that the lacy structure formed in the initial stage of VIPS can be preserved by selecting solvents such as 2-pyrrolidinone, thereby increasing the viscoelasticity of the polymer-rich phase. This, in turn, promotes rapid gelation and prevents coarsening. Hung et al.^39^ showed that polymer chain entanglement suppresses macrovoid formation by altering the relaxation-demixing timescale ratio. Macrovoids are initiated only when polymer chain relaxation is faster than phase separation. Su et al.^40^ introduced the concept of a critical residence time in the metastable region, showing that membrane morphology depends on whether the residence time is shorter or longer than a threshold set by the degree of polymer chain entanglement. Feng et al.^41^ established that sulfonation of polyphenylenesulfone (PPSU) enhances viscoelasticity through hydrogen bonding and $\pi\!-\!\pi$ interactions, delaying demixing and suppressing macrovoids while enhancing membrane hydrophilicity and permeability. Alibakhshi et al.^42^ emphasized the kinetic-thermodynamic interplay in polyethersulfone (PES) systems, demonstrating that while high viscoelasticity generally suppresses macrovoids, rapid demixing in PES/NMP solutions can override this effect. Hozumi et al.^43^ provided a rigorous rheological characterization of PVDF/NMP solutions, revealing that PVDF chains behave as polydisperse rigid rods even in the semidilute entangled regime and identifying a universal scaling parameter for viscoelasticity. Tanaka and Stockmayer^44^ proposed that the viscoelastic behavior of semicrystalline polymer solutions originates from multichain junctions formed by microcrystallites, which act as thermoreversible physical cross-links of a transient network and couple gelation directly to crystallization. For PVDF specifically, recent studies further indicate that polymorph selection during NIPS is also coupled to this viscoelastic state. Chan et al.^45^ showed that solvents with higher dipole moments remain longer in the casting film and increase the fraction of polar crystalline phases, while Qiu et al.^46^ proposed that coupled solvent-nonsolvent-polymer interactions can stretch PVDF chains toward the all-trans conformation and thereby promote $\beta$-phase formation. Taken together, these studies suggest that viscoelasticity, modulated by solvent quality, polymer chain entanglement, and microcrystalline cross-links, governs not only the pathway and arrest dynamics of phase separation, but also how strongly solvent and nonsolvent polarity can influence PVDF chain conformation during crystallization.

Recent studies on PVDF-based functional materials further emphasize that nonequilibrium processing pathways can strongly influence chain conformation, dipole alignment, and resulting material properties. In particular, shear-assisted processing and nanofiller-induced interfacial interactions have been shown to promote self-polarization and stabilize the polar $\beta$-phase without external electrical poling^47,48^. Although these works primarily address electromechanical and biofunctional applications, they highlight the broader principle that processing-induced structural evolution in PVDF solutions is governed by the interplay between molecular mobility, intermolecular interactions, and kinetic constraints. This perspective is directly relevant to phase separation processes, where similar nonequilibrium conditions determine not only polymorph selection but also the viscoelastic state of the system and the resulting membrane morphology.

Taken together, the available literature suggests that $T_{d,crit}$ marks not only a morphological transition, but also a switch between two crystallization regimes. At $T_d \le T_{d,crit}$, incomplete dissolution leaves behind residual $\alpha$-polymorph crystalline precursors that increase the viscoelasticity of the polymer solution, promote early gelation during nonsolvent uptake, and favor crystallization through self-seeding and regrowth of the thermodynamically stable $\alpha$-polymorph. In contrast, when $T_d > T_{d,crit}$, these precursors dissolve more completely, the viscoelastic constraint on the system is reduced, and solvent-nonsolvent interactions during demixing exert a stronger influence on polymer chain conformation, thereby promoting the formation of the polar $\beta$-phase. In this view, the fraction of $\alpha$-polymorph, $F_\alpha$, serves not only as a descriptor of the crystalline state of the final membrane but also as a marker of the balance between viscoelastic arrest and conformational selection during phase separation.

These observations suggest that the morphological transition observed near $T_{d,crit}$ is fundamentally linked to the viscoelastic state of the polymer solution during the early stages of phase separation. A theoretical framework capable of describing such behavior is provided by the concept of viscoelastic phase separation (VPS), originally introduced by Tanaka for dynamically asymmetric mixtures^49–51^. In VPS, morphology evolution is governed by the competition between the characteristic deformation time scale generated by phase separation and the stress relaxation time of the slow component of the mixture. When stress relaxation is slow compared to the deformation rate, elastic stresses accumulate within the polymer-rich phase, leading to network-like structures and dynamically arrested morphologies. Conversely, when relaxation is rapid, phase separation proceeds in the classical fluid regime dominated by interfacial tension and diffusion. Recent theoretical work by Yoshimoto and Taniguchi extended this framework to ternary polymer systems relevant to membrane fabrication, explicitly accounting for viscoelastic stresses and stress-diffusion coupling during phase separation^52^.

Despite the conceptual relevance of VPS to membrane formation, most previous studies have either focused on theoretical descriptions or on qualitative interpretations of membrane morphologies in terms of viscoelastic effects. A quantitative experimental link between the rheological properties of polymer casting solutions and the resulting membrane microstructure has remained largely unexplored. In particular, it is not yet clear whether a physically meaningful parameter derived from polymer solution rheology can be used to identify the regime of phase separation that governs membrane formation.

In the present work, we address this question by experimentally investigating the relationship between polymer solution viscoelasticity, crystallization pathway, and membrane morphology during water vapor-induced phase separation (VIPS) of PVDF solutions. To this end, FTIR-ATR spectroscopy of both casting solutions and resulting membranes is combined with scanning electron microscopy (SEM) of the membrane morphology and rheological characterization of the polymer solutions. This combination of techniques allows the crystallization pathway, the viscoelastic state of the solution, and the resulting membrane microstructure to be correlated within a unified mechanistic framework of VIPS of a PVDF/DMSO/water system based on Tanaka’s VPS concept.

Within the VPS framework, the Weissenberg number (*Wi*), is introduced as a dimensionless descriptor linking the relaxation dynamics of the polymer solution to the deformation rate generated during phase separation. Based on the ternary VPS model of Yoshimoto and Taniguchi 52, an expression is derived that allows the Weissenberg number to be estimated from experimentally measurable rheological properties of PVDF solutions. Using this experimentally accessible parameter, three complementary experiments are performed to explore the relationship between solution viscoelasticity and membrane morphology: (i) extended stirring of PVDF solutions at elevated temperatures, (ii) determination of the minimum and critical dissolution temperatures $T_{d,min}$ and $T_{d,crit}$, and (iii) a proof-of-feasibility demonstration of identifying the phase-separation regime governing membrane formation and the associated morphology class. In this way, the present study establishes an experimental connection between polymer solution rheology, dissolution temperature, and membrane morphology within the theoretical VPS framework.

## Theory

### The Weissenberg number in the context of VPS theory

In the context of polymer solution demixing, liquid-liquid phase separation (LLPS) occurs through spinodal decomposition (SD) or nucleation and growth (N&G). This process is described by the fluid model (model H), which contains diffusive dynamics and hydrodynamics. The phase separation morphology is determined by the balance between interfacial forces and viscous forces while ensuring the conservation of momentum. The fluid model assumes the same dynamics for all components of the mixture^53^.

However, if one component is significantly larger in size than the others, e.g., a polymer molecule compared to solvent molecules, the phase separation dynamics change fundamentally. In this case, viscoelasticity plays a crucial role in shaping domain growth. Viscoelastic phase separation (VPS) is defined by the dynamic asymmetry between a fast component (solvent) and a slow component (polymer). Phase separation progresses at an intermediate rate between the characteristic timescales of the fast and slow components. During VPS, the slow component cannot spontaneously respond to the deformation rate induced by the phase separation process itself, which is characterized by the domain deformation time, $\tau _{d}$. Consequently, it exhibits elastic behavior, transitioning to the elastic mode of phase separation. The system’s response to the self-generated deformation rate is given by its slowest mechanical relaxation rate, which is expressed by the slow component’s longest rheological relaxation time, $\tau _{r}$^54^.

The Weissenberg number, $Wi = \tau _{r} \cdot \dot{\gamma }$, is defined as the product of a material’s longest relaxation time, denoted $\tau _{r}$, and the shear rate, denoted $\dot{\gamma }$. The material’s behavior is determined by its ability to relax shear-induced stress. If the material rapidly relaxes the stress, it is considered a liquid with $Wi < 1$. Conversely, if the material does not relax quickly enough, the shear-induced stress accumulates, and the material is considered an elastic solid with $Wi \ge 1$. During VPS, the shear rate is defined as the self-generated mechanical force induced by diffusion and hydrodynamics. The driving forces of the process are the chemical potential gradients between the involved species. Tanaka defines *Wi* as the ratio of the instantaneous deformation rate, $\dot{\gamma }_{inst} \sim \frac{1}{\tau _{d}}$, to the critical stress relaxation rate, $\dot{\gamma }_{crit} \sim \frac{1}{\tau _{r}}$. He emphasizes the competition between stress production and relaxation as the crucial linchpin that switches between fluid phase separation (FPS) mode, otherwise known as liquid-liquid phase separation (LLPS), and elastic phase separation (EPS) mode. In case of $Wi = \frac{\dot{\gamma }_{crit}}{\dot{\gamma }_{inst}} = \frac{\tau _{r}}{\tau _{d}} < 1$, the deformation time of phase separation, $\tau _{d}$, will exceed the relaxation time of the slow component, $\tau _{r}$. The system’s behavior is consistent with that of a viscous fluid, thereby maintaining dynamic symmetry between its components. Domain coarsening proceeds isotropically under the control of interfacial tension, leading to universal, self-similar growth laws. Conversely, when $Wi \ge 1$, deformation occurs at a rate that exceeds the stress relaxation rate. This results in the slow component’s inability to keep pace with the imposed flow. Consequently, the slow component responds elastically. The stress that is accumulated in the network of entangled polymer chains results in the formation of a transient gel. This results in a shift in the force balance from interfacial tension to elasticity, thereby breaking dynamic symmetry. As a result, the universality of self-similar scaling is lost, and domain morphologies become anisotropic, forming elongated, network-like or sponge-like structures characteristic of VPS.

VPS is a three-stage process, as outlined in Table 1. The phase separation process is initiated upon the crossing of the binodal. During the initial VPS stage, $Wi \ll 1$ and $\tau _{r} \ll \tau _{d}$. The slowest viscoelastic relaxation time of the slow component, i.e., the polymer-rich phase, derived from a single-element Maxwell model, $\tau _{r}(\phi ) = \frac{\eta (\phi )}{G'(\phi )}$, scales with polymer concentration. The viscosity of the solvated entangled polymer chains scales as $\eta \sim \phi ^{3.34}$, due to the reptation mechanism. The relaxation modulus has been shown to scale as $G' \sim \phi ^{2.25}$, a consequence of entanglement spacing. The characteristic time of the self-generated domain deformation is expressed as $\tau _{d} \sim \frac{R}{|\nu |}$. The mixture becomes turbid, and after a period of incubation, small polymer-lean domains begin to appear. Their domain radius, *R*, remains negligible, while the composition difference between the two phases, $\Delta \phi$, increases rapidly, due to the expulsion of solvent from the polymer-rich matrix. The flow velocity field is described by the Oseen integral, whose magnitude scales as $|\nu |\approx \frac{k_B T C}{3 \eta \zeta } \Delta \phi ^2$. Therefore, it can be concluded that $\tau _{d} \sim \frac{R}{|\nu |} \sim \frac{1}{\Delta \phi ^2}$, on the basis that $R \approx const \approx \zeta$ (characteristic length) in this stage. In summary, the following proportionalities govern the initial VPS stage: $\tau _{r} \sim \Delta \phi$ and $\tau _{d} \sim \frac{1}{\Delta \phi ^2}$.

The growth of $\Delta \phi$ leads to the intermediate stage of VPS, also known as EPS mode, which is characterized by $Wi \ge 1$ and $\tau _{r} \ge \tau _{d}$. As time progresses, the composition difference $\Delta \phi$ approaches $2 \phi _e$ ($\phi _e$: equilibrium composition). This reduces $|\nu |\approx \frac{k_B T C}{3 \eta \zeta } \Delta \phi ^2$ to $|\nu |\sim \frac{\gamma }{\eta }$ ($\gamma$: interfacial tension), because $\gamma \approx \frac{k_B T C}{3 \zeta } \phi _e^2 \approx const$. Therefore, the deformation time scales linearly with the domain radius $\tau _d \sim \frac{R}{|\nu |} \sim R$. During the intermediate stage, the polymer-lean phase forms expanding domains, while the polymer-rich matrix begins to shrink and stretch, forming a lacy, bicontinuous network. $\phi$ within the polymer-rich phase undergoes only a gradual change due to solvent expulsion into the polymer-lean phase, resulting in $\tau _{r} \approx const$. The polymer-rich phase now displays transient gel-like behavior, wherein polymer chain entanglement and, in case the chemical structure of the polymer enables it, microcrystalline junctions mediate reversible elasticity. The FPS mode transitions to the EPS mode, and the domain growth and deformation are determined by elastic stress rather than interfacial tension, thereby suppressing self-similar growth. Elastic stress is composed of two components: bulk stress, arising from the volume reduction due to solvent expulsion, and shear stress, stemming from structural elongation and rupture.

**Table 1:** Overview of the three stages of viscoelastic phase separation (VPS), summarizing the dominant driving and resisting forces, the prevailing phase separation mechanisms, and the corresponding morphology selectors.

VPS stage	Weissenberg number	Dominant driving term	Dominant resisting term	Mechanism and morphology selector
Initial	$Wi = \frac{\tau _r}{\tau _d} \ll 1$	Thermodynamic: Chemical potential gradients $\nabla \mu$	Viscous drag $\eta \nabla ^2 \nu$	FPS, SD or N&G
Intermediate	$Wi \ge 1$	Elastic: Viscoelastic stress gradients $\nabla \sigma$	Viscous drag $\eta \nabla ^2 \nu$	EPS, anisotropic growth
Late	$Wi \ll 1$	Thermodynamic: Chemical potential gradients $\nabla \mu$	Viscous drag $\eta \nabla ^2 \nu$	FPS, coalescence and Ostwald ripening

The evolution of the microstructure is determined by the balance between stress build-up and stress relaxation, as well as the timing of the dynamic arrest of the polymer-rich phase. If stresses in the transient gel relax prior to arrest, interfacial tension regains control. Self-similar coarsening induces phase inversion, thereby transforming the continuous polymer-rich network into dispersed rounding droplets, while the dispersed polymer-lean phase transitions into a continuous matrix. This corresponds to late stage of VPS, during which $\tau _{d}$ exceeds $\tau _{r}$, resulting in $Wi < 1$ once more. Continued solvent expulsion into the polymer-lean phase effectively vitrifies the polymer-rich phase due to permanent gelation of the supramolecular transient gel network. This nonequilibrium arrest of mobility is referred to as dynamic arrest. If such dynamic arrest occurs prior to stress relaxation, the bicontinuous morphology is preserved. If dynamic arrest occurs subsequent to significant relaxation, phase inversion, coalescence, and Ostwald ripening cause the evolution of the structure towards cellular and eventually nodular. Consequently, the final membrane morphology is determined not solely by the competition between stress build-up and stress relaxation, but also by the timing of dynamic arrest in relation to the onset of phase inversion.

### Measuring the Weissenberg number

In accordance with VPS theory, we propose that the dimensionless Weissenberg number (*Wi*) of a polymer solution indicates whether the early stage of VIPS is governed predominantly by EPS or FPS. Based on this proposition, we examine whether *Wi* can be used to identify the phase-separation regime governing membrane formation and the associated morphology class, i.e., bicontinuous versus nodular. To test this hypothesis, we developed a simple relation to determine *Wi* from the rheometric properties of polymer solutions. The foundation of this relationship is the VPS model for ternary polymer solutions, which was developed by Yoshimoto and Taniguchi^52^. Their model describes the free energy *F* of a ternary polymer system consisting of polymer, solvent, and nonsolvent as a function of the volume fraction of each component and the conformation tensor *W*, according to

1 $$\begin{aligned} F \left[ \phi _P, \phi _S, \phi _W, W \right] = \int _V dr(f_{mix}+f_{int}+f_{ela}) \end{aligned}$$

Here, *W* represents the viscoelastic state of the polymer network that emerges from interactions between polymer chains, including entanglement and microcrystalline junctions. $f_{mix}$ denotes the Flory–Huggins free energy of mixing, $f_{int}$ the interfacial energy, and $f_{ela}$ the elastic energy arising from deformation of the transient gel-like polymer network.

To evolve *W* together with the volume fractions,

2 $$\begin{aligned} \frac{\partial \phi _i}{\partial t} = - \nabla \cdot (\phi _i \nu _i) \end{aligned}$$

Yoshimoto and Taniguchi employ a constitutive equation based on the upper-convected Maxwell model,

3 $$\begin{aligned} \frac{DW}{Dt} = (\nabla \nu _P) \cdot W + (\nabla \nu _P)^T \cdot W - \frac{W - I}{\tau } \end{aligned}$$

where $\frac{D}{Dt} = \frac{\partial }{\partial t} + \nabla \nu _P$ denotes the substantial derivative. The magnitude of the velocity-gradient tensor corresponds to the local shear rate $\Vert \nabla \nu _P \Vert \equiv \dot{\gamma }$. Consequently, the shear terms of *W* are proportional to the local shear rate, $(\nabla \nu _P) \cdot W + (\nabla \nu _P)^T \cdot W \sim \dot{\gamma } W$. Together with the relaxation term $\frac{W - I}{\tau }$, this leads to the definition of the Weissenberg number

4 $$\begin{aligned} Wi = \frac{shear}{relaxation} = \frac{\dot{\gamma } W}{(W - I) / \tau } = \dot{\gamma } \tau \end{aligned}$$

In the present context, the Weissenberg number is interpreted as a dimensionless scaling parameter that characterizes the competition between the stress relaxation time of the polymer-rich phase and the deformation time scale generated during phase separation. Within the VPS framework introduced by Tanaka, morphology selection in dynamically asymmetric mixtures is governed by the ratio between these two time scales. The following derivation therefore does not aim to solve the full set of hydrodynamic equations describing ternary demixing, but instead provides an order-of-magnitude estimate that identifies the regime in which viscoelastic stresses influence phase-separation dynamics.

To estimate the deformation rate generated during phase separation, the hydrodynamic response of the mixture must be considered. The unknown shear rate $\dot{\gamma }$ can therefore be eliminated by using the Stokes equation

5 $$\begin{aligned} 0 = - \nabla p - \sum _{i = P, S} \phi _i \nabla \mu _i + \eta \nabla ^2 \nu + \nabla \sigma \end{aligned}$$

which governs the mean velocity field of the mixture. In this formulation, the thermodynamic chemical potential gradient $\phi \nabla \mu$ represents the driving force of the FPS mode, the viscoelastic stress $\sigma$ represents the elastic response of the polymer network, and viscous dissipation is described by the solvent viscosity term $\eta _S \nabla ^2 \nu$. The viscoelastic stress can be approximated as $\sigma \sim G'(\phi )$, where $G'(\phi )$ denotes the elastic shear modulus of the transient polymer-rich network.

In incompressible mixtures the pressure field primarily acts as a Lagrange multiplier that enforces mass conservation. During phase separation, gradients in chemical potential generate osmotic stresses that drive demixing, while the pressure field adjusts to maintain mechanical equilibrium. For the local scaling estimate used here, these two isotropic contributions are assumed to largely compensate each other within each phase, leaving the approximate balance

6 $$\begin{aligned} \eta \nabla ^2 \nu \approx - \nabla \sigma \end{aligned}$$

To obtain an estimate of the deformation rate, the spatial derivatives in the momentum balance are reduced to characteristic scaling relations using the typical domain size *R* of the evolving phase-separated structure. This yields the relations $\nabla ^2 \nu \sim \frac{|\nu |}{R^2}$ and $\nabla \sigma \sim \frac{G'(\phi )}{R}$, where *R* denotes the characteristic length scale of the developing phase-separated domains.

Insertion into the local balance shows that the velocity is driven by viscoelastic stress and resisted by solvent viscosity, resulting in the scaling relation $|\nu |\sim \frac{G'(\phi ) R}{\eta }$. Using the relation $\dot{\gamma } \sim \frac{|\nu |}{R} \sim \frac{G'(\phi )}{\eta _S}$, the Weissenberg number becomes

7 $$\begin{aligned} Wi = \frac{\tau (\phi ) G'(\phi )}{\eta _s} \end{aligned}$$

Thus, the Weissenberg number can be expressed in terms of the relaxation time $\tau (\phi )$ and the elastic shear modulus $G'(\phi )$ of the polymer solution. Physically, *Wi* represents the ratio between elastic stress generated by the polymer network and viscous dissipation controlled by the solvent viscosity $\eta _S$. When $Wi \ge 1$, viscoelastic stresses relax more slowly than the deformation generated during phase separation, allowing viscoelastic phase separation to dominate the evolution of the microstructure.

In practice, the relaxation time entering the Weissenberg number is estimated from bulk rheological measurements of the polymer solution prior to VIPS. Within the VPS framework, the mechanical response during demixing is dominated by the slowly relaxing polymer-rich phase, which forms a transient viscoelastic network. Although the local rheological properties of this phase evolve as concentration gradients develop during phase separation, these quantities remain experimentally inaccessible. Bulk rheological measurements therefore provide an experimentally accessible proxy for the characteristic relaxation dynamics governing early-stage stress development. The resulting Weissenberg number should therefore be interpreted as a dimensionles regime-classification parameter: conditions with $Wi < 1$ correspond to FPS that typically produces nodular morphologies, whereas $Wi \ge 1$ indicates EPS and the stabilization of bicontinuous structures.

## Methods

Large language model assistance (OpenAI ChatGPT, version GPT-5) was used to support the conceptual development, theoretical discussion, and refinement of the manuscript. All data analysis, interpretation, and final scientific judgments were performed and verified by the authors.

### Materials

Polyvinylidene fluoride (PVDF, Solef 6010, $M_w$ = 300–320 kDa from Solvay, Rheinberg, Germany) was dried at 100 $^{\circ }$C for 24 hours. Dimethyl sulfoxide (DMSO, analytical grade, from Fisher Scientific, Schwerte, Germany) was dried and stored over 3 Å molecular sieve. The unprocessed PVDF exclusively contains the thermodynamically most stable $\alpha$-polymorph and has a degree of crystallinity of approximately 60%^30^. DMSO was used as a solvent for PVDF, and deionized water was used throughout the experiments. All statistical analyses and all modeling were conducted with RStudio v 2023.06.1.

### Preparation of PVDF solutions

DMSO dissolves PVDF only at elevated temperatures of approximately 60 $^{\circ }$C^55^. Our experiments show that the properties of PVDF/DMSO solutions depend not only on the dissolution temperature $T_d$ but also on the dissolution time $t_d$. Within the studied concentration range, a minimum stirring time of 34 minutes is required to obtain a clear and homogeneous solution. This time is defined as the minimum dissolution time $t_{\text {d,min}}$. To ensure reproducibility, all PVDF solutions were therefore prepared at the selected $T_d$ and processed only after reaching $t_{\text {d,min}}$. After removal from the heat source, PVDF/DMSO solutions gradually become turbid due to recrystallization phenomena, typically within about 30 minutes^30^. To minimize variations caused by this time-dependent structural evolution, a maximum processing time $t_{\text {p,max}}$ of 9 minutes was defined for all analytical procedures. The determination of $t_{\text {d,min}}$ and $t_{\text {p,max}}$, including the experimental criteria used to define solution clarity and stability, is described in detail in Supplementary Section S1.1 online.

**Figure 1:**
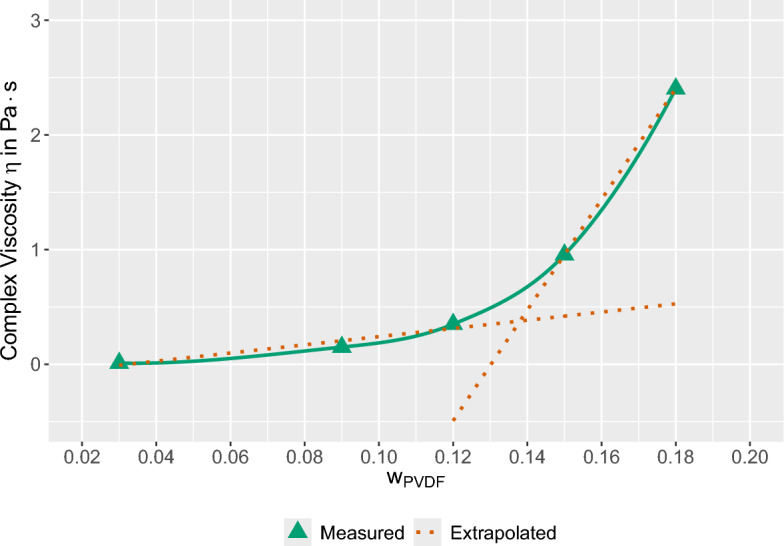
Determination of the overlap concentration $c^*$ of PVDF/DMSO solutions from the viscosity as a function of concentration.

Another crucial property of polymer solutions is the overlap concentration, $c^*$, which is defined as the concentration at which polymer chains in solution begin to overlap and interpenetrate one another, producing topological entanglements that constrain chain motion. At $c < c^*$, solutions are dilute, viscosity scales linearly with concentration, and chains relax rapidly. At $c \simeq c^*$, solutions enter the semidilute unentangled regime, where chains already overlap, friction increases, and viscosity grows faster than linearly. At $c > c^*$, solutions become semidilute entangled, with reptation-controlled dynamics, a significant storage modulus, and the ability to sustain elastic stress. In the context of VPS theory (cf. Section Theory), exceeding $c^*$ is a prerequisite for transient gel formation. The overlap concentration of PVDF/DMSO solutions was estimated to be approximately 14 wt.%, by extrapolating the linear parts of the viscosity curve, as shown in Fig. 1. The impact of $T_d$ on $c^*$ was minimal.

Two methods were employed to prepare PVDF solutions. Method A was selected for the rheological, UV-Vis, and IR investigations. A 40 mL PVDF solution was prepared in a 100 mL three-neck round-bottom flask. The flask was equipped with an overhead stirrer set at a speed of 240 rpm, an inline UV–Vis probe, and an inline PT100 temperature probe. The latter two were connected to a data logger. The sealed flask was placed on a heat-on stage, and the temperature throughout the bulk of the polymer solution was maintained within ± 0.1 $^{\circ }$C. The solution was stirred for 34 min, and all samples were prepared within 9 min before analysis.

The PVDF solutions utilized for membrane preparation were produced according to method B, whereby 50 g of solution was prepared in a 100 mL round-bottom flask with a magnetic stirrer. The solution was stirred at constant temperature overnight and subsequently rested without stirring at $T_d$ for a duration of one hour for degassing. The solution was then cooled to room temperature and used within 30 minutes.

### Preparation of PVDF membranes

Membranes prepared by method A were used for IR analysis, whereas SEM analysis was performed on membranes prepared by method B.

#### Method A

The PVDF solution was cooled to 25 $^{\circ }$C and subsequently poured onto a glass plate measuring $100 \times 100$ mm. A handheld casting knife with a 500 $\upmu \hbox {m}$ gap was utilized to create a homogenous film. The glass plate was then transferred into a vapor box for 4 min with a controlled temperature of 25 $^{\circ }$C and a controlled air stream of 40 L/min with 90 % relative humidity. The vapor box measures $450 \times 320 \times 70$ mm (width x depth x height). Subsequently, the glass plate was immersed in a water bath at room temperature for 30 min, followed by drying at 25 $^{\circ }$C and 10 mbar for 10 min.

#### Method B

The PVDF solution was cooled to room temperature and subsequently poured onto a glass plate measuring $300 \times 250$ mm using a mechanical casting knife that moved at a rate of 5 mm/s, thereby creating a homogenous film of 200 $\mu m$ thickness. The film was then transferred into a vapor box with controlled temperature (21–23 $^{\circ }$C) and air stream (13.7–14.4 L/h). This vapor box measures $350 \times 290 \times 540$ mm (width $\times$ depth $\times$ height). The exposure time and relative humidity were varied according to the experimental requirements. Subsequently, the glass plate was immersed in a water bath at room temperature for a duration of 30 min, thereby quenching any phase separation processes. Subsequently, the water was replaced, and the immersion was continued for a duration of 24 h. The membranes were subjected to freeze-drying at a pressure of 0.11 mbar at a temperature of $-18\;^{\circ }$C for a duration of 24 h.

### UV–Vis spectroscopy

The solutions prepared according to method A were subjected to analysis by UV-Vis spectroscopy. The dissolution process and subsequent stirring at $$T_d$$ were monitored by an UV–Vis spectrometer (Thermo Scientific Evolution 220) between $$\lambda = 250$$–780 nm, with an inline probe, as a function of time. The extinction $E$ at 380 nm was utilized to assess the extent of PVDF dissolution. The transmission $T$ was calculated according to the following equation, with $I$ and $I_0$ being the light intensity after and before passing through the sample.

8 $$\begin{aligned} E = -\lg \frac{I}{I_0} = -\lg T \end{aligned}$$

### Fourier transform infrared spectroscopy with attenuated total reflectance (FTIR-ATR)

The ratio of $\alpha$- to $\beta$-polymorph was measured by FTIR-ATR (Bruker Invenio S) between 4000–400 cm$^{-1}$ in solutions and membranes. The preparation of these solutions and membranes was carried out in accordance with method A. A specific absorption band for the $\alpha$-polymorph was observed at 763 cm$^{-1}$, while the $\beta$-polymorph exhibited a characteristic absorption band at 840 cm$^{-1}$. In the present data set, the FTIR spectra were dominated by the characteristic bands of the $\alpha$- and $\beta$-polymorphs. No additional bands attributable to other polymorphs were resolved with sufficient intensity to warrant quantitative analysis. Therefore, Eq. (9) was used to calculate the apparent fraction of $\alpha$-polymorph, $F_\alpha$, from the absorbance of the 763 and 840 cm$^{-1}$ bands and the absorption coefficients of $\alpha$-form ($K_\alpha$ = 6.1$\cdot$10$^{-4}$ cm$^2$/mol) and $\beta$-form ($K_\beta$ = 7.7$\cdot$10$^{-4}$ cm$^2$/mol), following Li et al.^37^. In the context of this work, $F_\alpha$ is used not only to describe the final membrane crystallography, but also as a proxy for the dominant crystallization pathway during phase separation. The present study found the polymorph fraction in the membrane to be independent from polymer concentration and VIPS duration at a given $T_d$. All membranes that were subjected to IR analyses were produced by VIPS with an exposure time to humid air of 4 min.

9 $$\begin{aligned} F_\alpha =\frac{A_\alpha }{\frac{K_\alpha }{K_\beta }A_\beta +A_\alpha } \end{aligned}$$

### Rheological experiments

The rheological characterization was conducted using a rotational rheometer (Malvern Kinexus Pro). A cone-and-plate measuring system with a diameter of 60 mm was utilized for the measurements. The measuring system was preheated to the temperature of the sample for a period of 5 minutes. Approximately 2 mL of bubble-free sample were applied to the measuring system. Prior to each measurement, a pre-shear interval of 30 s at a shear rate of 5 $s^{-1}$ was executed, followed by a rest interval of 60 s. This protocol was implemented to equalize and to reduce pre-stresses caused by the sample preparation. All rheological data presented in this study was obtained through oscillation measurements. Oscillatory measurement is a method employed to examine viscoelastic materials. Specifically, a shear strain ramp from 1 to 200 % at an angular frequency of $\omega = 10 \, rad/s$ was employed.

Both, the preset shear strain and the measured shear stress, are sinusoidal functions with the same angular frequency. The two functions are phase-shifted by an angle of $\delta$. The tangent of the phase angle $\delta$, otherwise known as the loss factor, is defined as

10 $$\begin{aligned} \tan \delta =\frac{G''}{G'} \end{aligned}$$

The loss modulus, G”, is a measure of the deformation energy dissipated within the sample during the shear process, resulting in irreversible deformation subsequent to the removal of the load. In contrast, the storage modulus G’ is defined as the measure of the deformation energy that is stored by the sample during the shear process. Subsequent to the removal of the load, the stored energy functions as the driving force that reverses the deformation under load, either partially or completely.

The relaxation time, denoted by $\tau$, is calculated using the following equation.

11 $$\begin{aligned} \tau = \frac{G'}{G'' \omega } \end{aligned}$$

All rheological data were measured within the linear viscoelastic range (LVE)^56^. Repeatability was assessed with five independently prepared PVDF solutions. The resulting relaxation times and viscoelastic moduli agreed within $\pm 2.8 \%$, indicating good repeatability of the measurements under the standardized preparation protocol described in Section Preparation of PVDF solutions and Supplementary Section S1.1 online.

### Scanning electron microscopy

Scanning electron microscopy (SEM) micrographs of the membranes were obtained by means of an ESEM Quanta 400 FEG instrument operating at high vacuum. Membrane samples were coated with platinum using a sputter coater (K-550, Emitech Ltd., Kent, UK) at 20 mA for 1 min. Cross-section micrographs were obtained by breaking the samples after cooling with liquid nitrogen.

## Results

### Extended stirring of a PVDF/DMSO solution at elevated temperature

PVDF/DMSO solutions are characterized by a minimum dissolution time, $t_{\text {d,min}}$, that correlates with batch size, and a maximum processing time, $t_{\text {p,max}}$, that is limited to 9 minutes (cf. Supplementary Section S1.1 online). We hypothesized that these solutions are dynamic systems whose properties change over time, even under constant stirring at an elevated temperature apparently sufficient for complete initial dissolution. Therefore, reproducible solution characterization and membrane fabrication depend on defining the time window during which solution properties remain stable.

In order to test this hypothesis, a 230 mL batch of 15 wt.% PVDF in DMSO was prepared, heated to $T_{d} = 50\;^{\circ }C$, and monitored continuously for a period of 200 hours. The batch size was selected to ensure an adequate supply of material for all analyses conducted during the experiment. Temperature and UV–Vis extinction at 380 nm were measured continuously using inline probes. As soon as the solution became visually clear, samples were regularly drawn to determine the Weissenberg number (*Wi*) and the $\alpha$-polymorph crystallite content in both the solution and the membrane. The preparation and analysis of each sample was completed within 9 minutes, thereby meeting the $t_{\text {p,max}}$ condition. The membranes were prepared according to method A.

**Figure 2:**
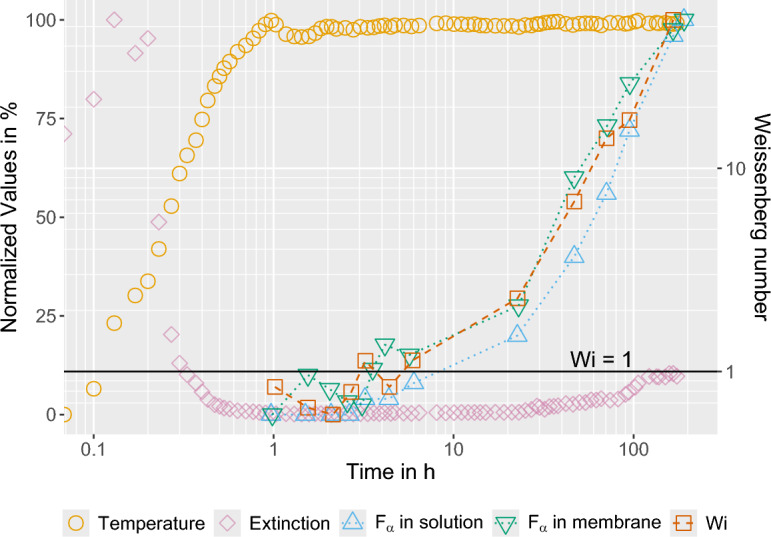
Analysis of a PVDF/DMSO solution heated to 50 $^{\circ }$C and continuously stirred for 200 hours. The measured temperature, extinction, as well as the $\alpha$-polymorph crystallite content in the solution and in the membrane data are plotted as normalized values with respect to the left y-axis. The Weissenberg number data are plotted with respect to the right y-axis.

**Table 2:** Minimum and maximum values used for the normalization of the measured parameters.

Range limits	Temperature $^{\circ }$C	UV–Vis extinction	Solution $\alpha$-polymorph	Membrane $\alpha$/$\beta$ ratio
Minimum	24.5	$-0.007$	0.016	0.548
Maximum	50.2	3.8	0.041	0.752

Figure 2 summarizes the results of the experiment. The entire data set, with the exception of the *Wi* data, was normalized by min–max scaling to facilitate comparison of the different observables. The corresponding minimum and maximum values are listed in Table 2. In accordance with the criteria defined in Supplementary Section S1.1 online, $t_{d,min}$ was 60 minutes and $t_{\text {p,max}}$ was 208 minutes. After 268 minutes, turbidity reappeared as the extinction exceeded the defined threshold value. Accordingly, the solution remained visually clear only between approximately 1 and 4.5 hours. Thereafter, extinction increased continuously, accelerated markedly after 30 hours, and approached a plateau after about 100 hours.

The increase in extinction is attributed to polymer aggregate formation, as evidenced by the concurrent increase in $\alpha$-polymorph crystallites in both the solution and the membranes. We therefore infer that \mbox{$\alpha$-polymorph} microcrystallites surviving the initial dissolution gradually regrow over time through a self-seeding process^57^. Importantly, this observation indicates that the polymorph ratio measured in the final membrane is rooted in the evolving precursor structure of the solution itself. In other words, the increase in $F_\alpha$ does not arise only during final solidification, but already reflects the presence and regrowth of $\alpha$-polymorph crystalline entities in solution that can subsequently bias membrane crystallization toward the thermodynamically stable $\alpha$-phase.

From a thermodynamic perspective, the proposed incomplete initial dissolution of $\alpha$-polymorph microcrystallites can be rationalized by the high energetic stability of ordered PVDF crystallites. Dissolution of such domains requires disruption of a densely packed crystalline arrangement and is therefore energetically unfavorable, despite significant entropy gains, due to a substantial enthalpic cost, which may not be fully compensated when the solvent quality is only marginal and the thermal input is limited. Under these conditions, the most defect-free fraction of the original crystalline entities can persist as microcrystalline precursors. Because the $\alpha$-phase is the thermodynamically most stable polymorph of PVDF under quiescent conditions, these surviving precursors provide a natural template for subsequent regrowth. This interpretation is consistent with the observed increase in $\alpha$-crystallinity in both solution and membrane and supports the view that, below $T_{d,crit}$, polymorph selection is governed primarily by self-seeding and regrowth of residual $\alpha$-type nuclei rather than by conformational forcing during demixing.

*Wi* exhibited a trend similar to that of the $\alpha$-polymorph crystallites. Initially, *Wi* was below unity, but it exceeded 1 after about 3 hours and reached values of approximately 20 after 100 hours. The parallel increase of *Wi* and $\alpha$-crystallinity suggests that both observables are controlled by the same structural evolution, namely the formation and regrowth of microcrystalline junctions. Once $Wi \ge 1$, the solution is able to sustain elastic stress and behaves as a transient gel. In this state, the multichain microcrystalline junctions act not only as crystallization seeds, but also as thermoreversible cross-links that increase the stress-bearing capacity of the polymer-rich phase^ during phase separation processes44,58^.

This observation is central to the interpretation of $F_\alpha$: the same residual $\alpha$-polymorph microcrystalline precursors that promote regrowth of the thermodynamically stable polymorph also increase viscoelasticity and mechanically constrain chain rearrangement. As a consequence, when such junctions are retained, the influence of solvent and nonsolvent polarity on chain conformation during phase separation is attenuated. By contrast, once these junctions are dissolved at higher $T_d$, *Wi* decreases and the polymer chains remain mobile for longer, allowing solvent-nonsolvent interactions to exert a stronger effect on polymorph selection. The gradual increase in *Wi* during prolonged stirring at 50 $^{\circ }$C is therefore consistent with the formation of a self-seeded, \mbpx{$\alpha$polymorph-rich} viscoelastic network under marginal-solvent conditions.

The observed recrystallization requires effective $\Theta$ conditions for polymer chains to undergo collapse and association rather than solvation. The effective $\Theta$ temperature, $\Theta _{\textrm{eff}}$, is the temperature at which a polymer behaves ideally, that is, where attractive and repulsive segment interactions balance, under the actual thermodynamic and kinetic constraints of the system, such as higher than dilute concentration, finite chain length, and viscoelastic coupling. It marks the crossover between good and poor solvent behavior in complex or nonequilibrium polymer systems. It represents a renormalized $\Theta$ point, shifted from the ideal value due to interchain overlap, preferential solvation, and other nonideal effects. The decreasing solubility of PVDF at 50 $^{\circ }$C over the investigated time frame of 200 hours eventually reaches $\Theta _{\textrm{eff}}$ conditions, thereby enabling the gradual recrystallization of polymer chains over time. Partial mobility facilitates local chain reorganization, thereby promoting gradual crystalline domain reformation.

Another factor contributing to recrystallization is shear-induced non-equilibrium dynamics, as introduced by Onuki^59^. The author proposed that shear stress significantly amplifies polymer concentration fluctuations, thereby facilitating the formation of ordered structures even under conditions that are below the equilibrium limits of the bulk. This shear-driven mechanism aligns with the observations made, thereby suggesting that mechanical agitation directly contributes to nucleation and regrowth of $\alpha$-polymorph crystallites.

Our findings demonstrate that PVDF/DMSO solutions manifest significant time-dependent turbidity and an increase of *Wi* near $\Theta _{\textrm{eff}}$ conditions, primarily driven by recrystallization. In addition, apparently initially stable solution properties may turn out to be metastable. This emphasizes the necessity of precisely controlling dissolution time ($t_{d,min}$) and post-dissolution processing time ($t_{\text {p,max}}$) to ensure reproducible solution characterization and membrane preparation.

### Determination of minimum and critical dissolution temperature

The critical dissolution temperature, $T_{d,crit}$, was determined using the methodology established by Li et al.^37^. The $\alpha$-polymorph content in membranes fabricated by VIPS from PVDF solutions prepared in accordance with method A was measured at varying dissolution temperatures. The temperature where a sharp decline in the percentage of $\alpha$-polymorph has been observed was designated as $T_{d,crit}$, denoting the morphological shift from lacy bicontinuous to nodular microstructure. The minimum dissolution temperature, $T_{d,min}$, is associated with the onset of solution homogeneity. At this moment, the solution undergoes a transition from turbid to clear. The quantification of $T_{d,min}$ was accomplished through the utilization of inline UV-Vis spectroscopy. The transmission at 380 nm was measured at various $T_d$ after a minimum dissolution time of 34 min.

**Figure 3:**
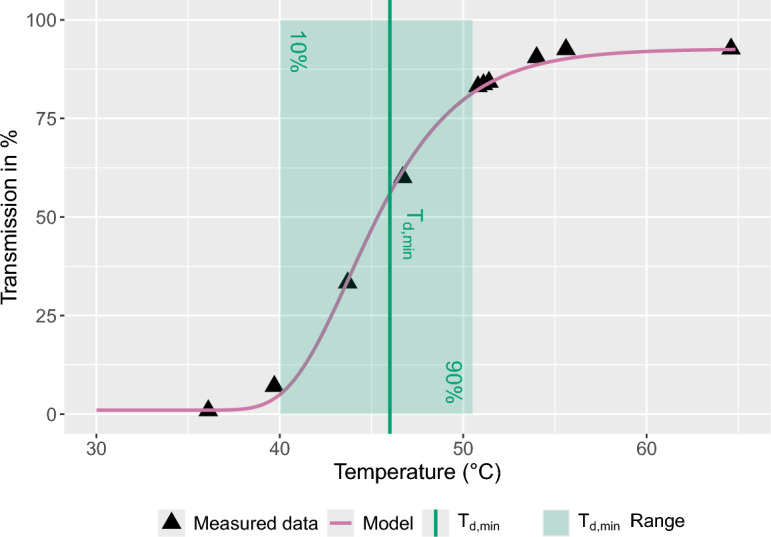
Effect of dissolution temperature $T_d$ on the optical transmission at 380 nm of a 15 wt.% PVDF/DMSO solution. The experimental data were fitted using a modified Gompertz function with a $pseudo R^2$ of 0.998. The $T_d$ range corresponding to a 10–90% increase in transmission is highlighted by a green rectangle. The minimum dissolution temperature $T_{d,min}$ is defined as the temperature at which the transmission has increased by 60%.

The measured data were modeled using the following modified Gompertz function:

12 $$\begin{aligned} y(T)=y_{\text {min}}+(y_{\text {max}}-y_{\text {min}})\cdot \exp (-b\cdot \exp (-k\cdot (T-T_{\text {mdn}}))) \end{aligned}$$

The regression parameters included lower and upper asymptotes, $y_{\text {min}}$ and $y_{\text {max}}$, scale *b*, growth rate *k*, and midpoint temperature $T_{\text {mdn}}$. The goodness of fit was quantified using a pseudo $R^2$ calculated from the ratio of residual sum of squares and total sum of squares. We defined $T_{d,min}$ as the value corresponding to a 60 % increase from the initial transmission. The experimental data of 15 wt.% PVDF in DMSO, as shown in Fig. 3, yielded a $T_{d,min}$ of 46.0 $^{\circ }$C, with a pseudo $R^2$ value of 0.998. Concurrently, transmission increased from 10% to 90% within a temperature interval of 10 $^{\circ }$C. The same analyses of $T_{d,min}$ for 3, 9, 12, and 18 wt.% PVDF are detailed in Supplementary Figs. S2 to S5 online.

**Figure 4:**
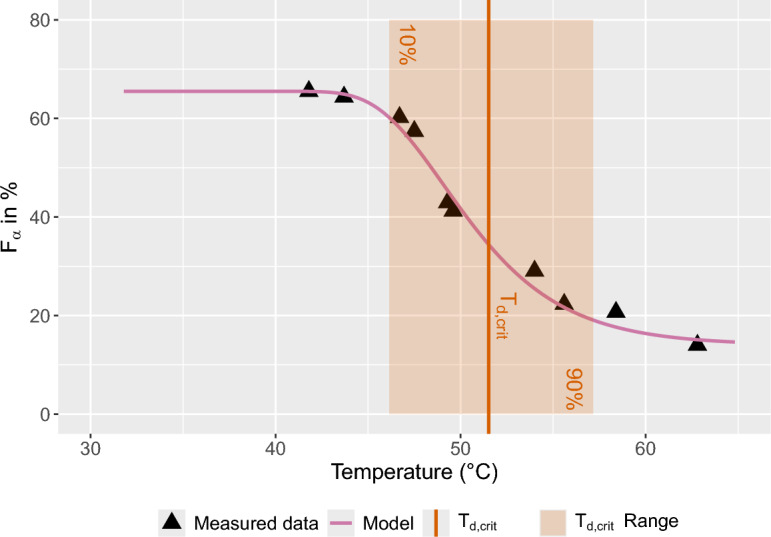
Effect of dissolution temperature $T_d$ on the fraction of $\alpha$-polymorph in a membrane prepared from a 15 wt.% PVDF/DMSO solution. The experimental data were fitted using a modified Gompertz function with a $pseudo R^2$ of 0.986. The $T_d$ range corresponding to a 10–90% decrease in $\alpha$-polymorph content is highlighted by a red rectangle. The critical dissolution temperature $T_{d,crit}$ is defined as the temperature at which the $\alpha$-polymorph content has decreased by 60%.

$T_{d,crit}$ was determined in a similar manner, although it was defined as a 60 % decrease from the initial \mbox{$\alpha$-to-$\beta$} polymorph. For a 15 wt.% PVDF solution, $T_{d,crit}$ was determined to be 51.5 $^{\circ }$C, with a polymorph ratio shift from 10% to 90% within a temperature range of 11 $^{\circ }$C, as illustrated in Fig. 4. The pseudo $R^2$ was found to be 0.985. The same analyses of $T_{d,crit}$ for 3, 9, 12, and 18 wt.% PVDF are detailed in Supplementary Figs. S6 to S9 online.

**Figure 5:**
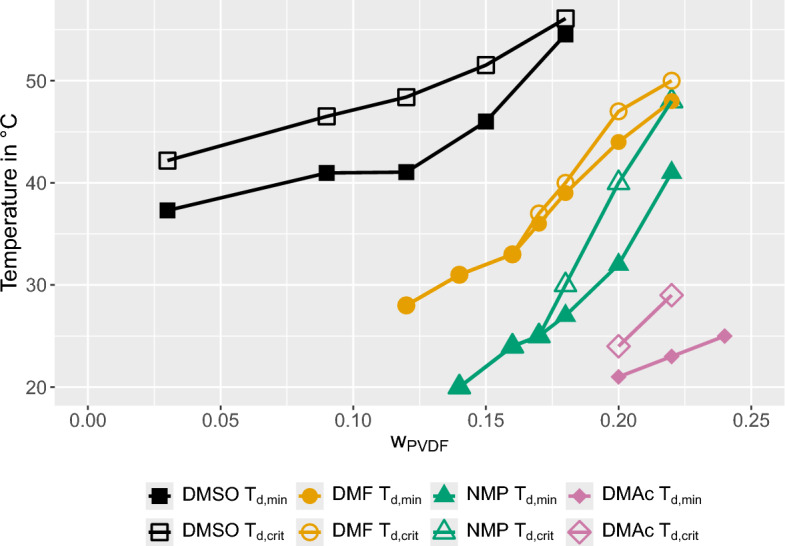
The Minimum and critical dissolution temperatures, $T_{d,min}$ and $T_{d,crit}$, of PVDF in various solvents as a function of polymer mass fraction $w_{PVDF}$. The data for DMSO were obtained in this study, whereas the data for DMF, NMP, and DMAc were taken from^37^.

The results of our determination of $T_{d,min}$ and $T_{d,crit}$ are shown in Fig. 5, alongside the data from Li et al.^37^, who investigated the solvents DMF, NMP, and DMAc. The combined data supports the findings of Li et al.^37^ regarding the impact of solvent quality on $T_{d,min}$ and $T_{d,crit}$. The solvent quality is quantified by the Hansen solubility sphere radius, $R_a$, defined as the distance between the centers of the Hansen spheres for polymer and solvent, and the interaction radius of PVDF, $R_0$^60^, as outlined in Table 3. A larger $R_a$ value indicates a weaker polymer-solvent affinity, whereas an $R_0 > R_a$ signifies poor solvent quality. It was confirmed that DMSO, with an $R_a$ in the vicinity of $R_0$, is close to being a poor solvent for PVDF. Furthermore, a persistent gap between $T_{d,min}$ and $T_{d,crit}$ was observed, creating a $T_d$ window that enables the formation of lacy bicontinuous membrane structures by VIPS.

**Table 3:** Hansen solubility parameters of PVDF^55^, DMSO, DMF, NMP, and DMAc^37^. Calculated Hansen solubility sphere radii $R_a$ of PVDF in the respective solvents and the interaction radius $R_0$ of PVDF^14,20^.

Substance	$\delta _d$	$\delta _p$	$\delta _h$	$R_a$	$R_0$
PVDF	17.2	12.5	9.2		$\approx$ 5
DMSO	18.4	16.4	10.2	4.7	
NMP	18.0	12.3	7.21	2.6	
DMF	17.4	13.7	11.3	2.5	
DMAc	16.8	11.5	10.2	1.6	

**Figure 6:**
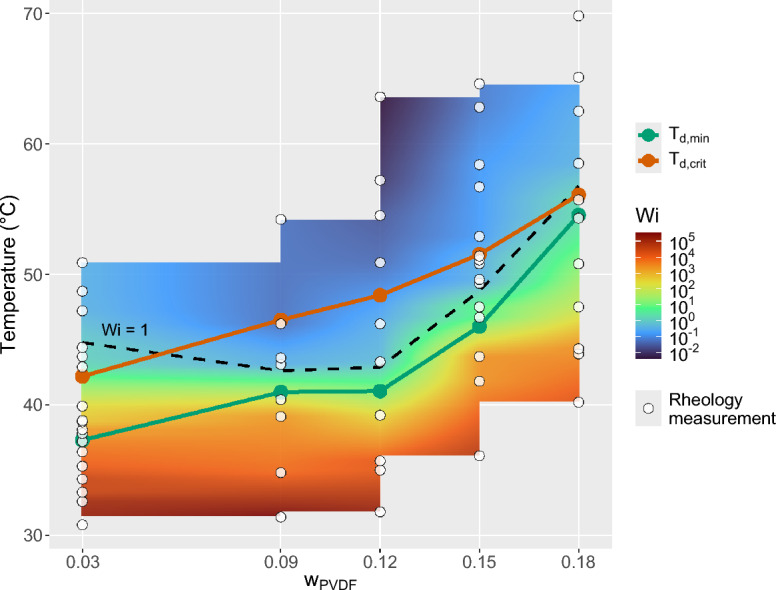
Weissenberg number landscape as a function of polymer mass fraction $w_{PVDF}$ and dissolution temperature $T_d$ of the PVDF/DMSO system. The experimentally determined minimum dissolution temperature $T_{d,\min }$ and critical dissolution temperature $T_{d,crit}$ are superimposed.

Figure 6 summarizes the experimentally determined Weissenberg numbers as a function of the dissolution temperature $T_d$ over a range of polymer mass fractions. Supplementary Figs. S10–S14 show the relationship between *Wi* and $T_d$ separately for 3, 9, 12, 15, and 18 wt.% PVDF. As $T_d$ increases above $T_{d,min}$, *Wi* decreases exponentially from values on the order of $10^4$ toward unity. As long as $Wi = \frac{\tau _r}{\tau _d} \ge 1$, the relaxation time $\tau _r$ exceeds the deformation time $\tau _d$, allowing elastic stresses to accumulate during phase separation and fulfilling the condition for the elastic phase-separation (EPS) mode of VPS^54^. This capacity for elastic stress build-up is associated with polymer-chain entanglement and multichain microcrystalline junctions acting as thermoreversible cross-links^44^. Such junctions may survive incomplete dissolution and, under marginal-solvent conditions, reform over time, as observed for solutions dissolved at 50 $^{\circ }$C (cf. Section Extended stirring of a PVDF/DMSO solution at elevated temperature).

With further increase of $T_d$, these junctions are progressively lost, leading to a reduction in network connectivity and stress-bearing capability. This process is reflected in the continuous decline of *Wi* below unity. At $T_{d,crit}$, which Li et al. 37 associated with the transition to nodular microstructures and a predominance of the $\beta$-polymorph, *Wi* has fallen below unity, indicating that phase separation proceeds predominantly in the fluid phase-separation (FPS) mode where domain evolution is governed mainly by interfacial tension. The condition $Wi \approx 1$ therefore marks a rheological crossover corresponding to the transition from marginal to good solvent conditions, analogous to an effective $\Theta _{\textrm{eff}}$ temperature of the system. This crossover is also relevant for polymorph selection: at low $T_d$, where *Wi* remains high and residual junctions persist, phase separation occurs in a mechanically constrained environment and crystallization proceeds predominantly through self-seeding and regrowth of surviving $\alpha$-type precursors. In contrast, at higher $T_d$, where the junction network has dissolved and *Wi* is low, polymer chains retain greater conformational freedom during demixing, enabling solvent-nonsolvent interactions to bias the chains toward polar conformations and promote $\beta$-phase formation. Consequently, $T_{d,crit}$ represents not only a morphological crossover but also the boundary between two distinct polymorph-selection mechanisms governed by the interplay between thermodynamics (solvent-polymer affinity and crystallite stability) and dynamics (stress relaxation and interfacial forces).

These observations connect the rheological properties of the polymer solution, quantified by the dimensionless experimental *Wi*, with the membrane morphology trends reported by Li et al. ^37^. Membranes prepared within the $T_d$ window predominantly exhibit bicontinuous microstructures with a high fraction of the $\alpha$-polymorph, whereas solutions dissolved above $T_{d,crit}$ produce nodular structures enriched in the $\beta$-polymorph. When these findings are combined with the *Wi* landscape derived from our rheological measurements, the regime boundary $Wi \approx 1$ is found to lie within the experimentally observed $T_d$ window. This correspondence suggests that the viscoelastic state of the polymer solution, quantified by *Wi*, is closely linked to the dominant phase-separation mechanism. In the following section, we therefore examine to what extent the experimentally determined *Wi* can be used as an indicator of the prevailing phase-separation mode, elastic phase separation (EPS) for $Wi \ge 1$ and fluid phase separation (FPS) for $Wi < 1$, and consequently of the general appearance of the membrane microstructure, i.e., bicontinuous versus nodular.

### Mechanism of microstructure evolution during VIPS as a function of the Weissenberg number

#### Effect of polymer concentration and dissolution temperature

In this section we examine whether the experimentally determined Weissenberg number (*Wi*) can be used to identify the phase-separation mode governing membrane formation. Within the framework of viscoelastic phase separation (VPS), the transition between fluid phase separation (FPS) and elastic phase separation (EPS) occurs near $Wi \approx 1$, where the characteristic relaxation time of the polymer-rich phase becomes comparable to the deformation time scale generated during demixing. Two sets of PVDF/DMSO solutions containing 12, 13, 14, and 15 wt.% PVDF were prepared, the first dissolved at $T_d = 50~^\circ$C and the second at $T_d = 70~^\circ$C. All solutions and membranes discussed in this subsection were prepared according to Method B, and the membranes were formed by VIPS exposure for 20 minutes at 80 % relative humidity.

Since the deformation rate during VIPS cannot be measured directly, an experimental rheometric protocol was established to approximate the viscoelastic state of the casting solutions during the initial stages of phase separation. After dissolution at the respective $T_d$, the solution was subjected to a controlled temperature quench from $T_d$ to 25 $^{\circ }$C at a rate of approximately 6 $^\circ$C min$^{-1}$. The temperature was then maintained at 25 $^{\circ }$C for 20 min. During this procedure, the relaxation time of the solution was determined from rheological measurements, allowing the temporal evolution of *Wi* to be estimated.

This rheometric protocol provides an estimate of the initial Weissenberg number $Wi_i$ of the casting solution at the onset of the VIPS process and therefore approximates the viscoelastic state of the system during the early stages of membrane formation. During the actual VIPS process, the local value of *Wi* evolves because the polymer concentration in the polymer-rich phase increases as solvent is expelled, which tends to increase the relaxation time $\tau _r$ and thus *Wi*. At the same time, the driving forces of phase separation, namely the chemical potential gradients $\nabla \mu$ and the viscoelastic stress gradients $\nabla \sigma$, gradually decrease during demixing, reducing the deformation rate and increasing the deformation time $\tau _d$. Consequently, the instantaneous *Wi* within the system is not constant during VIPS. Despite these simplifications, the experimentally determined $Wi_i$ provides a useful regime indicator that characterizes the viscoelastic state of the casting solution at the beginning of phase separation and enables comparison of different preparation conditions.

**Figure 7:**
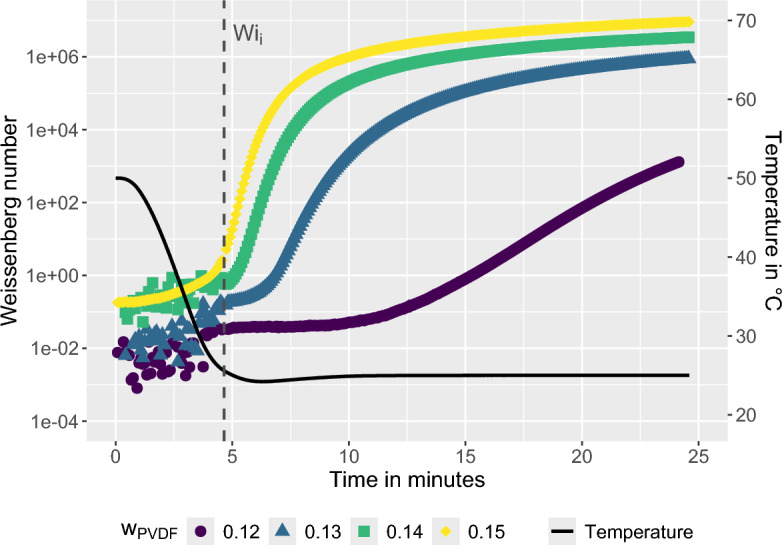
Weissenberg number *Wi* as a function of time for various polymer mass fractions $w_{PVDF}$ during a quench from $T_d$ = 50 $^{\circ }$C to 25 $^{\circ }$C. The initial Weissenberg number $Wi_i$ is defined immediately after the quench.

Figure 7 shows the temporal evolution of *Wi* for solutions prepared at $T_d = 50\,^\circ$C for different polymer mass fractions. The vertical line indicates the initial Weissenberg number $Wi_i$, defined as the value immediately after completion of the temperature quench. This parameter approximates the viscoelastic state of the casting solution at the onset of the VIPS process. For the lower polymer concentrations of 12 and 13 wt.%, $Wi_i$ remains below unity, indicating that phase separation initially proceeds in the FPS regime. With increasing polymer concentration, however, $Wi_i$ exceeds unity. For 14 and 15 wt.% PVDF the solutions therefore enter the EPS regime already at the beginning of VIPS, implying that elastic stresses can accumulate in the polymer-rich phase and influence the evolving microstructure.

**Figure 8:**
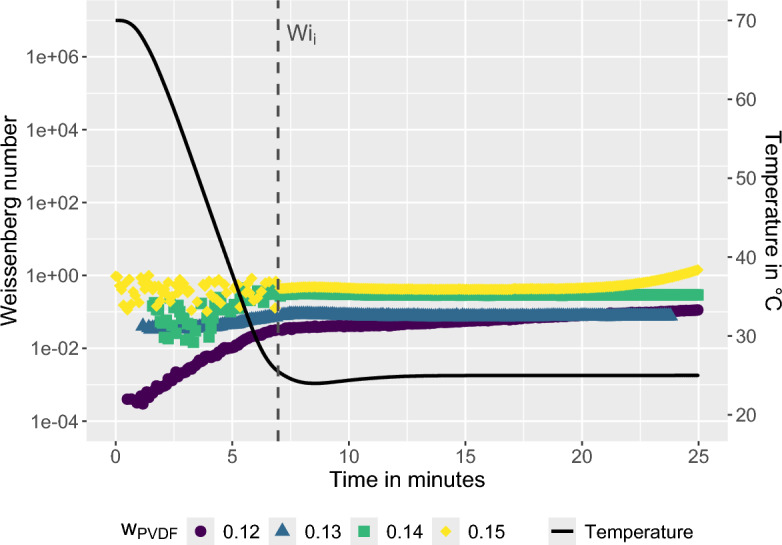
Weissenberg number *Wi* as a function of time for various polymer mass fractions $w_{PVDF}$ during a quench from $T_d$ = 70 $^{\circ }$C to 25 $^{\circ }$C. The initial Weissenberg number $Wi_i$ is defined immediately after the quench.

Figure 8 presents the corresponding evolution of *Wi* for solutions prepared at $T_d = 70\,^\circ$C. In contrast to the previous case, the initial Weissenberg numbers remain below unity for all polymer concentrations. This behavior reflects the reduced viscoelasticity of solutions prepared above the critical dissolution temperature $T_{d,crit}$, where residual microcrystalline junctions are largely dissolved and the polymer chains exhibit greater mobility.

**Figure 9:**
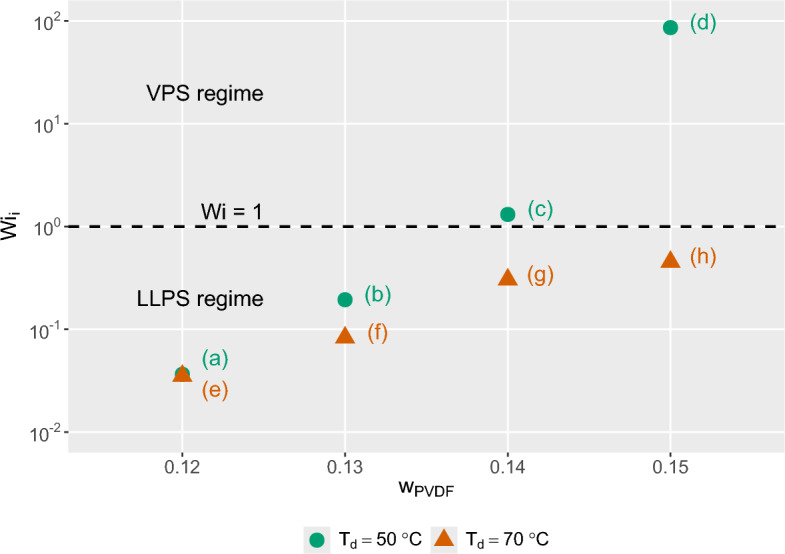
Morphology map relating the initial Weissenberg number $Wi_i$ to polymer mass fraction $w_{PVDF}$ for dissolution temperatures $T_d = 50\,^{\circ }\textrm{C}$ and $70\,^{\circ }\textrm{C}$. The dashed line indicates the threshold $Wi = 1$, which separates FPS ($Wi < 1$) from EPS ($Wi \ge 1$) according to the framework of Tanaka. The labels (a-h) correspond to SEM micrographs of the resulting membrane structures (see Fig. 10). Samples in the $Wi_i \ge 1$ regime exhibit bicontinuous morphologies, whereas samples in the $Wi_i < 1$ regime exhibit predominantly nodular structures.

The dependence of the initial Weissenberg number on polymer concentration and dissolution temperature is summarized in Fig. 9. Irrespective of $T_d$, $Wi_i$ increases monotonically with polymer concentration due to the increasing density of chain entanglements and transient junctions within the solution. However, the absolute values of $Wi_i$ are substantially higher for solutions prepared at $T_d = 50\,^\circ C$ than for those prepared at $T_d = 70\,^\circ C$. This difference reflects the persistence of thermoreversible multichain microcrystalline junctions below $T_{d,crit}$, which enhance the viscoelastic response of the solution.

**Figure 10:**
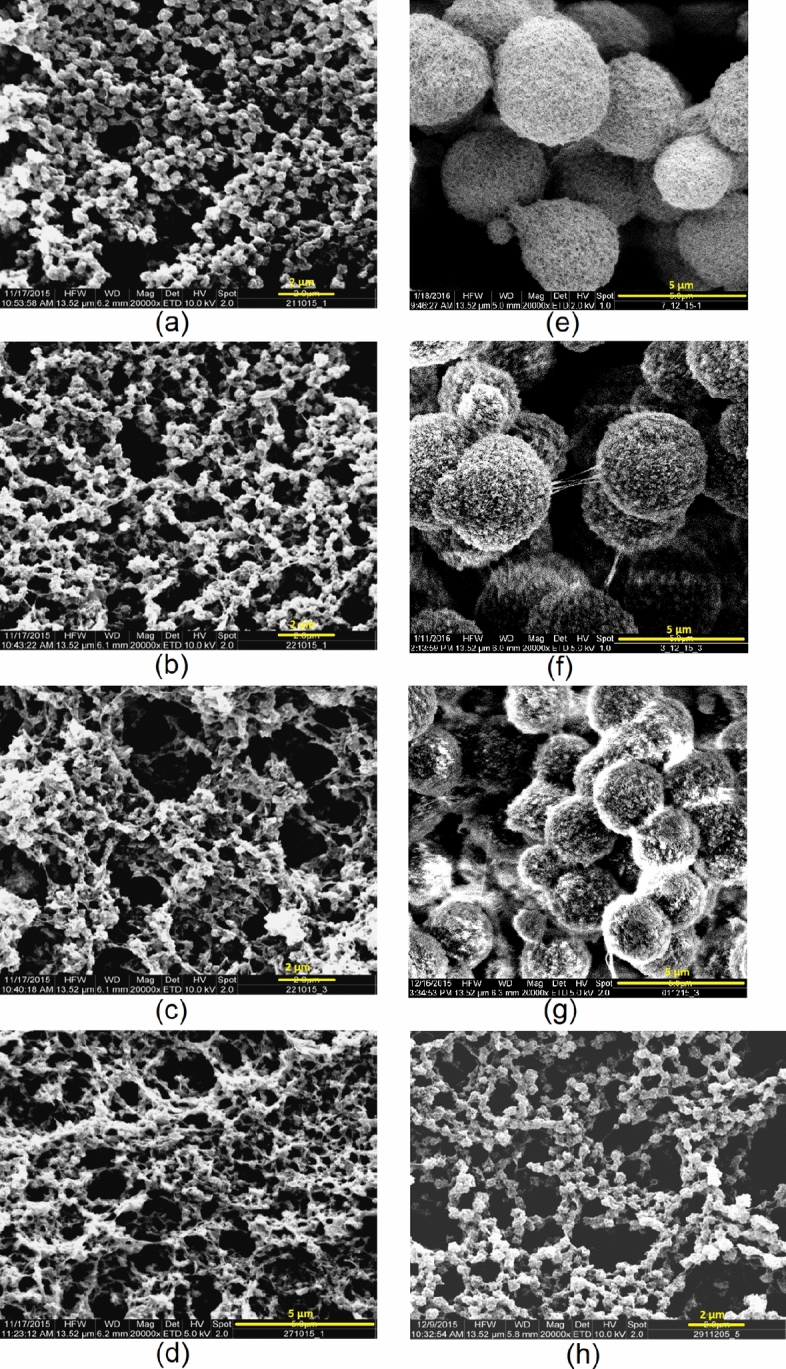
SEM micrographs, at a magnification factor of 20000, show the effect of $T_d$ on the membrane microstructure. Polymer solutions prepared at 50 $^{\circ }$C: (**a**)–(**d**) and 70 $^{\circ }$C: (**e**)–(**h**). The polymer concentration increases top to bottom, ranging from 12 to 15 wt.% PVDF in 1 wt.% increments. VIPS exposure time was 20 minutes at a relative humidity of 80 %.

The morphological consequences of these rheological differences are illustrated by the SEM micrographs in Fig. 10. For solutions prepared at $T_d = 50\,^\circ$C (left column), the membrane microstructure evolves from nodular to bicontinuous with increasing polymer concentration. At 12 wt.% PVDF, densely packed nodules with diameters of approximately 0.4 $\upmu \mathrm{m}$ dominate the structure. At 13 wt.% PVDF the nodules become less densely packed and are connected by longer filaments. A pronounced morphological transition occurs at 14 wt.% PVDF, where the structure changes from nodular to a fine lacy network. At 15 wt.% PVDF the bicontinuous filamentous structure becomes fully developed.

This morphological transition occurs when the initial Weissenberg number reaches $Wi_i \ge 1$, which marks the onset of elastic phase separation according to VPS theory. In this regime, the polymer-rich phase develops a transient stress-bearing network that suppresses self-similar coarsening and stabilizes the evolving bicontinuous microstructure until phase separation is arrested by immersion in the water bath. These observations show that a dissolution temperature $T_d < T_{d,crit}$ is a necessary but not sufficient condition for the formation of a bicontinuous morphology. The additional requirement is that the viscoelastic state of the solution satisfies $Wi_i \ge 1$, enabling elastic stresses to govern the phase-separation dynamics ab initio.

In contrast, membranes prepared from solutions dissolved at $T_d = 70\,^\circ$C (right column of Fig. 10) exhibit nodular structures at all polymer concentrations investigated. The nodule diameter decreases from approximately 4 $\upmu \mathrm{m}$ at 12 wt.% PVDF to about 0.4 $\upmu \mathrm{m}$ at 15 wt.% PVDF. This reduction in characteristic domain size with increasing polymer concentration reflects an earlier dynamic arrest of the FPS coarsening process due to the increasing viscoelasticity of the solution.

Only at 15 wt.% PVDF does *Wi* approach unity during the course of the VIPS process, leading to the appearance of short filamentary connections between the nodules. This observation coincides with the polymer concentration exceeding the overlap concentration ($c^* \approx 14$ wt.%, see Fig. 1), where the formation of transient entanglement networks becomes possible.

In summary, the experimentally determined $$Wi_i$$ correlates directly with the observed membrane microstructures. Solutions with $$Wi_i < 1$$ undergo fluid phase separation and produce nodular membrane morphologies, whereas solutions with $$Wi_i \ge 1$$ enter the regime of elastic phase separation and form bicontinuous filamentous structures. The dissolution temperature further modulates this behavior by controlling the density of residual microcrystalline junctions and thus the viscoelastic state of the casting solution. Together, these results demonstrate that the experimentally determined Weissenberg number $Wi$ is a physically meaningful dimensionless parameter linking polymer solution rheology to the dominant phase-separation regime and the resulting morphology class of the membrane, i.e., bicontinuous versus nodular.

#### Effect of stirring time before membrane casting

The influence of stirring time on membrane morphology was investigated to determine whether the viscoelastic state of the casting solution can evolve during prolonged dissolution, even when the dissolution temperature remains above $T_{d,crit}$. As discussed in Section Extended stirring of a PVDF/DMSO solution at elevated temperature, extended stirring may alter the density of polymer chain entanglements and microcrystalline junctions in the solution. Such structural changes are expected to modify the solution viscoelasticity and therefore the initial Weissenberg number $Wi_i$ that governs the early stages of viscoelastic phase separation during VIPS.

To examine this hypothesis, an additional long-term stirring experiment was performed at $T_d = 70~^\circ$C. In contrast to the experiment described in Section Extended stirring of a PVDF/DMSO solution at elevated temperature, the solutions were not characterized rheologically during stirring. Instead, membranes were prepared after dissolution times of 16, 24, 48, 72, and 120 h. Prior to membrane preparation the solutions were cooled to room temperature and cast according to Method A. The VIPS parameters were 80 % relative humidity and an exposure time of 20 min.

**Figure 11:**
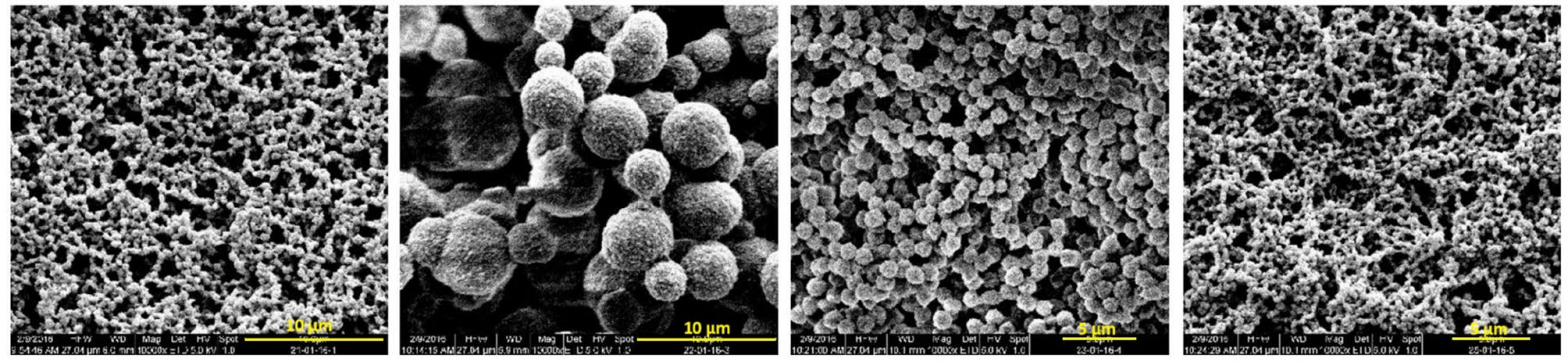
SEM micrographs, at a magnification factor of 10,000, of membrane microstructures prepared from 15 wt.% PVDF dissolved in DMSO at 70 $^{\circ }$C with dissolution times of 24, 48, 72, and 120 hours (from left to right).

Figure 10 (h) shows the SEM micrograph obtained after a dissolution time of $t_d = 16$ hours, while Fig. 11 presents the subsequent morphologies for increasing $t_d$ from left to right. At $t_d = 16$ hours, the microstructure consists of nodules with a diameter of approximately $0.4\,\upmu \mathrm{m}$ that are interconnected by short fibrils. This morphology can be explained by the evolution of the Weissenberg number during VIPS: initially $Wi_i < 1$, followed by a temporary increase of *Wi* above unity (cf. Fig. 8), and finally a decrease below unity again. Consequently, the system passes through all three VPS stages before the coarsening process is dynamically arrested.

After 24 hours of stirring the nodule diameter remains approximately unchanged, but the fibrillar connections disappear. This change indicates a further decrease of $Wi_i$, reducing the influence of viscoelastic stresses during phase separation. After 48 hours, the number of nodules decreases substantially while their diameter increases to approximately $2{-}4\,\upmu \mathrm{m}$. This observation suggests a continued decline of $Wi_i$, leading to a shorter or even absent $Wi \ge 1$ regime during VIPS. Without a transition to EPS, the characteristic domain size increases monotonically until dynamic arrest occurs.

After 72 hours of stirring the number of nodules increases again and their diameter decreases to approximately $1\,\upmu \mathrm{m}$. This change indicates that $Wi_i$ increases again between 48 and 72 hours, allowing a transition to EPS ($Wi \ge 1$) that delays late-stage VPS coarsening. After 120 hours the microstructure becomes indistinguishable from the 24-hour sample, indicating comparable $Wi_i$ conditions.

This non-monotonic evolution of membrane morphology suggests that the viscoelastic state of the casting solution changes dynamically during prolonged stirring. A plausible explanation is that the density of polymer chain entanglements and multichain microcrystalline junctions initially decreases as dissolution continues and the transient network relaxes. At longer stirring times these junctions may gradually reform through self-seeding and nonequilibrium shear dynamics^59^. Because the polymer concentration exceeds the overlap concentration ($c > c^*$), both dissolution and reformation of such junctions are physically plausible.

Consequently, the viscoelastic properties of the solution, and thus the effective $Wi_i$ governing the early stages of VIPS, may decrease initially and subsequently increase again. These observations support the interpretation that the experimental Weissenberg number and the associated density of transient junctions in the casting solution are dynamic quantities that can evolve even under apparently constant dissolution conditions. The resulting changes in the balance between stress build-up and relaxation during phase separation directly influence the membrane morphology obtained by VIPS.

#### Effect of VIPS duration

**Figure 12:**
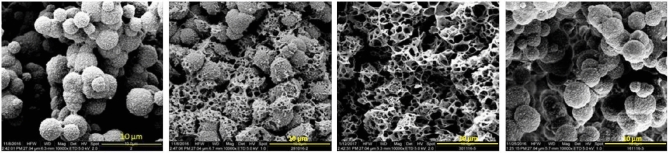
SEM micrographs, at a magnification factor of 10,000, of membrane microstructures prepared from 15 wt.% PVDF dissolved in DMSO at 90 $^{\circ }$C with VIPS exposure times of 6, 21, 41, and 61 minutes (from left to right).

The influence of extended VIPS exposure on membrane morphology was examined for solutions prepared at $T_d > T_{d,crit}$, where the initial Weissenberg number is well below unity ($Wi_i \ll 1$). Figure 12 shows SEM micrographs of membranes prepared from a 15 wt.% PVDF solution dissolved in DMSO at $T_d = 90\,^{\circ }$C. The VIPS exposure times were 6, 21, 41, and 61 minutes at a relative humidity of 80 %.

The selected dissolution temperature ensures $Wi_i \ll 1$, such that VIPS initially proceeds in the FPS mode of the initial VPS stage. In this regime, dynamic symmetry prevails and the interplay between interfacial tension and viscous dissipation selects a characteristic length scale that follows classical coarsening laws.

After 6 minutes of exposure, the membrane microstructure consists of dispersed nodules with diameters between 2 and 4 $\upmu \mathrm{m}$. This morphology suggests that either $Wi < 1$ persists throughout the VIPS process, leading to continuous fluid phase separation and subsequent phase inversion and coarsening, or that a temporary $Wi > 1$ regime occurs but is too brief to significantly delay late-stage VPS. This initial droplet structure defines the geometric baseline that constrains all subsequent intra-droplet processes.

When the VIPS duration is extended to 21 min, the overall nodule diameter remains largely unchanged. However, approximately half of the nodules lose their outer skin and develop a bicontinuous lacy substructure. This observation suggests that after the initial FPS-driven droplet formation, continued solvent expulsion from the polymer-rich droplets into the surrounding polymer-lean phase increases the concentration difference $\Delta \phi$. Because the relaxation time scales as $\tau _r \sim \Delta \phi$ and the deformation time as $\tau _d \sim \frac{1}{\Delta \phi ^2}$, the Weissenberg number increases according to $Wi = \frac{\tau _r}{\tau _d}$ until $Wi \ge 1$.

Once this threshold is reached, the intra-droplet phase separation transitions from FPS mode to EPS mode. The emergence of viscoelastic stress gradients $\nabla \sigma$ introduces an additional driving force for the intermediate VPS stage. Morphological evolution within the droplets then proceeds by anisotropic growth of a viscoelastic network while mass and momentum remain conserved at the droplet scale. Under otherwise identical conditions but at $T_d = 70\,^{\circ }$C, significantly smaller nodules with diameters of approximately 0.4 $\upmu \mathrm{m}$ were observed (cf. Fig. 10 (h) ). This difference can be attributed to the onset of the intermediate VPS stage prior to phase inversion, so that phase inversion occurs only during the late stage of VPS.

After 41 minuntes of VIPS exposure, all nodules become skinless and contain a bicontinuous lacy substructure. At the same time, the polymer-lean domains inside the droplets grow in size while their number decreases. This behavior reflects continued solvent expulsion into the polymer-lean phase, which increases $\Delta \phi$ and causes contraction of the elastic polymer-rich network. The resulting morphology indicates that the system approaches the onset of late-stage VPS.

Between 41 and 61 minutes of exposure, the viscoelastic stresses within the bicontinuous substructure relax. This relaxation leads to intra-droplet phase inversion and coalescence of the polymer-rich sub-droplets. Consequently, the droplet diameter returns to the 2–4 $\upmu \mathrm{m}$ length scale already observed after 6 minutes of exposure. This recovery may result either from insufficient time for inter-droplet coalescence and Ostwald ripening before the water-bath quench or from dynamic arrest of the evolving structure.

In summary, the Weissenberg number, and therefore the ability of the solution to form a transient gel through chain entanglements and thermoreversible microcrystalline junctions, is a key parameter governing membrane morphology during VIPS. Traditional descriptions of VIPS focus on the interplay between liquid-liquid demixing and polymer crystallization, but do not explicitly account for viscoelasticity. Viewed through the framework of VPS theory, the VIPS process is governed by the dynamic transition between fluid (FPS) and elastic (EPS) modes of phase separation.

When $T_d < T_{d,crit}$, VIPS begins in the intermediate VPS stage because $Wi_i \ge 1$. Elastic stresses can therefore build up immediately, stabilizing emerging bicontinuous network structures that become dynamically arrested before phase inversion occurs. In contrast, when $T_d > T_{d,crit}$, VIPS starts in the FPS mode of the initial VPS stage because $Wi_i < 1$. Depending on the process conditions, such as dissolution temperature, polymer concentration, and VIPS duration, two different phase-separation pathways may arise.

In the first scenario, the initial lacy network undergoes phase inversion and evolves toward an increasingly nodular morphology that coarsens through interfacial-tension-driven mechanisms until solvent expulsion ultimately causes dynamic arrest. In the second scenario, continued solvent expulsion increases *Wi* sufficiently to trigger the intermediate VPS stage. This transition delays late-stage coarsening and leads to smaller nodules compared with the first scenario.

This mechanistic interpretation connects the classification proposed by Li et al.^37^, who distinguished between “non-crystallization-gelling” at $T_d < T_{d,crit}$ and “crystallization-gelling” at $T_d > T_{d,crit}$, to the elastic and fluid phase-separation modes predicted by VPS theory. The VPS framework also provides a rationale for the observations of Annamalai et al.^38^, who reported that gelation occurs earlier at $T_d < T_{d,crit}$ than at $T_d > T_{d,crit}$. In VPS terms, early gelation corresponds to the EPS regime, whereas late gelation reflects a prolonged FPS regime in which viscoelastic effects emerge only after $Wi \ge 1$ is reached.

The resulting bicontinuous or nodular membrane morphologies, network arrest versus droplet coarsening, can therefore be interpreted as the outcome of a competition between viscoelastic stress build-up and stress relaxation relative to the timing of dynamic arrest. The experimental Weissenberg number thus serves as effective dimensionless parameter elucidating the mechanistic sequence of VIPS and distinguishing conditions that favor nodular or bicontinuous membrane structures.

## Conclusion

This study establishes a mechanistic framework for porous PVDF membrane formation by nonsolvent vapor-induced phase separation (VIPS), based on the interplay between dissolution temperature, solution viscoelasticity, and phase-separation dynamics. We identified the minimum dissolution temperature, $T_{d,min}$, and the critical dissolution temperature, $T_{d,crit}$, for PVDF/DMSO solutions across a range of polymer concentrations. These parameters define a dissolution-temperature window in which PVDF/DMSO solutions exhibit significant elasticity arising from polymer chain entanglements and thermoreversible microcrystalline junctions. The resulting supramolecular network behaves as a transient gel capable of resisting deformation during the VIPS process.

Within Tanaka’s viscoelastic phase separation (VPS) theory, this elastic response corresponds to the elastic phase separation (EPS) mode. If the stresses that develop in the polymer-rich phase cannot relax before dynamic arrest occurs, caused by solvent expulsion into the polymer-lean phase, the evolving lacy bicontinuous structure is preserved in the final membrane morphology. When $T_{d,crit}$ is exceeded, dissociation of the microcrystalline junctions reduces the elasticity of the polymer-rich phase. Phase separation then proceeds predominantly via the fluid phase separation (FPS) mode of VPS, in which interfacial tension and viscous flow govern morphological evolution, leading to phase inversion and coarsening prior to dynamic arrest and ultimately to nodular membrane structures.

At the same time, $T_{d,crit}$ marks a transition in the dominant crystallization mechanism. Below this threshold, surviving $\alpha$-type crystalline precursors and the associated viscoelastic network promote self-seeding and regrowth of the thermodynamically stable $\alpha$-polymorph. Above $T_{d,crit}$, the reduced viscoelastic constraint allows solvent–nonsolvent interactions during demixing to influence chain conformation more strongly, thereby favoring formation of the polar $\beta$-phase.

The experimental Weissenberg number (*Wi*), derived from rheological measurements of the casting solution, provides a quantitative dimensionless indicator of the balance between stress build-up and stress relaxation. It distinguishes between EPS conditions ($Wi \ge 1$) and FPS conditions ($Wi \ll 1$) and therefore identifies the dominant phase-separation regime during membrane formation. Solutions prepared at $T_d < T_{d,crit}$ exhibit early gelation associated with the EPS regime and preserve bicontinuous structures. In contrast, solutions prepared at $T_d > T_{d,crit}$ experience extended FPS behavior followed by only a brief EPS period, leading to nodular morphologies.

Together, these results demonstrate that dissolution conditions and rheological properties of the polymer solution determine the phase-separation pathway during VIPS and thereby control the resulting membrane microstructure. By integrating these observations within the VPS framework, this work unifies previous empirical interpretations of PVDF membrane formation into a consistent mechanistic picture. The identification of $T_{d,min}$, $T_{d,crit}$, and *Wi* as key control parameters provides practical guidance for tuning membrane structure and performance.

Several limitations of the present study should nevertheless be noted. The experimental Weissenberg number derived here represents an estimate based on bulk rheological measurements of the casting solution prior to phase separation. During VIPS, the polymer concentration and relaxation dynamics of the polymer-rich phase evolve continuously as solvent is expelled and nonsolvent is absorbed, so the local value of *Wi* may change during demixing and cannot currently be measured directly. Furthermore, the present analysis focuses on the PVDF/DMSO system within a limited range of experimental conditions, so the quantitative values of $T_{d,min}$, $T_{d,crit}$, and *Wi* may not be directly transferable to other polymer–solvent systems.

Future work should therefore aim to probe the evolving rheological properties of the polymer-rich phase during phase separation more directly and to extend the present approach to additional semicrystalline polymers and solvent systems. Combining rheological measurements with in-situ structural probes such as time-resolved scattering or spectroscopic techniques could provide deeper insight into the coupling between crystallization, viscoelastic stress development, and phase-separation dynamics.

Despite these remaining challenges, the present results demonstrate that the viscoelastic state of the casting solution provides a physically meaningful link between dissolution conditions and membrane morphology. By connecting dissolution temperature, transient microcrystalline junction networks, solution rheology, and membrane microstructure within the VPS framework, this work establishes a mechanistic basis for understanding and controlling membrane formation from semicrystalline polymer solutions and offers a foundation for more predictive design of advanced porous membranes.

## Supplementary Information

Supplementary Information.

## Data Availability

The datasets generated during and/or analyzed during the current study are available in the OSF repository, under the following link: https://doi.org/10.17605/OSF.IO/B52FA. Data are available under the terms of the Creative Commons Attribution 4.0 International license (CC-BY 4.0).

## References

[1] Developments in crystalline polymers-1 (ed Bassett, D.C.) ISBN: 978-94-009-7343-5. 10.1007/978-94-009-7343-5 (Springer, Dordrecht, 1982).

[2] Dong, Y. et al. Synergistic effect of PVDF-coated PCL-TCP scaffolds and pulsed electromagnetic field on osteogenesis. *Int. J. Mol. Sci.*10.3390/ijms22126438 (2021). 34208563 10.3390/ijms22126438 PMC8234164

[3] Liu, F., Hashim, N. A., Liu, Y., Abed, M. M. & Li, K. Y. Progress in the production and modification of PVDF membranes. *J. Membr. Sci.* **375**, 1–27. 10.1016/j.memsci.2011.03.014 (2011) (**(ISSN: 03767388)**).

[4] Kang, G.-D. & Cao, Y.-M. Application and modification of poly(vinylidene fluoride) (PVDF) membranes–A review. *J. Membr. Sci.* **463**, 145–165. 10.1016/j.memsci.2014.03.055 (2014) (**(ISSN: 03767388)**).

[5] Membrane fabrication (ed Hilal, N.) ISBN: 1482210452 (CRC Press, Boca Raton, 2015).

[6] Mulder, M. Basic principles of membrane technology 2. ed. ISBN: 0-7923-4247-x (Kluwer, Dordrecht, 1996).

[7] Lalia, B. S., Kochkodan, V., Hashaikeh, R. & Hilal, N. A review on membrane fabrication: Structure, properties and performance relationship. *Desalination* **326**, 77–95. 10.1016/j.desal.2013.06.016 (2013).

[8] Ismail, N. et al. Investigating the potential of membranes formed by the vapor induced phase separation process. *J. Membr. Sci.* **597**, 117601. 10.1016/j.memsci.2019.117601 (2020).

[9] Bohr, S. J. et al. State-of-the-art review of porous polymer membrane formation characterization–How numerical and experimental approaches dovetail to drive innovation. *Front. Sustain.*10.3389/frsus.2023.1093911 (2023).

[10] Khayet, M. Membranes and theoretical modeling of membrane distillation: A review. *Adv. Colloid Interface Sci.* **164**, 56–88. 10.1016/j.cis.2010.09.005 (2011). 21067710 10.1016/j.cis.2010.09.005

[11] Eykens, L., de Sitter, K., Dotremont, C., Pinoy, L. & van der Bruggen, B. How to optimize the membrane properties for membrane distillation—A review. *Ind. Eng. Chem. Res.* **55**, 9333–9343. 10.1021/acs.iecr.6b02226 (2016).

[12] Eykens, L., de Sitter, K., Dotremont, C., Pinoy, L. & van der Bruggen, B. Membrane synthesis for membrane distillation: A review. *Separat. Purif. Technol.* **182**, 36–51. 10.1016/j.seppur.2017.03.035 (2017).

[13] Rezaei, M. et al. Wetting phenomena in membrane distillation: Mechanisms, reversal, and prevention. *Water Res.* **139**, 329–352. 10.1016/j.watres.2018.03.058 (2018). 29660622 10.1016/j.watres.2018.03.058

[14] Jung, J. T. et al. Understanding the non-solvent induced phase separation (NIPS) effect during the fabrication of microporous PVDF membranes via thermally induced phase separation (TIPS). *J. Membr. Sci.* **514**(250–263), 03767388. 10.1016/j.memsci.2016.04.069 (2016).

[15] Marshall, J. E. et al. On the solubility and stability of polyvinylidene fluoride. *Polymers.*10.3390/polym13091354 (2021). 33919116 10.3390/polym13091354 PMC8122610

[16] Figoli, A. et al. Towards non-toxic solvents for membrane preparation: A review. *Green Chem.* **16**, 4034. 10.1039/C4GC00613E (2014).

[17] Marino, T., Blefari, S., Di Nicolò, E. & Figoli, A. A more sustainable membrane preparation using triethyl phosphate as solvent. *Green Process. Synthesis* **6**, 295–300. 10.1515/gps-2016-0165 (2017).

[18] Meringolo, C. et al. Tailoring PVDF membranes surface topography and hydrophobicity by a sustainable two-steps phase separation process. *ACS Sustain. Chem. Eng.* **6**, 10069–10077. 10.1021/acssuschemeng.8b01407 (2018).

[19] Su, J. F., Beltsios, K. G., Li, P.-H. & Cheng, L.-P. Facile formation of symmetric microporous PVDF membranes via vapor-induced phase separation of metastable dopes. *Colloids Surfaces A Physicochem. Eng. Aspects* **634**, 128012. 10.1016/j.colsurfa.2021.128012 (2022).

[20] Xie, W. et al. Toward the next generation of sustainable membranes from green chemistry principles. *ACS Sustain. Chem. Eng.* **9**, 50–75. 10.1021/acssuschemeng.0c07119 (2021).

[21] Russo, F. et al. Advancements in sustainable PVDF copolymer membrane preparation using Rhodiasolv PolarClean as an alternative eco-friendly solvent. *Clean Technol.* **3**, 761–786. 10.3390/cleantechnol3040045 (2021).

[22] Tazaki, M., Wada, R., Abe, M. O. & Homma, T. Crystallization and gelation of poly(vinylidene fluoride) in organic solvents. *J. Appl. Polym. Sci*. **65**, 1517–1524. (1997) 10.1002/(SICI)1097-4628(19970822)65:8%3C1517::AID-APP9%3E3.0.CO;2-J

[23] Dikshit, A. K. & Nandi, A. K. Thermoreversible gelation of poly(vinylidene flouride) in diethyl adipate: A concerted mechanism. *Macromolecules* **31**, 8886–8892. 10.1021/ma980764n (1998).

[24] Cheng, L.-P., Lin, D.-J., Shih, C.-H., Dwan, A.-H. & Gryte, C.C. (1999) PVDF membrane formation by diffusion-induced phase separation-morphology prediction based on phase behavior and mass transfer modeling. *J. Polym. Sci. Part B Polym. Phys*. **37**, 2079–2092. 10.1002/(SICI)1099-0488(19990815)37:16%3C2079::AID-POLB11%3E3.0.CO;2-Q

[25] Li, X. et al. Evolution of polyvinylidene fluoride (PVDF) hierarchical morphology during slow gelation process and its superhydrophobicity. *ACS Appl. Mater. Interfaces* **5**, 5430–5435. 10.1021/am401412a (2013). 23725003 10.1021/am401412a

[26] Khayet, M., Cojocaru, C. & García-Payo, M. C. Experimental design and optimization of asymmetric flat-sheet membranes prepared for direct contact membrane distillation. *J. Membr. Sci.* **351**, 234–245. 10.1016/j.memsci.2010.01.057 (2010).

[27] Tan, X. & Rodrigue, D. A Review on porous polymeric membrane preparation. Part I: Production techniques with polysulfone and poly(vinylidene fluoride). *Polymers*. 10.3390/polym11071160 (2019). 10.3390/polym11071160 PMC6680680 31288433

[28] Tsai, J. T. et al. Retainment of pore connectivity in membranes prepared with vapor-induced phase separation. *J. Membr. Sci.* **362**, 360–373. 10.1016/j.memsci.2010.06.039 (2010).

[29] Mousavi, S. M. & Zadhoush, A. Investigation of the relation between viscoelastic properties of polysulfone solutions, phase inversion process and membrane morphology: The effect of solvent power. *J. Membr. Sci.* **532**, 47–57. 10.1016/j.memsci.2017.03.006 (2017).

[30] Alexowsky, C. *Herstellung von porösen Polyvinylidenfluorid-Membranen mit maßgeschneiderten Eigenschaften durch schnelle und skalierbare dampfinduzierte Phasentrennung Dissertation* (Universität Duisburg-Essen, Essen, 2019).

[31] Alexowsky, C., Bojarska, M. & Ulbricht, M. Porous poly(vinylidene fluoride) membranes with tailored properties by fast and scalable non-solvent vapor induced phase separation. *J. Membr. Sci.* **577**, 69–78. 10.1016/j.memsci.2019.01.033 (2019).

[32] Benz, M., Euler, W. B. & Gregory, O. J. The influence of preparation conditions on the surface morphology of poly(vinylidene fluoride) films. *Langmuir* **17**, 239–243. 10.1021/la001206g (2001).

[33] Lin, D.-J., Beltsios, K. G., Young, T.-H., Jeng, Y.-S. & Cheng, L.-P. Strong effect of precursor preparation on the morphology of semicrystalline phase inversion poly(vinylidene fluoride) membranes. *J. Membr. Sci.* **274**, 64–72. 10.1016/j.memsci.2005.07.043 (2006).

[34] Wang, X., Wang, X., Zhang, L., An, Q. & Chen, H. Morphology and formation mechanism of poly(vinylidene fluoride) membranes prepared with immerse precipitation: Effect of dissolving temperature. *J. Macromol. Sci. Part B* **48**, 696–709. 10.1080/00222340902958950 (2009).

[35] Gugliuzza, A. & Drioli, E. New performance of hydrophobic fluorinated porous membranes exhibiting particulate-like morphology. *Desalination* **240**, 14–20. 10.1016/j.desal.2008.07.007 (2009).

[36] Li, M. et al. Controlling the microstructure of poly(vinylidene-fluoride) (PVDF) thin films for microelectronics. *J. Mater. Chem. C* **1**, 7695. 10.1039/C3TC31774A (2013).

[37] Li, C.-L. et al. Insight into the preparation of poly(vinylidene fluoride) membranes by vapor-induced phase separation. *J. Membr. Sci.* **361**, 154–166. 10.1016/j.memsci.2010.05.064 (2010).

[38] Annamalai, P. K. et al. Kinetics of mass transfer during vapour-induced phase separation (VIPS) process and its influence on poly-(vinylidene fluoride) (PVDF)membrane structure and surface morphology. *Desalination Water Treatment* **34**, 204–210. 10.5004/dwt.2011.2809 (2011).

[39] Hung, W.-L., Wang, D.-M., Lai, J.-Y. & Chou, S.-C. On the initiation of macrovoids in polymeric membranes—Effect of polymer chain entanglement. *J. Membr. Sci.* **505**, 70–81. 10.1016/j.memsci.2016.01.021 (2016).

[40] Su, S.-L., Wang, D.-M. & Lai, J.-Y. Critical residence time in metastable region—A time scale determining the demixing mechanism of nonsolvent induced phase separation. *J. Membr. Sci.* **529**, 35–46. 10.1016/j.memsci.2017.01.059 (2017).

[41] Feng, Y. et al. Rheology and phase inversion behavior of polyphenylenesulfone (PPSU) and sulfonated PPSU for membrane formation. *Polymer* **99**, 72–82. 10.1016/j.polymer.2016.06.064 (2016).

[42] Alibakhshi, S., Youssefi, M., Hosseini, S. S. & Zadhoush, A. Significance of thermodynamics and rheological characteristics of dope solutions on the morphological evolution of polyethersulfone ultrafiltration membranes. *Polym. Eng. Sci.* **61**, 742–753. 10.1002/pen.25613 (2021).

[43] Hozumi, H., Nohara, Y., Horikawa, Y. & Shikata, T. Rigid rod particle like viscoelastic responses of poly(vinylidene fluoride) in N -methylpyrrolidone solution. *J. Rheol.* **67**, 683–692. 10.1122/8.0000610 (2023).

[44] Tanaka, F. & Stockmayer, W. H. Thermoreversible gelation with junctions of variable multiplicity. *Macromolecules* **27**, 3943–3954. 10.1021/ma00092a039 (1994).

[45] Chan, K.-Y., Li, C.-L., Wang, D.-M. & Lai, J.-Y. Formation of porous structures and crystalline phases in poly(vinylidene fluoride) membranes prepared with nonsolvent-induced phase separation-roles of solvent polarity. *Polymers* **15**, 1314. 10.3390/polym15051314 (2023). 36904555 10.3390/polym15051314 PMC10007550

[46] Qiu, Z., Lei, T., Li, S., Cai, X. & Yang, K. Insights into polymorphic transition of PVDF during nonsolvent-induced phase separation. *Macromolecules*10.1021/acs.macromol.3c02394 (2024).

[47] Lu, X. et al. Ultrasound-driven piezoelectric stimulation of BMSCs on beta-PVDF to enhance exosome secretion for spinal cord injury repair. *Nano Energy* **151**, 111855. 10.1016/j.nanoen.2026.111855 (2026).

[48] Qi, H. et al. High-performance self-polarized PVDF film based on one-dimensional core-shell nanofiller and direct ink writing 3D printing. *Nano Energy* **150**, 111771. 10.1016/j.nanoen.2026.111771 (2026).

[49] Tanaka, H. Universality of viscoelastic phase separation in dynamically asymmetric fluid mixtures. *Phys. Rev. Lett.* **76**, 787–790. 10.1103/PhysRevLett.76.787 (1996). 10061550 10.1103/PhysRevLett.76.787

[50] Tanaka, H. Viscoelastic phase separation. *J. Phys. Condensed Matter* **12**, R207–R264. 10.1088/0953-8984/12/15/201 (2000).

[51] Tanaka, H. & Araki, T. Viscoelastic phase separation in soft matter: Numerical-simulation study on its physical mechanism. *Chem. Eng. Sci.* **61**, 2108–2141. 10.1016/j.ces.2004.02.025 (2006).

[52] Yoshimoto, K. & Taniguchi, T. Viscoelastic phase separation model for ternary polymer solutions. *J. Chem. Phys.* **154**, 104903. 10.1063/5.0039208 (2021). 33722036 10.1063/5.0039208

[53] Müller, M. & Abetz, V. Nonequilibrium processes in polymer membrane formation: Theory and experiment. *Chem. Rev.* **121**, 14189–14231. 10.1021/acs.chemrev.1c00029 (2021). 34032399 10.1021/acs.chemrev.1c00029

[54] Tanaka, H. Viscoelastic phase separation in soft matter and foods. *Faraday Discussions.* **158**, 371–406. 10.1039/C2FD20028G (2012) (**(Discussion 493–522)**). 23234176 10.1039/c2fd20028g

[55] Bottino, A., Capannelli, G., Munari, S. & Turturro, A. Solubility parameters of poly(vinylidene fluoride). *J. Polym. Sci. Part B Polym. Phys.* **26**, 785–794. 10.1002/polb.1988.090260405 (1988).

[56] Mezger, T.G. The Rheology Handbook: 4th Edition 4th ed. ISBN: 9783866308428 (Vincentz Network, Hannover, 2014).

[57] Blundell, D. J. & Keller, A. Nature of self-seeding polyethylene crystal nuclei. *J. Macromol. Sci. Part B* **2**, 301–336. 10.1080/00222346808212454 (1968).

[58] Guenet, J.-M. *Thermoreversible Gelation of Polymers and Biopolymers* (Academic Press, 1992) (**(ISBN: 0123053803)**).

[59] Onuki, A. Nonequilibrium phase transitions in extreme conditions: Effects of shear flow and heat flow. *J. Phys. Condensed Matter* **10**, 11473–11490. 10.1088/0953-8984/10/49/034 (1998).

[60] Hansen solubility parameters: A user’s handbook 2. ed. (ed Hansen, C.M.) ISBN: 9780849372483 (CRC, Boca Raton, Fla., 2007).

