# Supplementary material for: Formation of PVDF membranes with distinct pore morphologies interpreted through the framework of viscoelastic phase separation
Source: Sci Rep. 2026 May 9;16:14694. doi: 10.1038/s41598-026-50635-7 (PMC13157489; doi:10.1038/s41598-026-50635-7)
Supplement: Supplementary file 1 — Supplementary Information. [file 41598_2026_50635_MOESM1_ESM.zip › Supplementary Information Paper 2.docx]

Supplementary Information: Mechanism of PVDF membrane formation by VIPS revisited

## The effect of polymer dissolution temperature and solution viscoelasticity

Sven Johann Bohr1,2, Bruno Domnic1, Clemens Alexowsky2, Stéphan Barbe1, and Mathias Ulbricht2,*

1Faculty of Applied Natural Sciences, Cologne University of Applied Sciences, 51379 Leverkusen, Germany

2Department of Technical Chemistry II, University of Duisburg-Essen, 45141 Essen, Germany

*Corresponding author: [mathias.ulbricht@uni-essen.de](mailto:mathias.ulbricht@uni-essen.de)

09.09.2025

# S1 Supplementary information

## S1.1 Determination of minimum dissolution time *t_d,min_* and maximum processing time *t*_p,max_

DMSO dissolves PVDF only at elevated temperatures of approximately 60°C [[1].](#_bookmark1) Af- ter cooling to room temperature, PVDF/DMSO solutions become visibly turbid after approximately 30 minutes [[2].](#_bookmark2) Our research revealed that the properties of solutions of PVDF in DMSO are highly sensitive not only to the dissolution temperature *T_d_* but also to the dissolution time *t_d_*. To ensure the accuracy of analyses conducted on PVDF/DMSO solutions, it is imperative that all procedures are carried out at the selected *T_d_*. To mitigate this strict requirement, we assessed the stability of a ho- mogenous solution after its removal from the heat source to quantify the time period in which the solution remains stable enough for representative analysis.

50


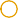

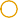

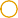


2.5

45

2.0

40

Temperature in °C

1.5

Extinction / 1

tcooling + tp,max

35

1.0

td,min

tcooling

30

0.5

25

0.0

20

0 50 100 150 200

Time in min

UV−Vis Extinction Temperature

Figure S1: Determination of the minimum dissolution time *t_d,min_* and the maximum processing time *t*_p,max_

The dissolution process of a 100-mL batch of 15 wt% was investigated. PVDF in DMSO, heated to 50 °C, was monitored using an inline UV-Vis probe and an inline temperature probe. The objective of the experiment was to identify two critical time

periods: first, the time required to form a clear, homogenous solution. Second, the time frame within which analyses must be conducted prior to the onset of turbidity, once a sample is removed from the heat source. As illustrated in Figure [S1,](#_bookmark0) the temperature and the extinction at a wavelength of *λ* = 265 nm are shown as a function of time. Upon attaining the target temperature, the extinction reached a minimum. The mean of the 25 lowest extinction values is equated to a clear solution. The minimum dissolution time, defined as the time when the extinction falls within a 3*σ*-range of the mean extinction value, is denoted as *t_d,min_*. Conversely, the maximum processing time, defined as the time when the extinction exceeds the 3*σ*-threshold, is denoted as *t*_p,max_. This approach effectively eliminates 99.73% of measurement noise that might be misinterpreted as indications of turbidity. Assuming these criteria, the solution is deemed clear after *t_d,min_* = 34 minutes. The solution was removed from the heater at *t_cooling_* = 76 minutes and after *t_cooling_* + *t_p,max_* = 95 minutes signs of turbidity appeared; thus, *t_p,max_* = 19 minutes. It is noteworthy that both parameters exhibit a correlation with batch size. Furthermore, a direct correlation between *t_d,min_* and polymer concentration is observed, while an inverse correlation is noted between *t_p,max_* and polymer concentration.

As initially stated, the sensitivity of PVDF/DMSO solutions to changes in *T_d_* or

*t_d_*, as well as their instability at room temperature, necessitates careful considera- tion during the development and implementation of analytical methodologies. These methodologies must be meticulously designed to preserve the delicate properties of the solutions without introducing substantial alterations. This requirement was met by adhering to the specific time intervals previously determined. The solution prepa- ration was conducted in accordance with method A (40 mL batch), with *t_d,min_* set to 34 minutes. The reproducibility of all subsequent analyses was ensured by setting *t*_p,max_ to 9 minutes. To the best of our knowledge, no other research on the dynamic behavior of solutions of semicrystalline polymers has been published. Consequently, a direct comparison of our results is not currently feasible.

## S1.2 The minimum dissolution temperature

100

10%

|  |  | |  |  |  | |  |  |  |  |  |
| --- | --- | --- | --- | --- | --- | --- | --- | --- | --- | --- | --- |
|  |  |  |  |  |  |  |  |  |  |  |  |
|  |  |  |  |  |  |  |  |  |  |  |  |
|  |  |  |  |  |  |  |  |  |  |  |  |
|  |  |  |  | T |  |  |  |  |  |  |  |
|  |  |  |  |  |  |  |  |  |  |  |  |
|  |  |  |  |  |  |  |  |  |  |  |  |
|  |  |  |  |  |  |  |  |  |  |  |  |
|  |  |  |  |  |  |  |  |  |  |  |  |
|  |  | |  |  |  | |  |  |  |  |  |

75

Transmission in %

50

d,min

25

90%

0

30 40 50 60

Temperature (°C)


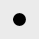
Measured data

Model

Td,min

T_d,min_ Range

Figure S2: The effect of *T_d_* on the transmission of a solution of 3 wt.% PVDF in DMSO measured at different wavelengths.

100

10%

75

50

Td,min

Transmission in %

25

0

90%

30 40 50 60

Temperature (°C)


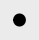
Measured data

Model

Td,min

T_d,min_ Range

Figure S3: The effect of *T_d_* on the transmission of a solution of 9 wt.% PVDF in DMSO measured at different wavelengths.

100

10%

75

50

Td,min

Transmission in %

25

0

90%

30 40 50 60

Temperature (°C)


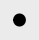
Measured data

Model

Td,min

T_d,min_ Range

Figure S4: The effect of *T_d_* on the transmission of a solution of 12 wt.% PVDF in DMSO measured at different wavelengths.

100

10%

75

50

Td,min

Transmission in %

25

0

90%

30 40 50 60

Temperature (°C)


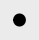
Measured data

Model

Td,min

T_d,min_ Range

Figure S5: The effect of *T_d_* on the transmission of a solution of 18 wt.% PVDF in DMSO measured at different wavelengths.

## S1.3 The critical dissolution temperature

80

10%

60

40

F_α_ in %

20

Td,crit

0

90%

30 40 50 60

Temperature (°C)


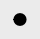
Measured data

Model

Td,crit

Td,crit Range

Figure S6: The effect of *T_d_* on the fraction of *α*-polymorph in the membrane prepared from 3 wt.% PVDF in DMSO.

80

10%

60

40

F_α_ in %

20

Td,crit

0

90%

30 40 50 60

Temperature (°C)


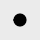
Measured data

Model

Td,crit

Td,crit Range

Figure S7: The effect of *T_d_* on the fraction of *α*-polymorph in the membrane prepared from 9 wt.% PVDF in DMSO.

80

|  |  |  |  | |  |  |  |  |  |  |
| --- | --- | --- | --- | --- | --- | --- | --- | --- | --- | --- |
|  |  |  |  | 10% |  |  |  |  |  |  |
|  |  |  |  |  |  |  |  |  |  |  |
|  |  |  |  |  |  |  |  |  |  |  |
|  |  |  |  |  |  |  |  |  |  |  |
|  |  |  |  |  |  |  |  |  |  |  |
|  |  |  |  |  |  | T |  |  |  |  |
|  |  |  |  |  |  | d,crit |  |  |  |  |
|  |  |  |  |  |  |  | 90% |  |  |  |
|  |  |  |  | |  |  |  |  |  |  |

60

40

F_α_ in %

20

0

30 40 50 60

Temperature (°C)


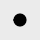
Measured data

Model

Td,crit

Td,crit Range

Figure S8: The effect of *T_d_* on the fraction of *α*-polymorph in the membrane prepared from 12 wt.% PVDF in DMSO.

80

10%

60

40

F_α_ in %

20

Td,crit

0

90%

30 40 50 60

Temperature (°C)


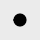
Measured data

Model

Td,crit

Td,crit Range

Figure S9: The effect of *T_d_* on the fraction of *α*-polymorph in the membrane prepared from 18 wt.% PVDF in DMSO.

## S1.4 Rheological properties alongside minimum and critical dissolution temperature

1e+04

Td,crit

Td,min

Wi = 1

1e+02

Weissenberg number

1e+00

1e-02

30 35 40 45 50

Temperature (°C)


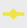
Weissenberg number

Td,min Range

Tcrit Range

Figure S10: The minimum dissolution temperature at different wavelengths, the criti- cal dissolution temperature, their respective temperature ranges, and the rheological data of a solution of 3 wt.% PVDF in DMSO.

1e+04

Td,crit

Td,min

Wi = 1

1e+02

Weissenberg number

1e+00

1e-02

35 40 45 50 55

Temperature (°C)


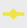
Weissenberg number

Td,min Range

Tcrit Range

Figure S11: The minimum dissolution temperature at different wavelengths, the criti- cal dissolution temperature, their respective temperature ranges, and the rheological data of a solution of 9 wt.% PVDF in DMSO.

1e+04

= 1

Wi

min

Td,

Td,crit

1e+02

Weissenberg number

1e+00

1e-02

35 40 45 50 55 60 65

Temperature (°C)


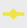
Weissenberg number

Td,min Range

Tcrit Range

Figure S12: The minimum dissolution temperature at different wavelengths, the criti- cal dissolution temperature, their respective temperature ranges, and the rheological data of a solution of 12 wt.% PVDF in DMSO.

1e+04

1e+02

Weissenberg number

1e+00

1e-02

40 45 50 55 60 65 70

|  |  |  |  |  |  |  |  |  |  |  |  |  |  |
| --- | --- | --- | --- | --- | --- | --- | --- | --- | --- | --- | --- | --- | --- |
|  |  | 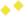 |  |  |  | Td,crit |  |  |  |  |  |  |  |
|  |  |  |  |  | Td,min |  |  |  |  |  |  |  |  |
|  |  |  |  |  |  |  |  |  |  |  |  |  |  |
|  |  |  |  |  |  |  |  |  |  |  |  | Wi = 1 |  |
|  |  |  |  |  |  |  |  |  |  |  |  |  |  |
|  |  |  |  |  |  |  |  |  |  |  |  |  |  |
|  |  |  |  |  |  |  |  |  |  |  |  |  |  |

Temperature (°C)


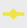
Weissenberg number

Td,min Range

Tcrit Range

Figure S13: The minimum dissolution temperature at different wavelengths, the criti- cal dissolution temperature, their respective temperature ranges, and the rheological data of a solution of 18 wt.% PVDF in DMSO.

# References

1. A. Bottino et al. “Solubility parameters of poly(vinylidene fluoride).” In: *Journal of Polymer Science Part B: Polymer Physics* 26.4 (1988), pp. 785–794. issn: 0887-6266. doi: [10.1002/polb.1988.090260405](https://doi.org/10.1002/polb.1988.090260405).
2. Clemens Alexowsky. “Herstellung von porösen Polyvinylidenfluorid-Membranen mit maßgeschneiderten Eigenschaften durch schnelle und skalierbare dampfind- uzierte Phasentrennung.” Dissertation. Essen: Universität Duisburg-Essen, 2019. url: <https://doi.org/10.17185/duepublico/70271>.
