# Supplementary material for: Formation of PVDF membranes with distinct pore morphologies interpreted through the framework of viscoelastic phase separation
Source: Sci Rep. 2026 May 9;16:14694. doi: 10.1038/s41598-026-50635-7 (PMC13157489; doi:10.1038/s41598-026-50635-7)
Supplement: Supplementary file 1 — Supplementary Information. [file 41598_2026_50635_MOESM1_ESM.zip › Supplementary Information Paper 2.pdf]

Supplementary Information: Formation of PVDF membranes with distinct pore morphologies interpreted through the framework of viscoelastic phase separation

Sven Johann Bohr[1,2], Bruno Richard Domnick[3], Clemens Alexowsky[4], Stéphan Barbe[1], and Mathias Ulbricht[2,*]

[1]Faculty of Applied Natural Sciences, Cologne University of Applied Sciences, 51379 Leverkusen, Germany
[2]Department of Technical Chemistry II, University of Duisburg-Essen, 45141 Essen, Germany
[3]Faculty of Process Engineering, Energy and Mechanical Systems, Cologne University of Applied Sciences, 50679 Cologne, Germany
[4]Evonik Operations GmbH, 45127 Essen, Germany
[*]Corresponding author: mathias.ulbricht@uni-essen.de

07.04.2026

S1 Supplementary information

S1.1 Determination of minimum dissolution time $t_{d,min}$ and maximum processing time $t_{\mathrm{p,max}}$

DMSO dissolves PVDF only at elevated temperatures of approximately 60°C [1]. After cooling to room temperature, PVDF/DMSO solutions become visibly turbid after approximately 30 minutes [2]. Our research revealed that the properties of solutions of PVDF in DMSO are highly sensitive not only to the dissolution temperature T_d but also to the dissolution time t_d. To ensure the accuracy of analyses conducted on PVDF/DMSO solutions, it is imperative that all procedures are carried out at the selected T_d. To mitigate this strict requirement, we assessed the stability of a homogenous solution after its removal from the heat source to quantify the time period in which the solution remains stable enough for representative analysis.

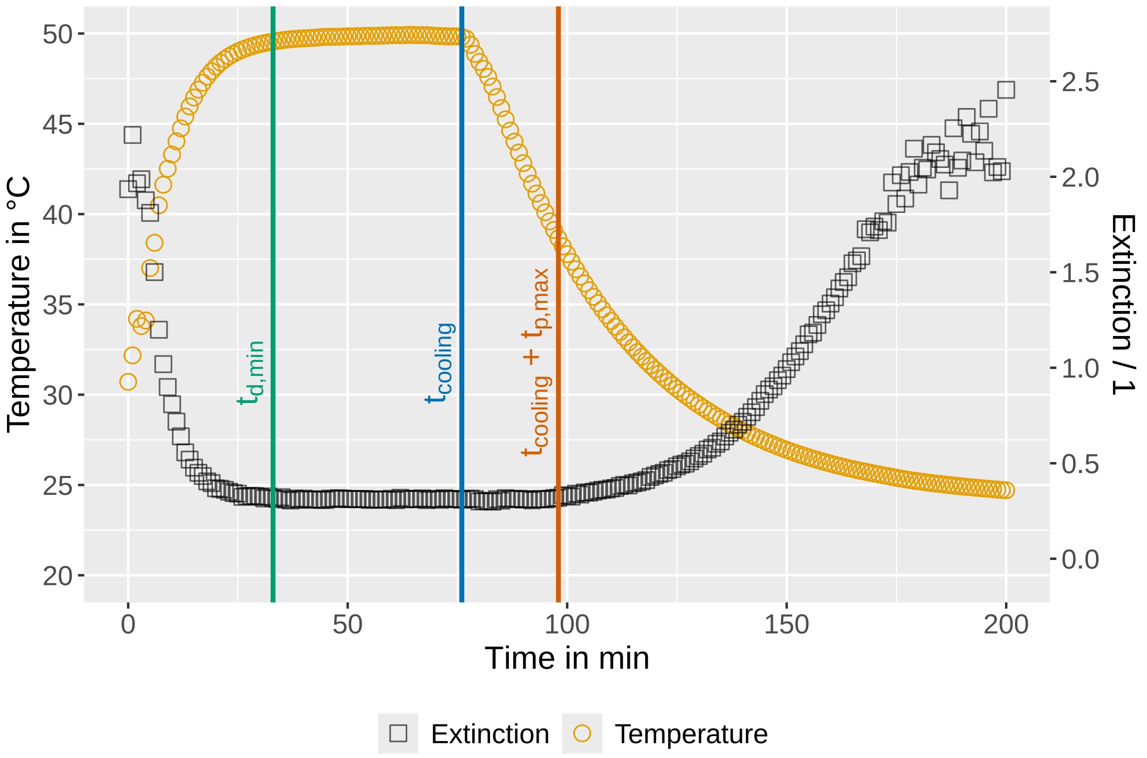

Figure S1: Determination of the minimum dissolution time $t_{d,min}$ and the maximum processing time $t_{\mathrm{p,max}}$ based on the optical extinction at 380 nm.

The dissolution process of a 100-mL batch of 15 wt% was investigated. PVDF in DMSO, heated to 50 °C, was monitored using an inline UV-Vis probe and an inline temperature probe. The objective of the experiment was to identify two critical time

periods: first, the time required to form a clear, homogenous solution. Second, the time frame within which analyses must be conducted prior to the onset of turbidity, once a sample is removed from the heat source. As illustrated in Figure S1, the temperature and the extinction at a wavelength of $\lambda = 265$ nm are shown as a function of time. Upon attaining the target temperature, the extinction reached a minimum. The mean of the 25 lowest extinction values is equated to a clear solution. The minimum dissolution time, defined as the time when the extinction falls within a 3σ-range of the mean extinction value, is denoted as $t_{d,min}$. Conversely, the maximum processing time, defined as the time when the extinction exceeds the 3σ-threshold, is denoted as $t_{\mathrm{p,max}}$. This approach effectively eliminates 99.73% of measurement noise that might be misinterpreted as indications of turbidity. Assuming these criteria, the solution is deemed clear after $t_{d,min} = 34$ minutes. The solution was removed from the heater at $t_{cooling} = 76$ minutes and after $t_{cooling} + t_{p,max} = 95$ minutes signs of turbidity appeared; thus, $t_{p,max} = 19$ minutes. It is noteworthy that both parameters exhibit a correlation with batch size. Furthermore, a direct correlation between $t_{d,min}$ and polymer concentration is observed, while an inverse correlation is noted between $t_{p,max}$ and polymer concentration.

As initially stated, the sensitivity of PVDF/DMSO solutions to changes in T_d or t_d, as well as their instability at room temperature, necessitates careful consideration during the development and implementation of analytical methodologies. These methodologies must be meticulously designed to preserve the delicate properties of the solutions without introducing substantial alterations. This requirement was met by adhering to the specific time intervals previously determined. The solution preparation was conducted in accordance with method A (40 mL batch), with $t_{d,min}$ set to 34 minutes. The reproducibility of all subsequent analyses was ensured by setting $t_{\mathrm{p,max}}$ to 9 minutes. To the best of our knowledge, no other research on the dynamic behavior of solutions of semicrystalline polymers has been published. Consequently, a direct comparison of our results is not currently feasible.

S1.2 The minimum dissolution temperature

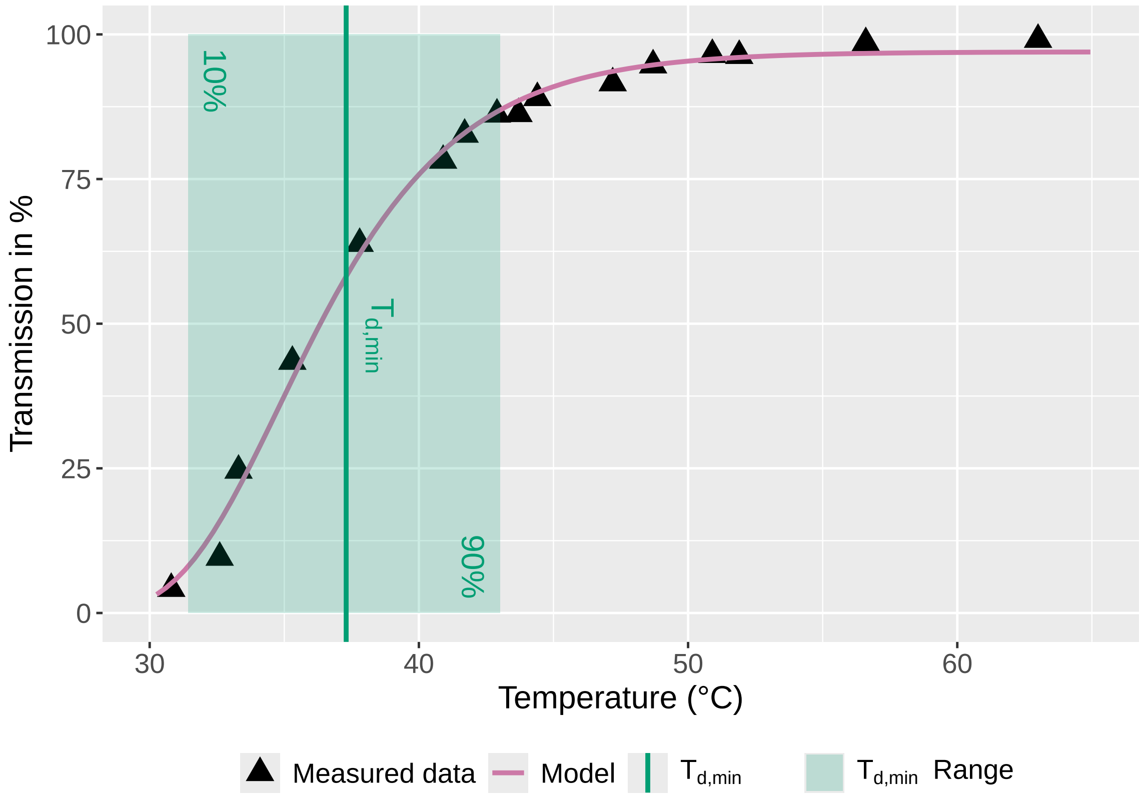

Figure S2: Effect of T_d on the optical transmission at 380 nm of a 3 wt.% PVDF/DMSO solution. The experimental data were fitted using a modified Gompertz function with a $pseudoR^2$ of 0.995. The T_d range corresponding to a 10–90 % increase in transmission is highlighted by a green rectangle. $T_{d,min}$ is defined as the temperature at which the transmission has increased by 60 %.

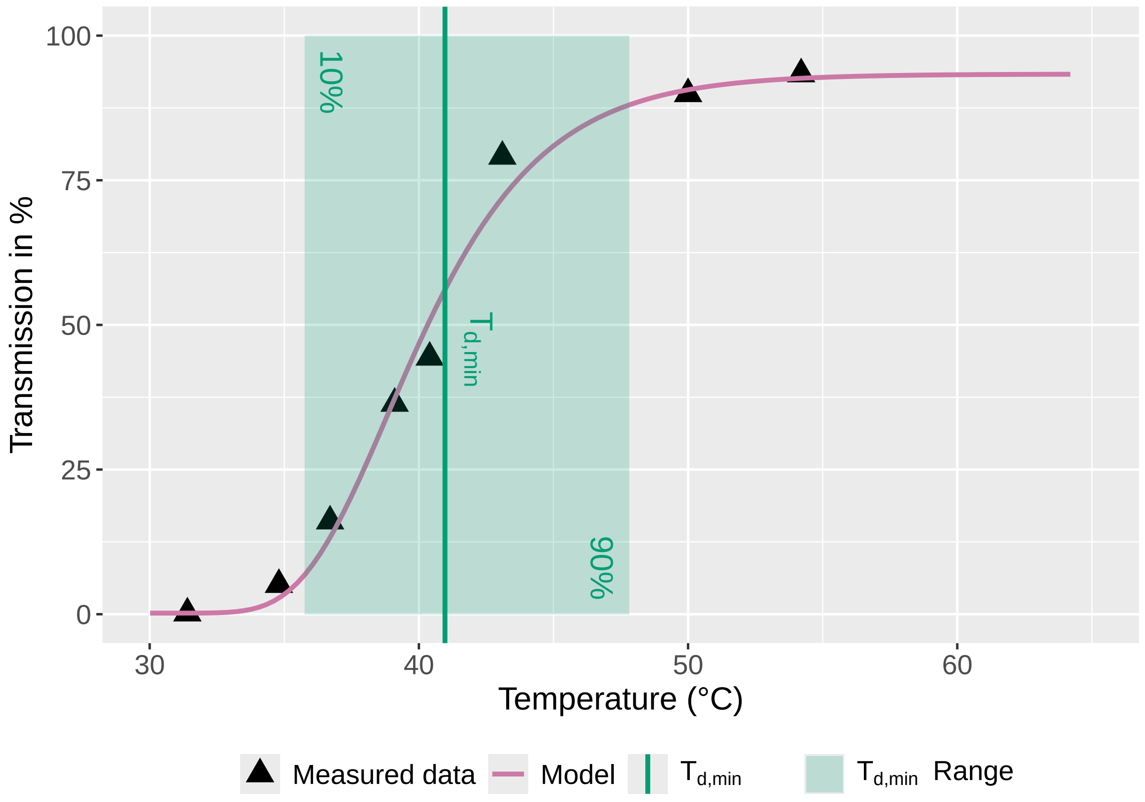

Figure S3: Effect of T_d on the optical transmission at 380 nm of a 9 wt.% PVDF/DMSO solution. The experimental data were fitted using a modified Gompertz function with a $pseudoR^2$ of 0.989. The T_d range corresponding to a 10–90 % increase in transmission is highlighted by a green rectangle. $T_{d,min}$ is defined as the temperature at which the transmission has increased by 60 %.

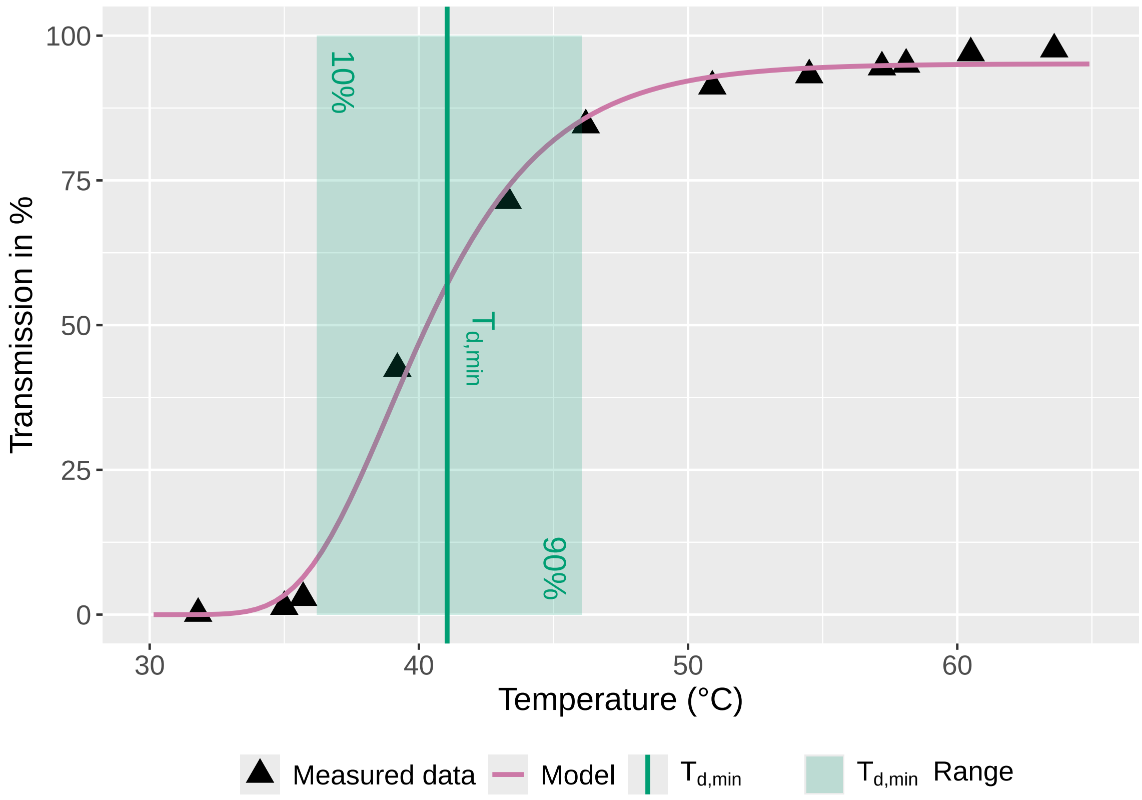

Figure S4: Effect of T_d on the optical transmission at 380 nm of a 12 wt.% PVDF/DMSO solution. The experimental data were fitted using a modified Gompertz function with a $pseudoR^2$ of 0.997. The T_d range corresponding to a 10–90 % increase in transmission is highlighted by a green rectangle. $T_{d,min}$ is defined as the temperature at which the transmission has increased by 60 %.

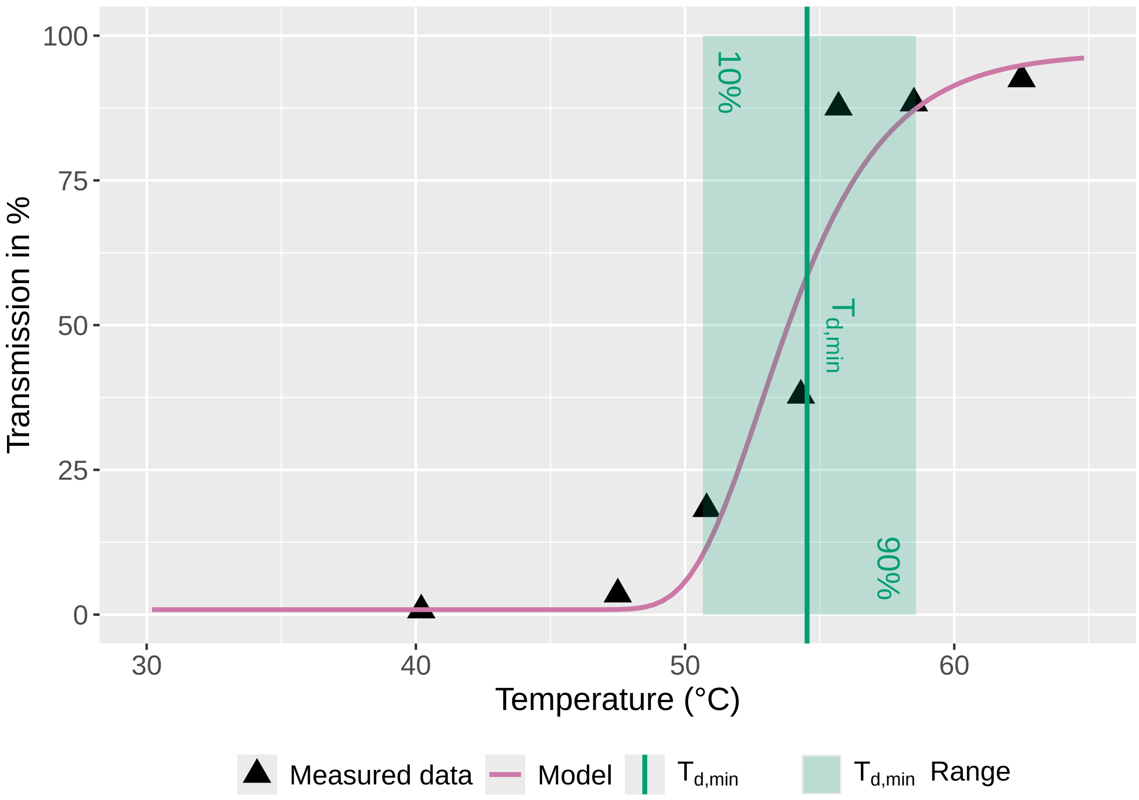

Figure S5: Effect of T_d on the optical transmission at 380 nm of a 18 wt.% PVDF/DMSO solution. The experimental data were fitted using a modified Gompertz function with a $pseudoR^2$ of 0.952. The T_d range corresponding to a 10–90 % increase in transmission is highlighted by a green rectangle. $T_{d,min}$ is defined as the temperature at which the transmission has increased by 60 %.

S1.3 The critical dissolution temperature

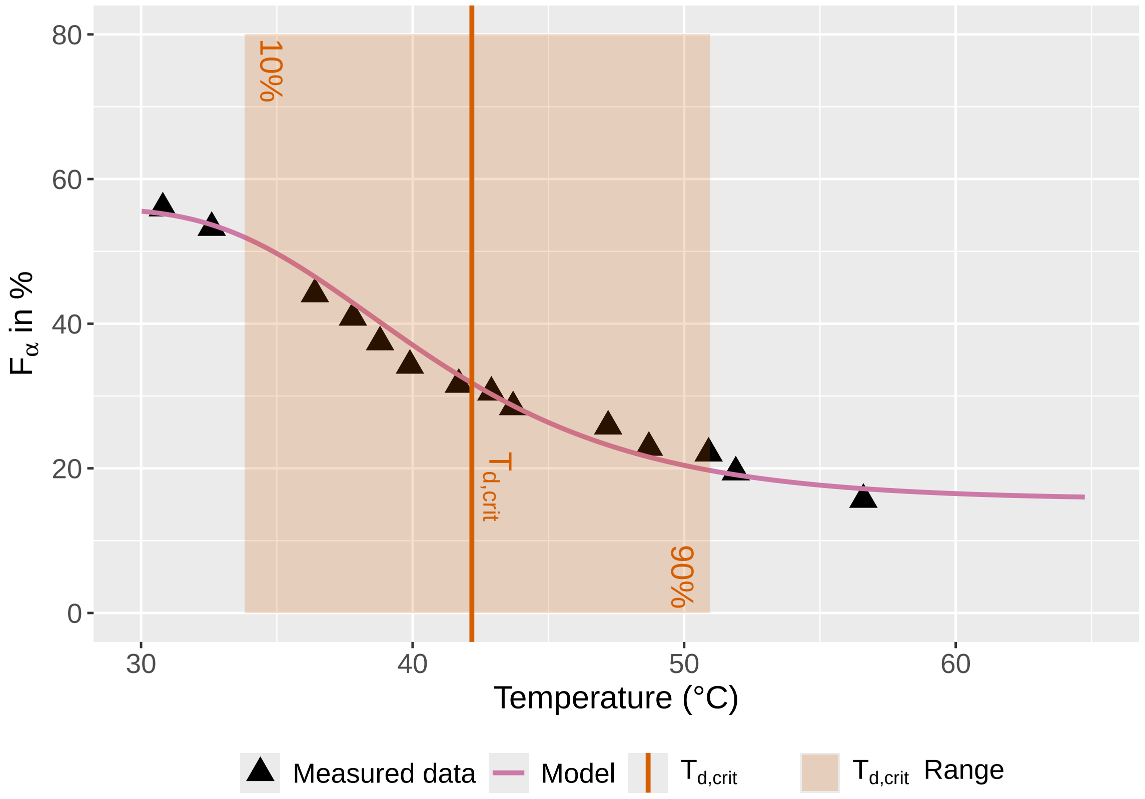

Figure S6: Effect of T_d on the fraction of α-polymorph in a membrane prepared from a 3 wt.% PVDF/DMSO solution. The experimental data were fitted using a modified Gompertz function with a $pseudoR^2$ of 0.976. The T_d range corresponding to a 10–90 % decrease in α-polymorph content is highlighted by a red rectangle. $T_{d,crit}$ is defined as the temperature at which the α-polymorph content has decreased by 60 %.

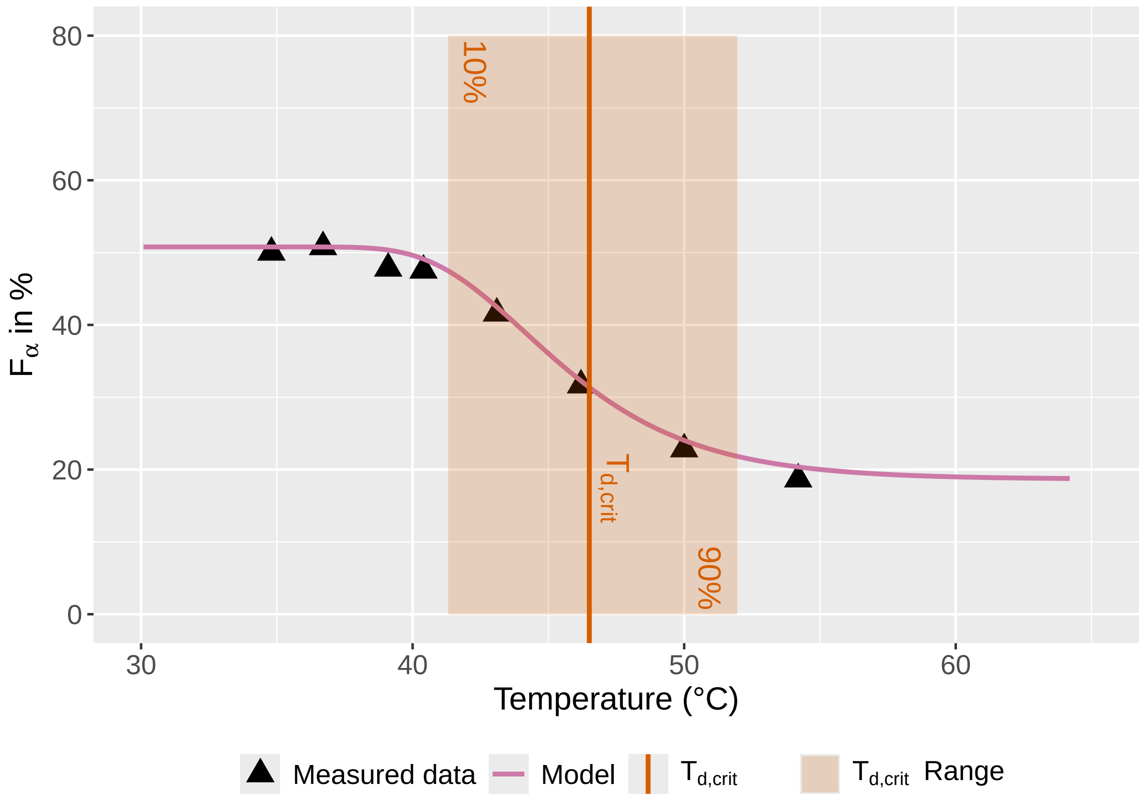

Figure S7: Effect of T_d on the fraction of α-polymorph in a membrane prepared from a 9 wt.% PVDF/DMSO solution. The experimental data were fitted using a modified Gompertz function with a $pseudoR^2$ of 0.987. The T_d range corresponding to a 10–90 % decrease in α-polymorph content is highlighted by a red rectangle. $T_{d,crit}$ is defined as the temperature at which the α-polymorph content has decreased by 60 %.

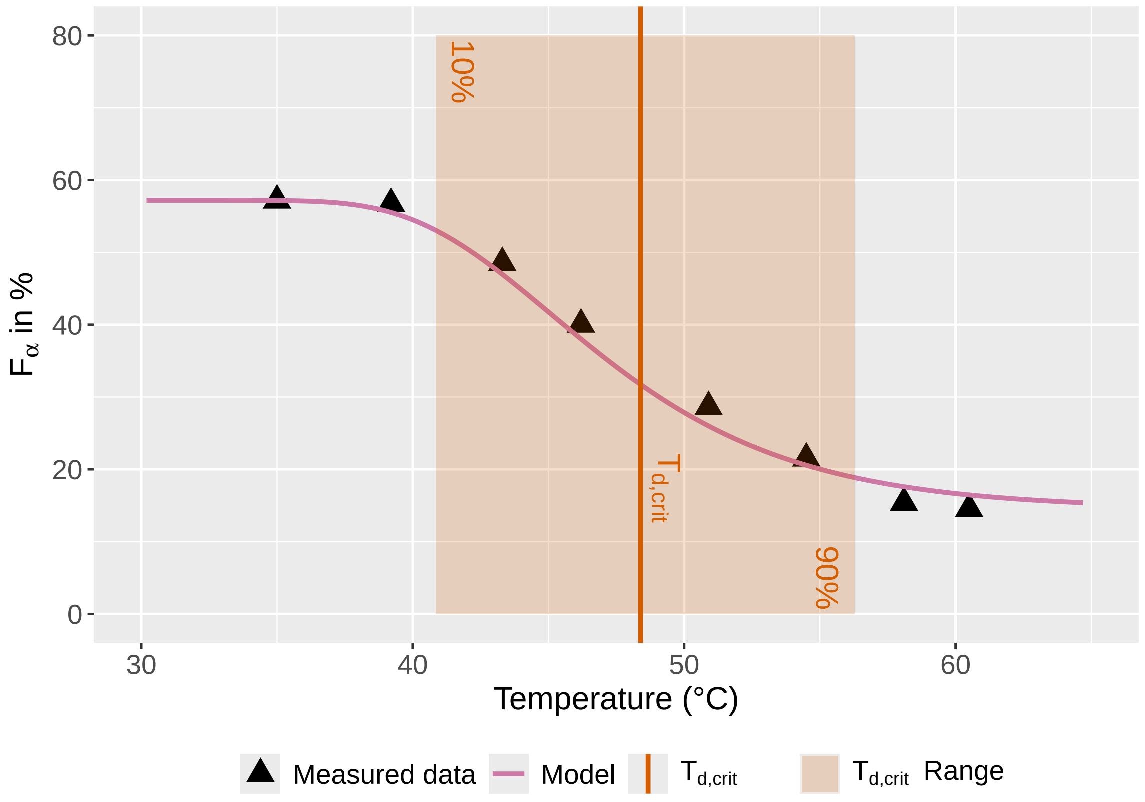

Figure S8: Effect of T_d on the fraction of α-polymorph in a membrane prepared from a 12 wt.% PVDF/DMSO solution. The experimental data were fitted using a modified Gompertz function with a $pseudoR^2$ of 0.989. The T_d range corresponding to a 10–90 % decrease in α-polymorph content is highlighted by a red rectangle. $T_{d,crit}$ is defined as the temperature at which the α-polymorph content has decreased by 60 %.

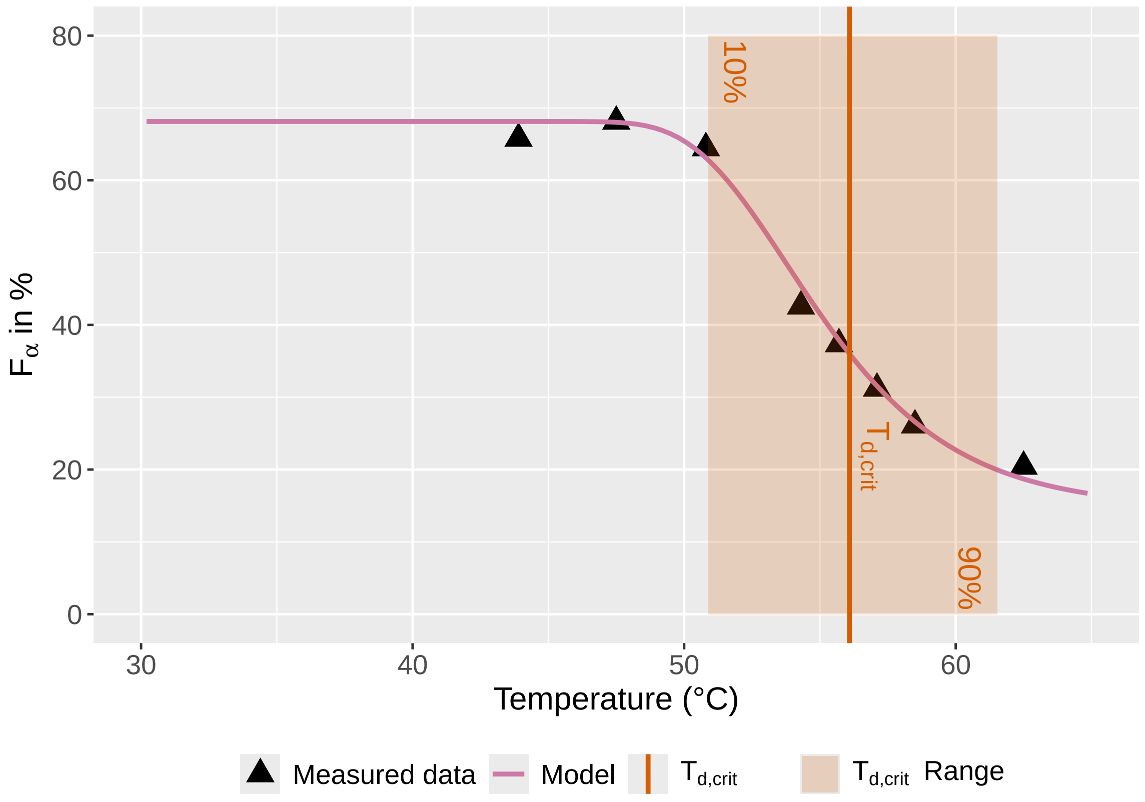

Figure S9: Effect of T_d on the fraction of α-polymorph in a membrane prepared from a 18 wt.% PVDF/DMSO solution. The experimental data were fitted using a modified Gompertz function. The T_d range corresponding to a 10–90 % decrease in α-polymorph content is highlighted by a red rectangle. $T_{d,crit}$ is defined as the temperature at which the α-polymorph content has decreased by 60 %.

S1.4 Rheological properties alongside minimum and critical dissolution temperature

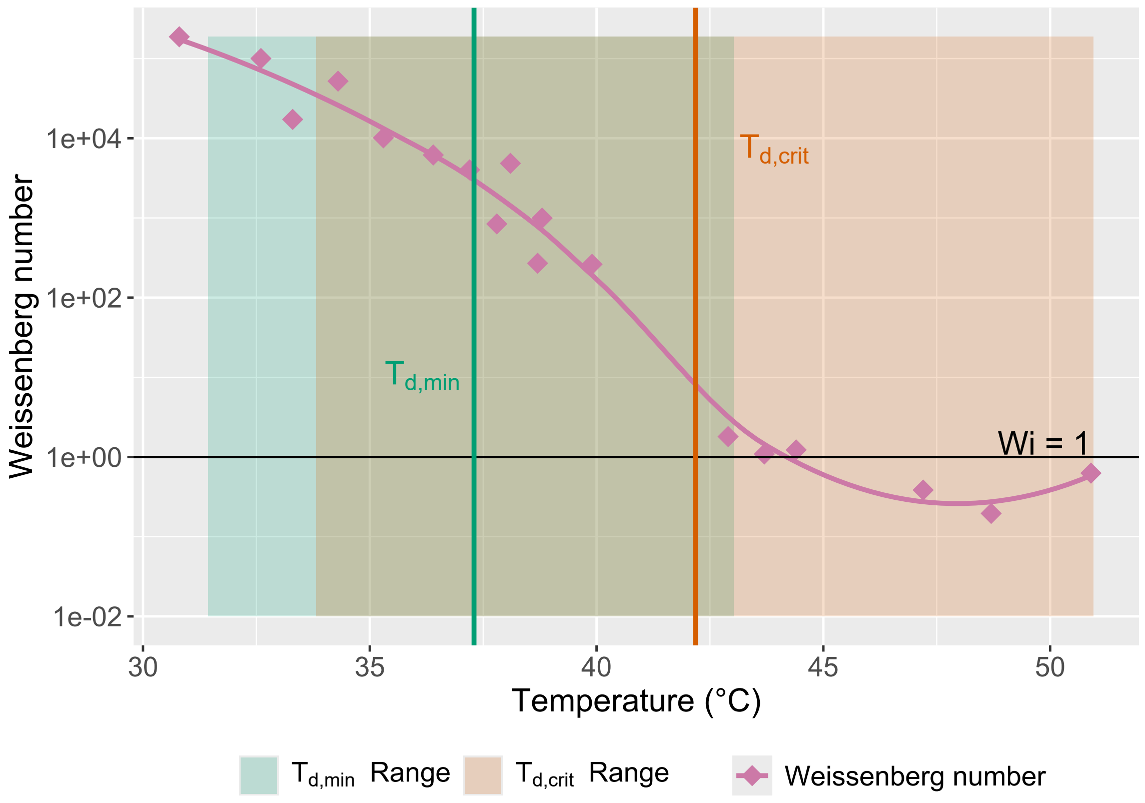

Figure S10: Weissenberg number as a function of T_d of a solution of 3 wt.% PVDF in DMSO. The minimum dissolution temperature $T_{d,min}$ and the critical dissolution temperature $T_{d,crit}$ are indicated by green and red vertical lines, respectively. The corresponding temperature ranges are highlighted by green and red rectangles.

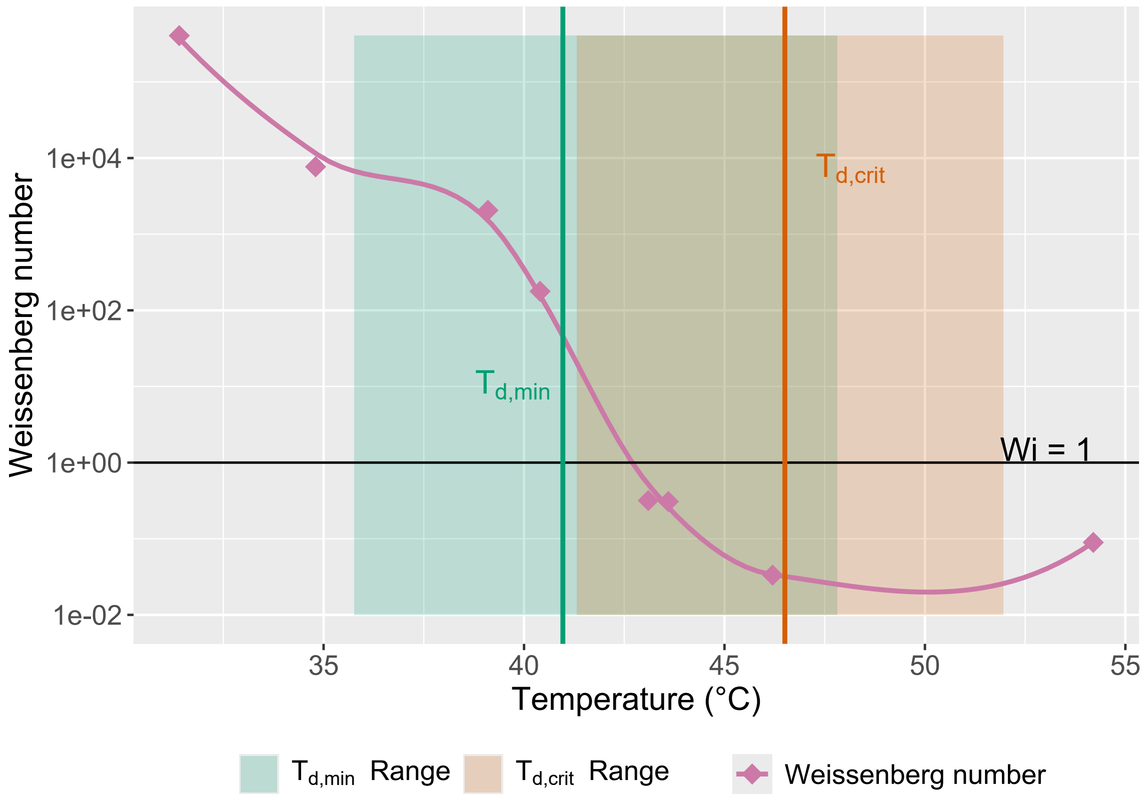

Figure S11: Weissenberg number as a function of T_d of a solution of 9 wt.% PVDF in DMSO. The minimum dissolution temperature $T_{d,min}$ and the critical dissolution temperature $T_{d,crit}$ are indicated by green and red vertical lines, respectively. The corresponding temperature ranges are highlighted by green and red rectangles.

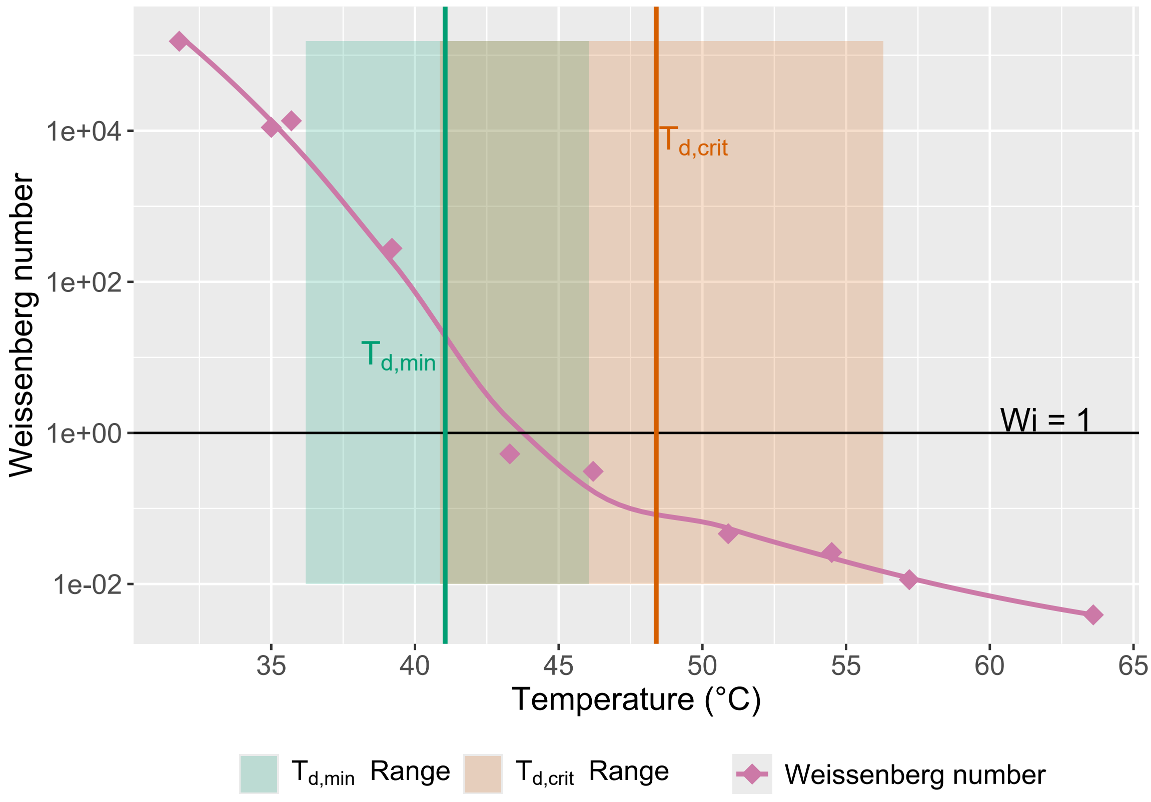

Figure S12: Weissenberg number as a function of T_d of a solution of 12 wt.% PVDF in DMSO. The minimum dissolution temperature $T_{d,min}$ and the critical dissolution temperature $T_{d,crit}$ are indicated by green and red vertical lines, respectively. The corresponding temperature ranges are highlighted by green and red rectangles.

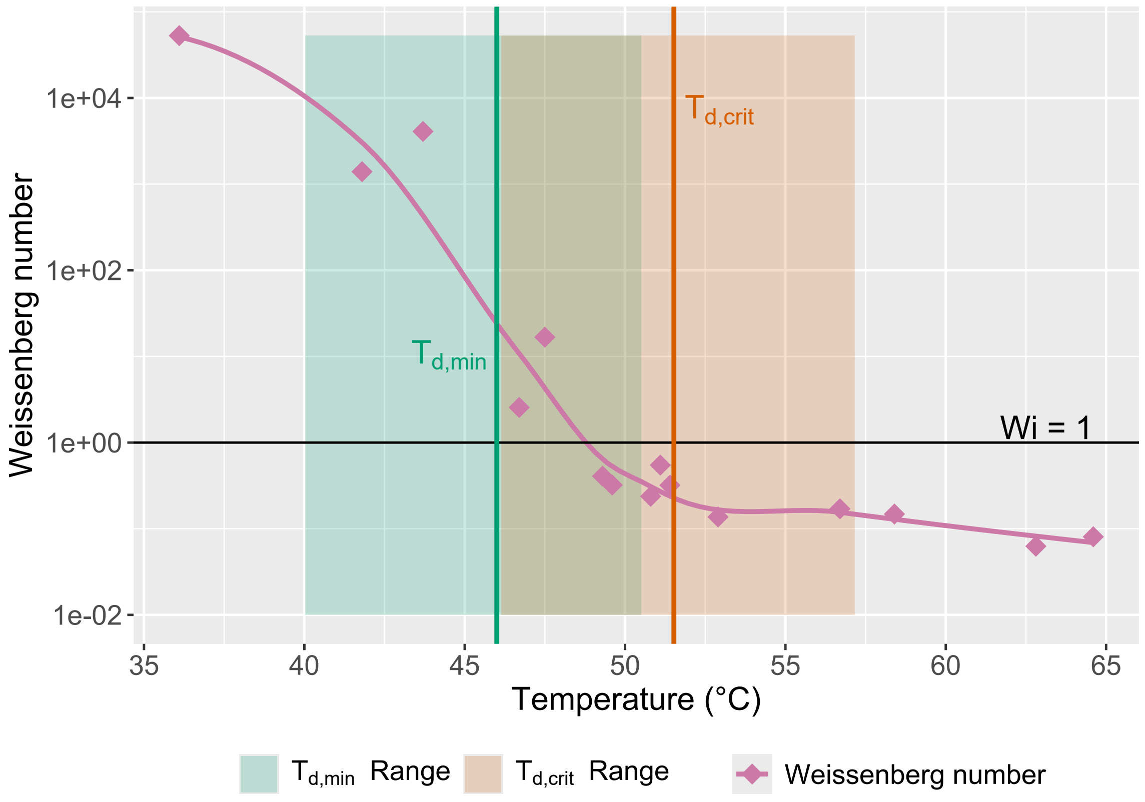

Figure S13: Weissenberg number as a function of T_d of a solution of 15 wt.% PVDF in DMSO. The minimum dissolution temperature $T_{d,min}$ and the critical dissolution temperature $T_{d,crit}$ are indicated by green and red vertical lines, respectively. The corresponding temperature ranges are highlighted by green and red rectangles.

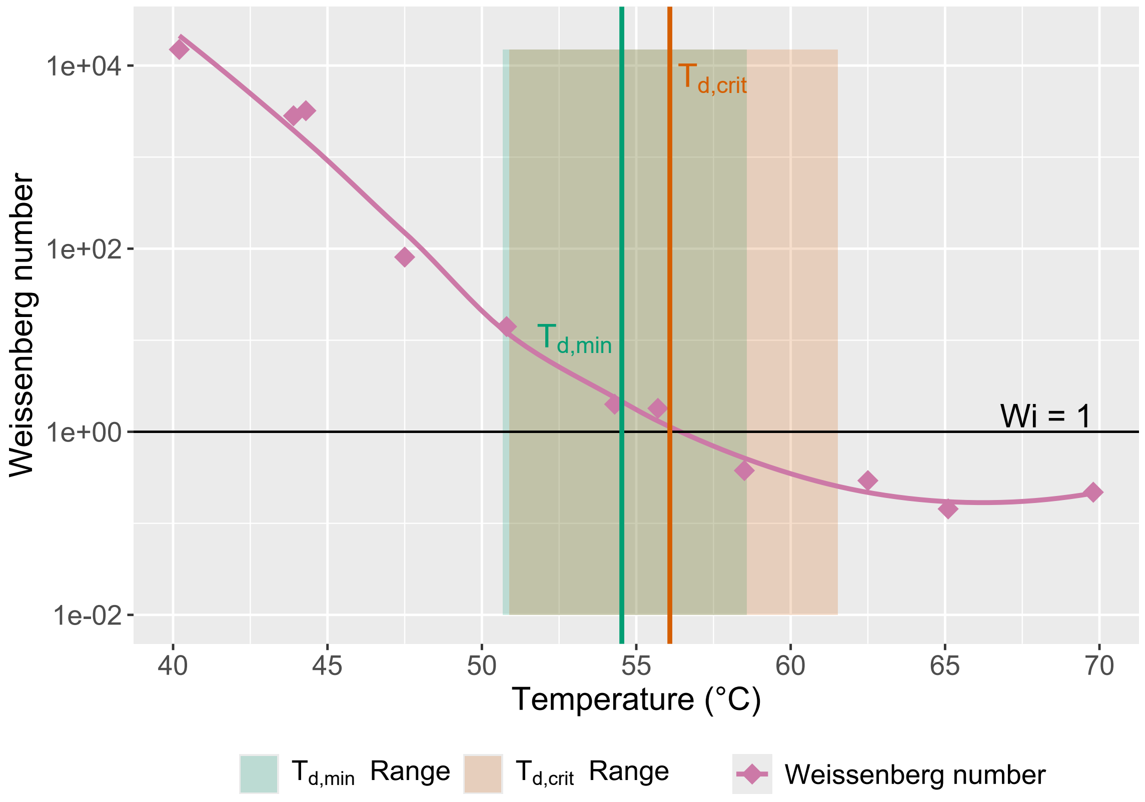

Figure S14: Weissenberg number as a function of T_d of a solution of 18 wt.% PVDF in DMSO. The minimum dissolution temperature $T_{d,min}$ and the critical dissolution temperature $T_{d,crit}$ are indicated by green and red vertical lines, respectively. The corresponding temperature ranges are highlighted by green and red rectangles.

References

1. Bottino, A., Capannelli, G., Munari, S. & Turturro, A. Solubility parameters of poly(vinylidene fluoride). *Journal of Polymer Science Part B: Polymer Physics* **26,** 785–794. ISSN: 0887-6266. doi:10.1002/polb.1988.090260405 (1988).
2. Alexowsky, C. *Herstellung von porösen Polyvinylidenfluorid-Membranen mit maßgeschneiderten Eigenschaften durch schnelle und skalierbare dampfinduzierte Phasentrennung* Dissertation (Universität Duisburg-Essen, Essen, 2019).
