# Supplementary figures and images for: Formation of PVDF membranes with distinct pore morphologies interpreted through the framework of viscoelastic phase separation
Source: Sci Rep. 2026 May 9;16:14694. doi: 10.1038/s41598-026-50635-7 (PMC13157489; doi:10.1038/s41598-026-50635-7)

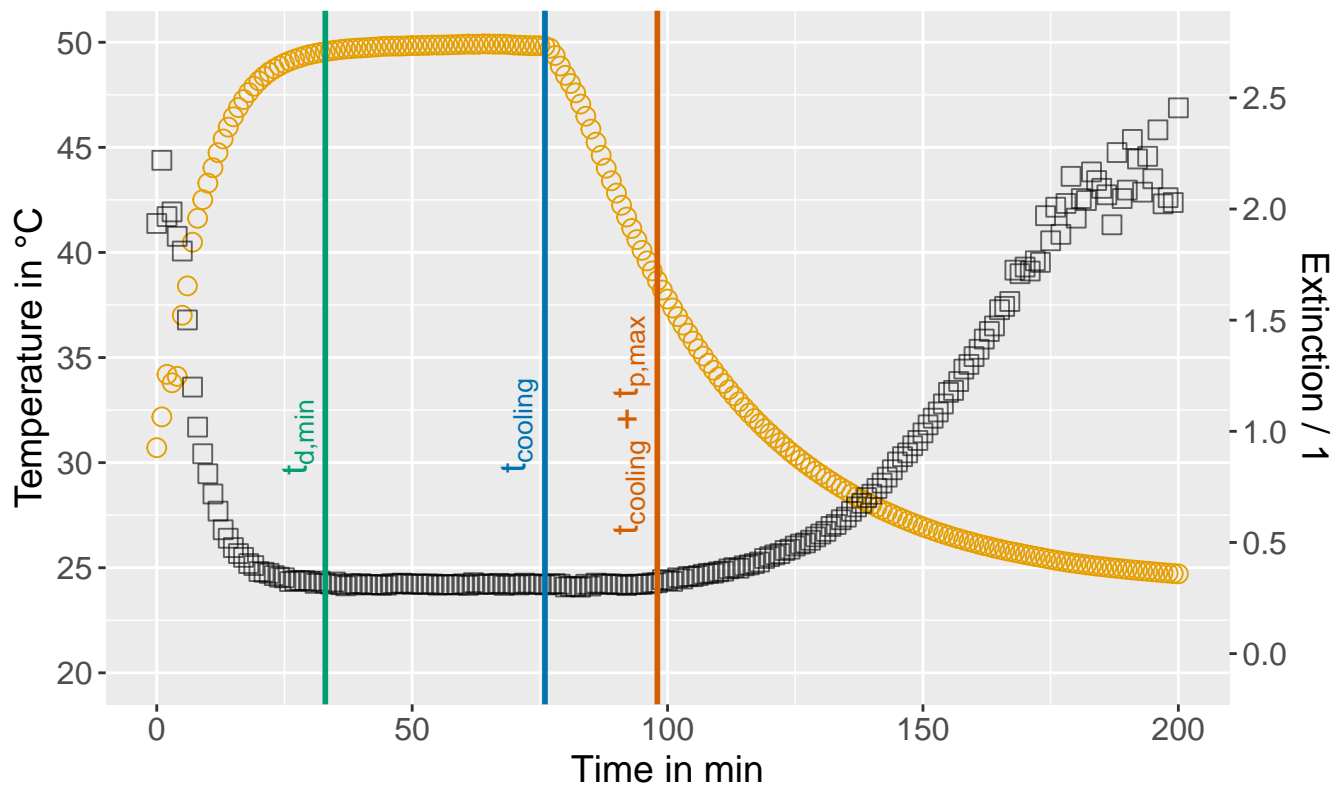

□ Extinction    ○ Temperature

Supplement: Supplementary file 1 — Supplementary Information. [file 41598_2026_50635_MOESM1_ESM.zip › fig_Determination_of_t_dmin_t_pmax.pdf]

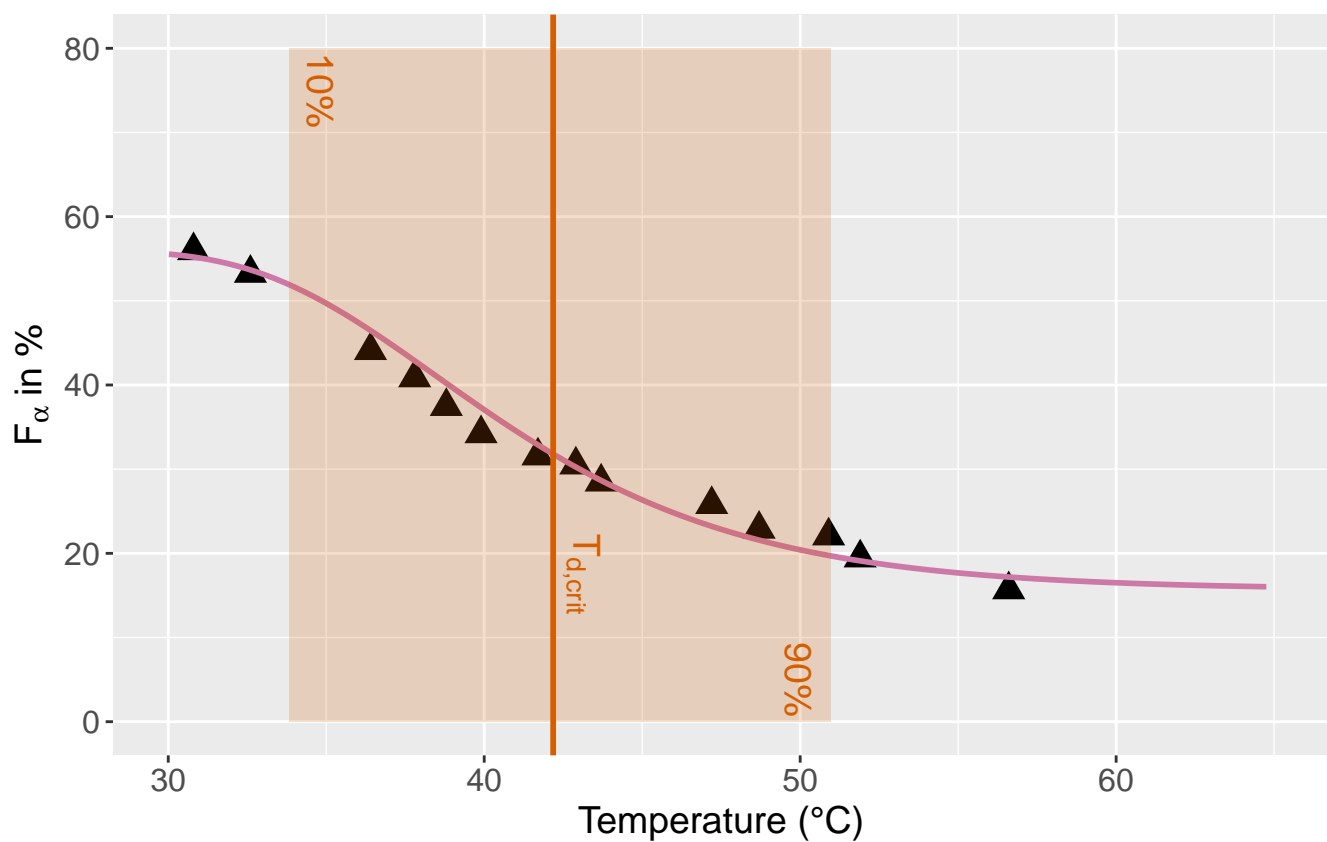

▲ Measured data    — Model    |  $T_{d,crit}$      $T_{d,crit}$  Range

Supplement: Supplementary file 1 — Supplementary Information. [file 41598_2026_50635_MOESM1_ESM.zip › fig_Effect_of_Td_on_alpha-polymorph_in_3_wt_PVDF_60.pdf]

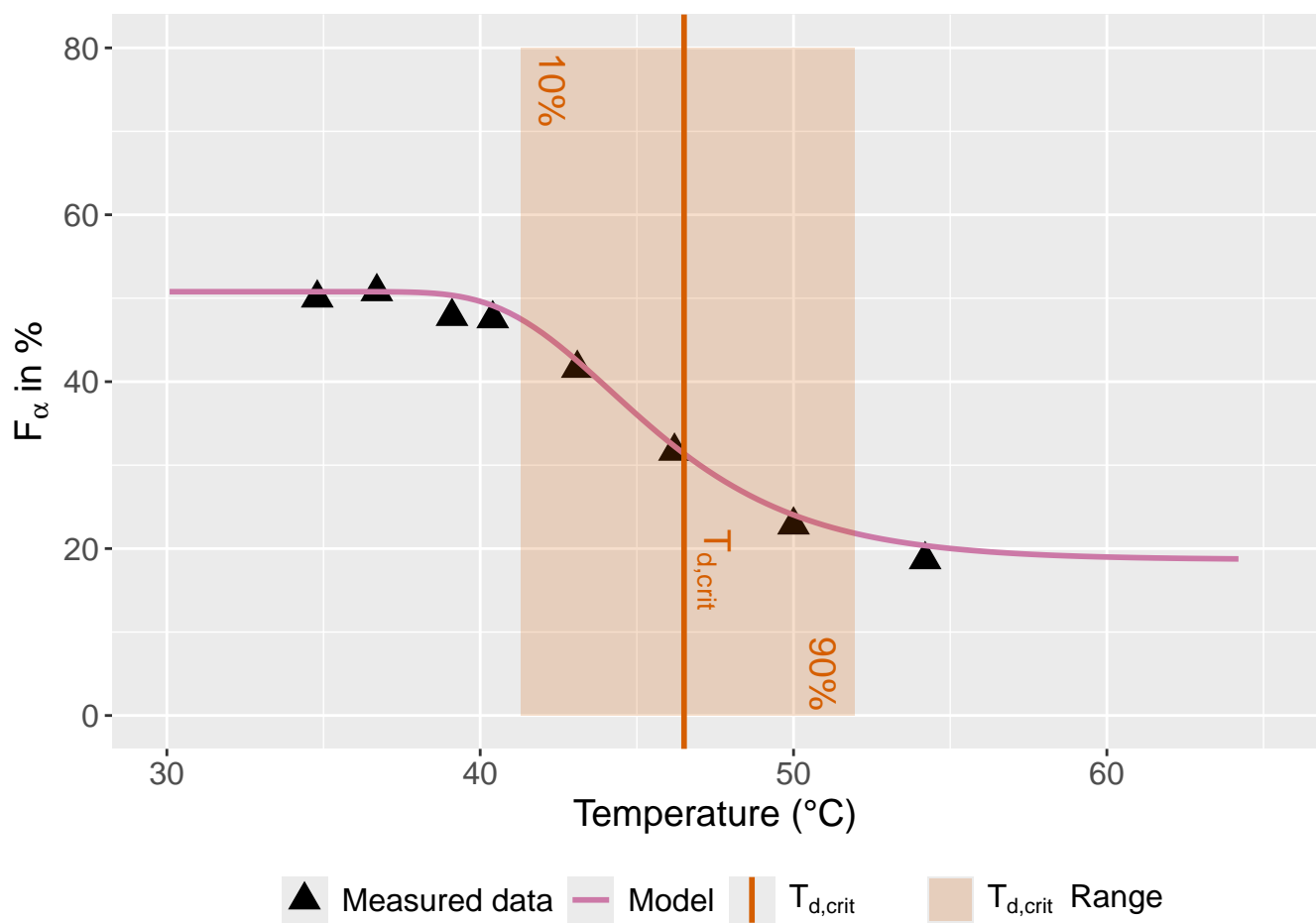

Supplement: Supplementary file 1 — Supplementary Information. [file 41598_2026_50635_MOESM1_ESM.zip › fig_Effect_of_Td_on_alpha-polymorph_in_9_wt_PVDF_60.pdf]

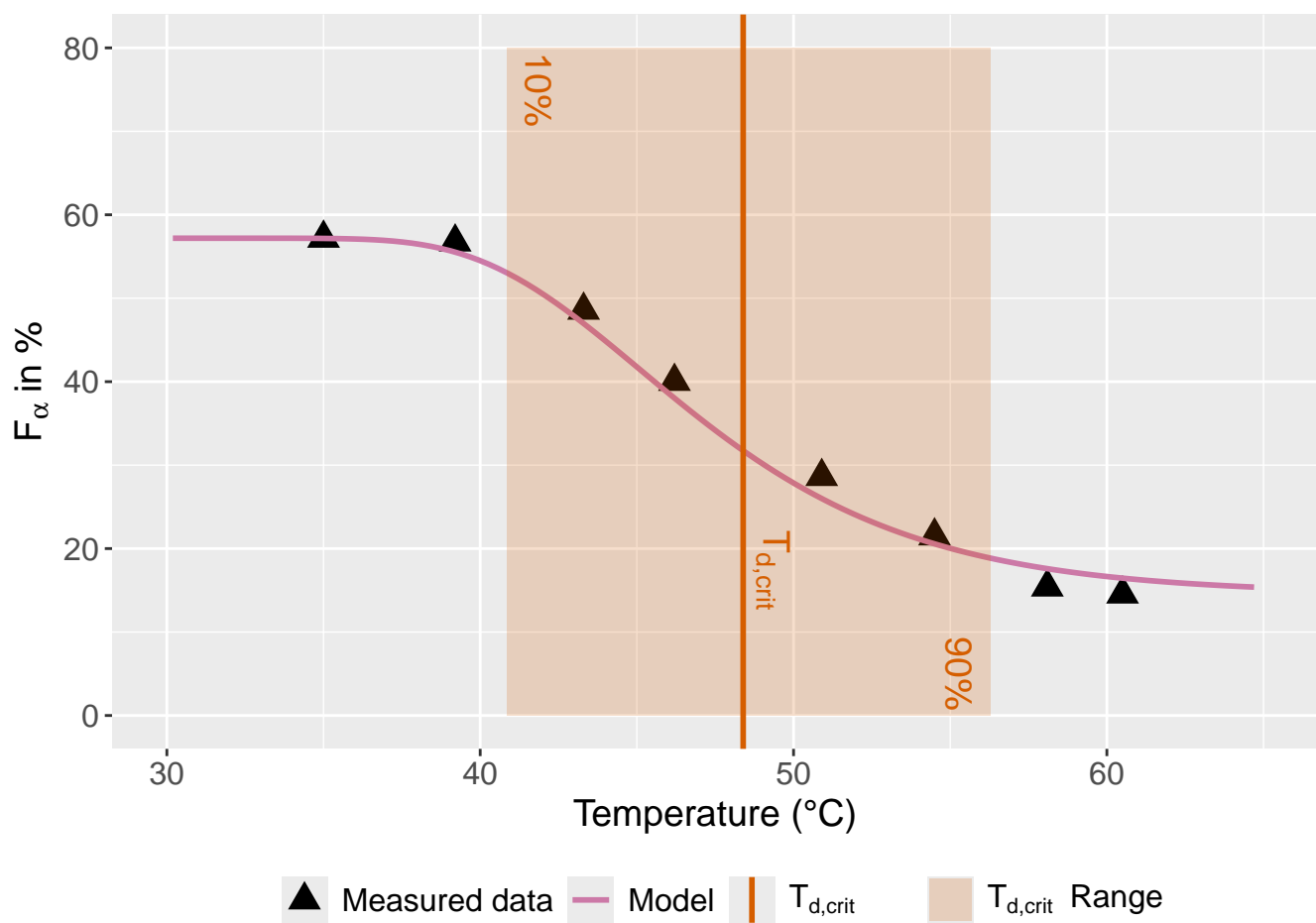

Supplement: Supplementary file 1 — Supplementary Information. [file 41598_2026_50635_MOESM1_ESM.zip › fig_Effect_of_Td_on_alpha-polymorph_in_12_wt_PVDF_60.pdf]

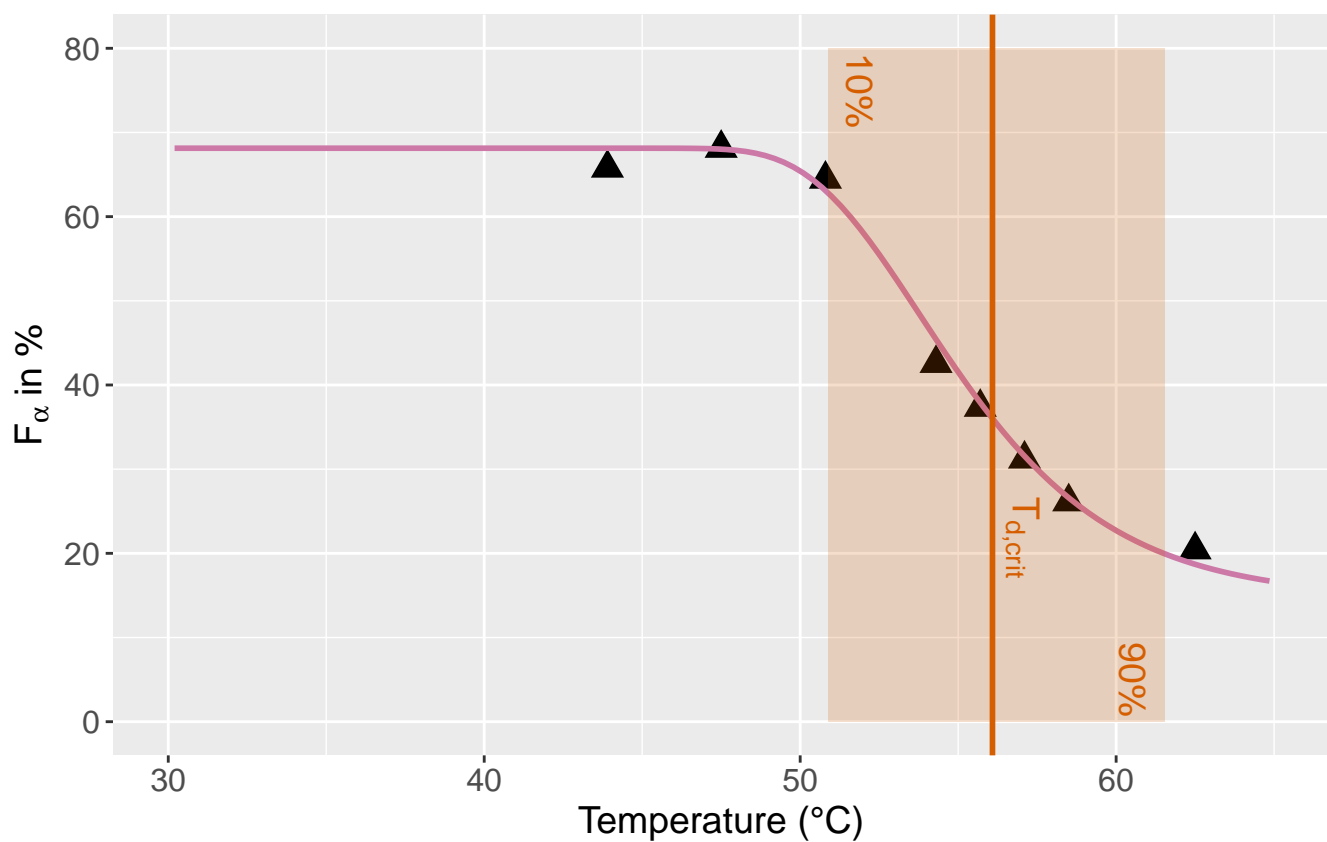

▲ Measured data    — Model    |  $T_{d,crit}$      $T_{d,crit}$  Range

Supplement: Supplementary file 1 — Supplementary Information. [file 41598_2026_50635_MOESM1_ESM.zip › fig_Effect_of_Td_on_alpha-polymorph_in_18_wt_PVDF_60.pdf]

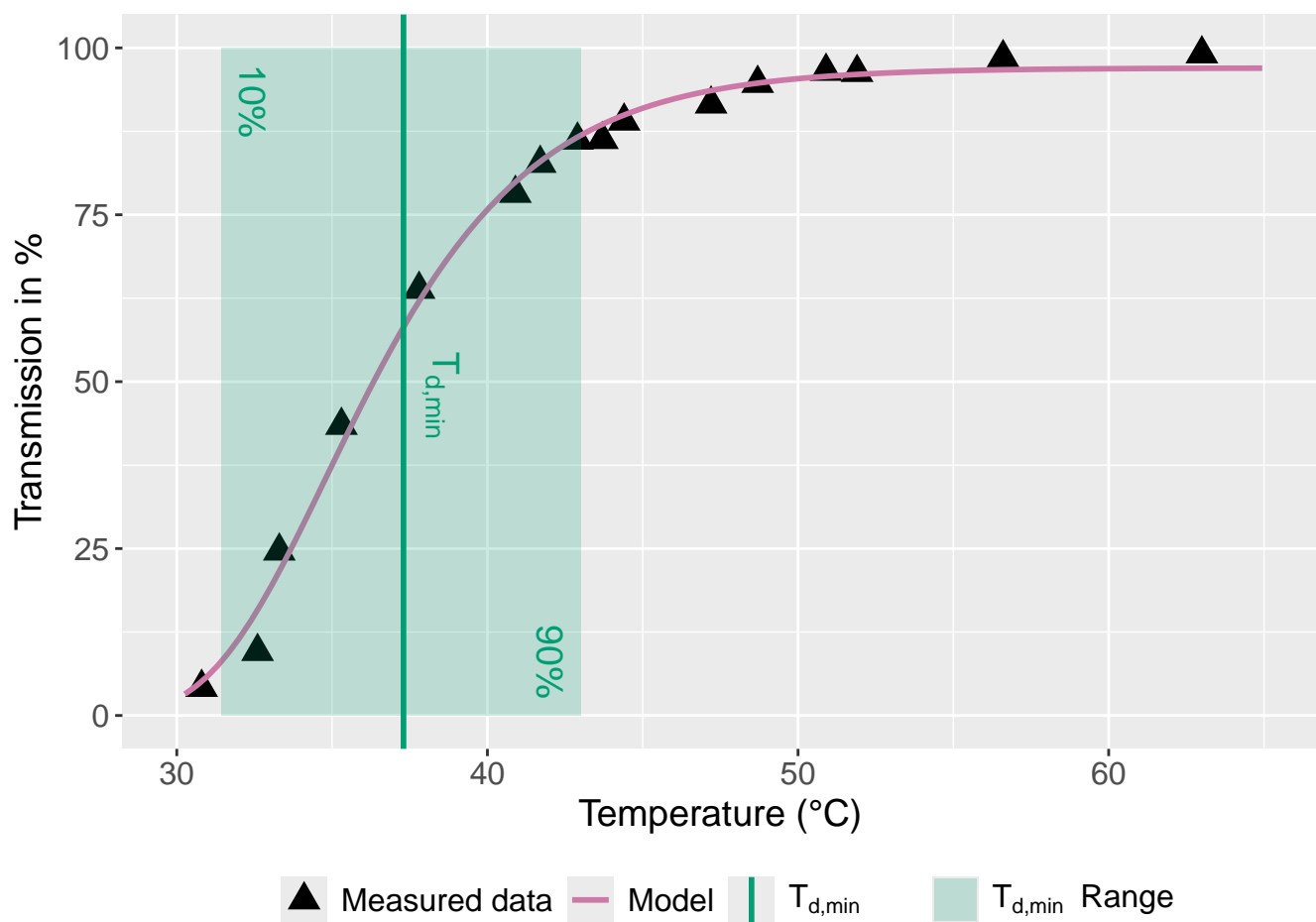

Supplement: Supplementary file 1 — Supplementary Information. [file 41598_2026_50635_MOESM1_ESM.zip › fig_Effect_of_Td_on_transmission_at_380_in_3_wt_PVDF_60.pdf]

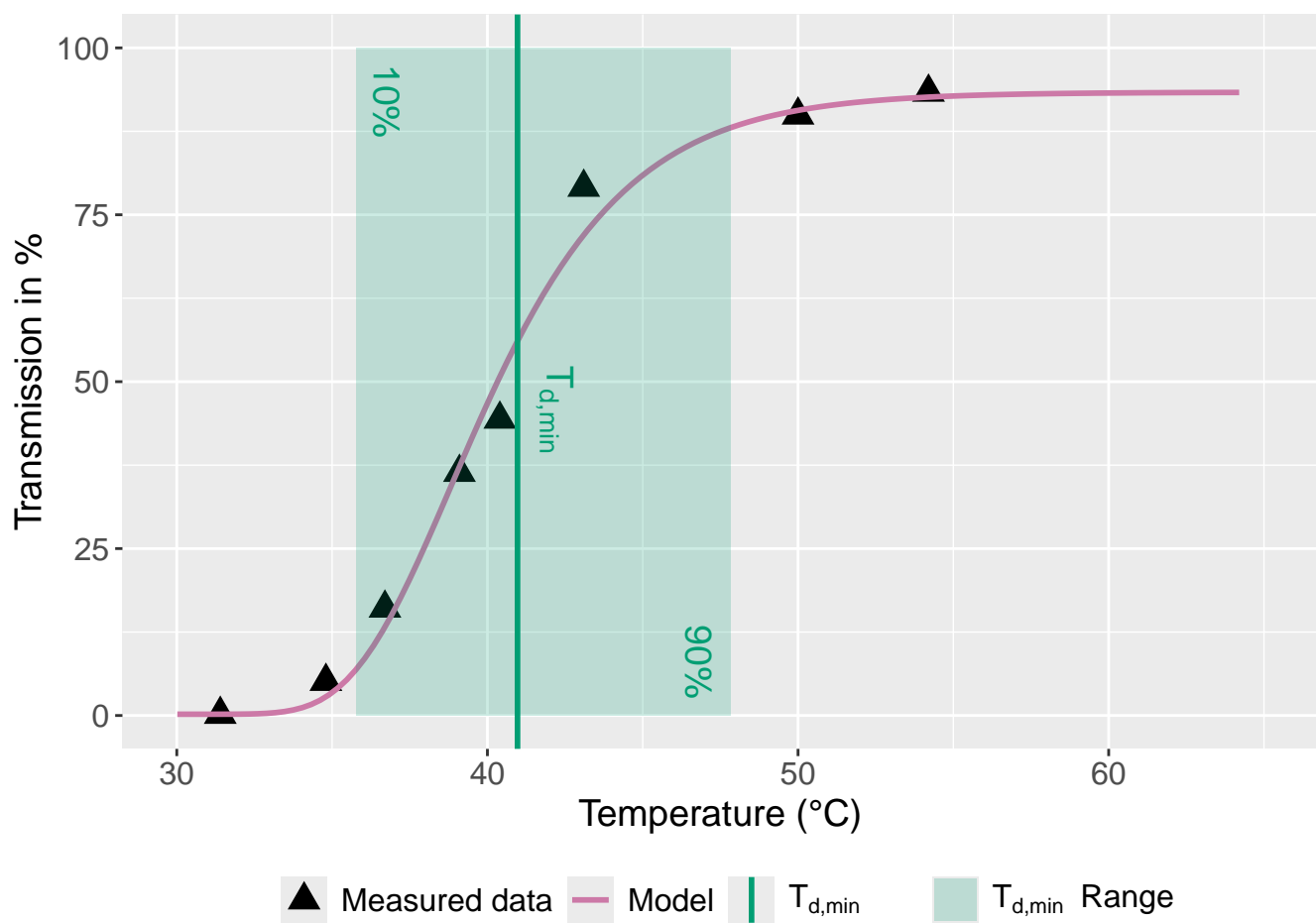

Supplement: Supplementary file 1 — Supplementary Information. [file 41598_2026_50635_MOESM1_ESM.zip › fig_Effect_of_Td_on_transmission_at_380_in_9_wt_PVDF_60.pdf]

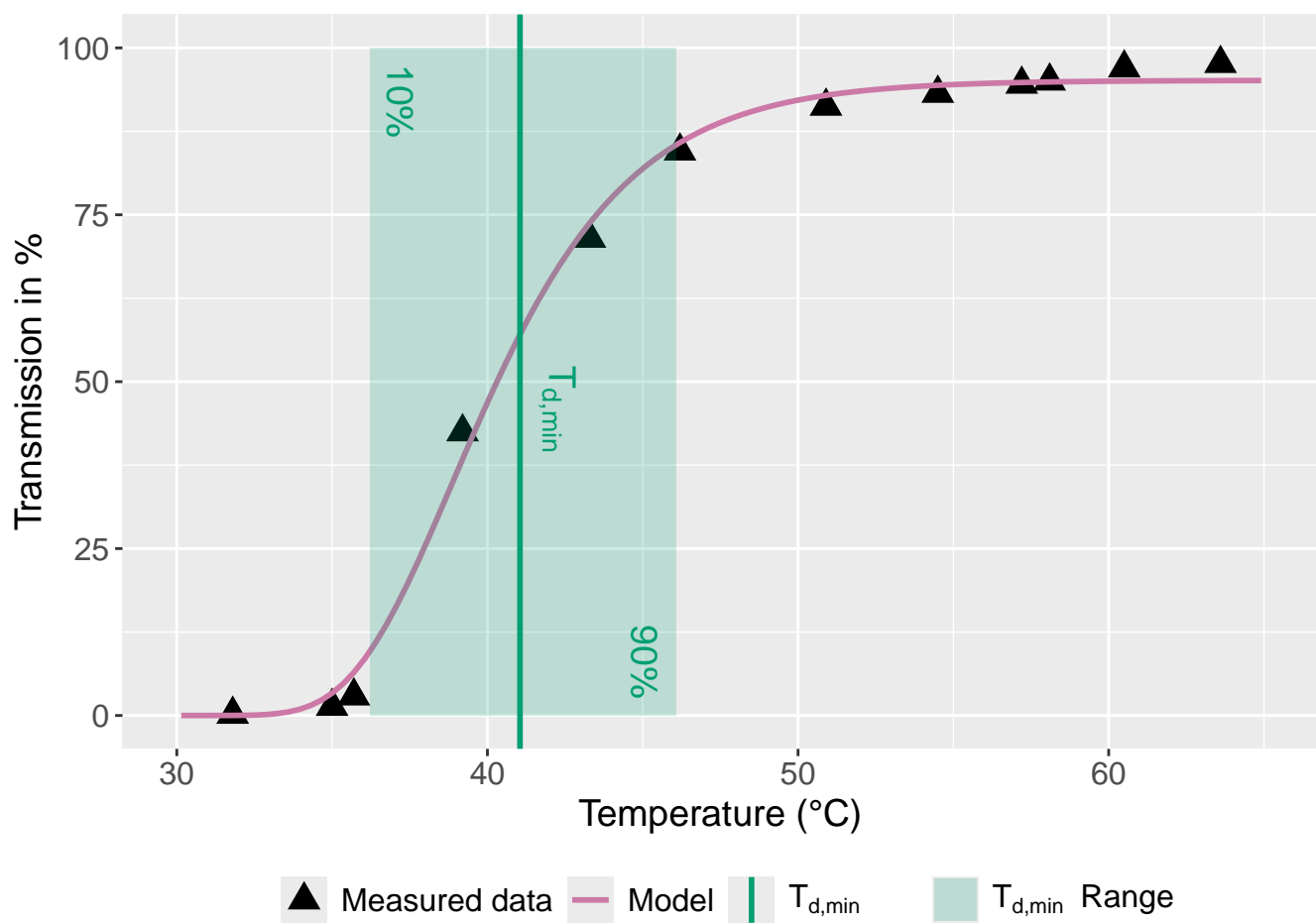

Supplement: Supplementary file 1 — Supplementary Information. [file 41598_2026_50635_MOESM1_ESM.zip › fig_Effect_of_Td_on_transmission_at_380_in_12_wt_PVDF_60.pdf]

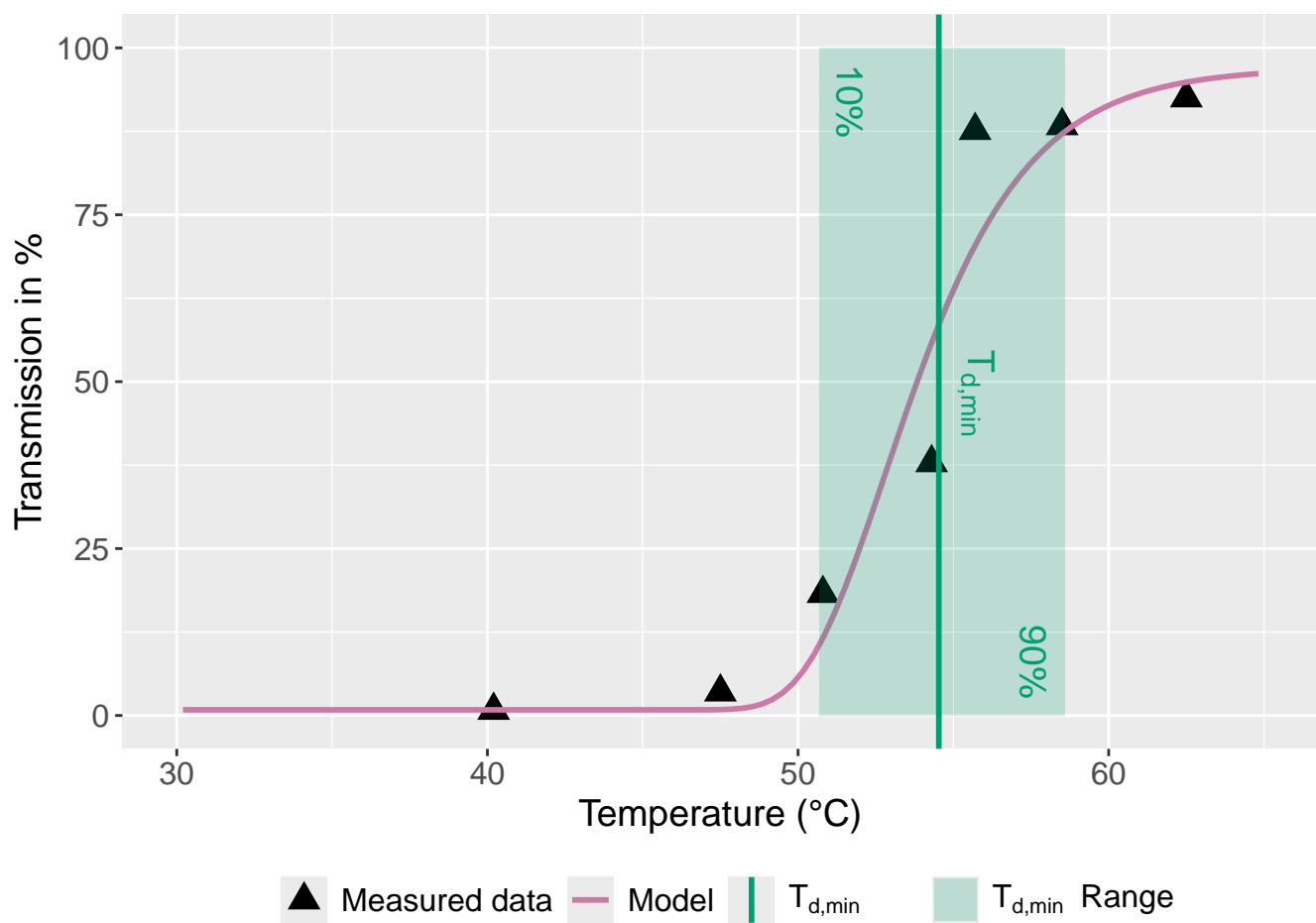

Supplement: Supplementary file 1 — Supplementary Information. [file 41598_2026_50635_MOESM1_ESM.zip › fig_Effect_of_Td_on_transmission_at_380_in_18_wt_PVDF_60.pdf]

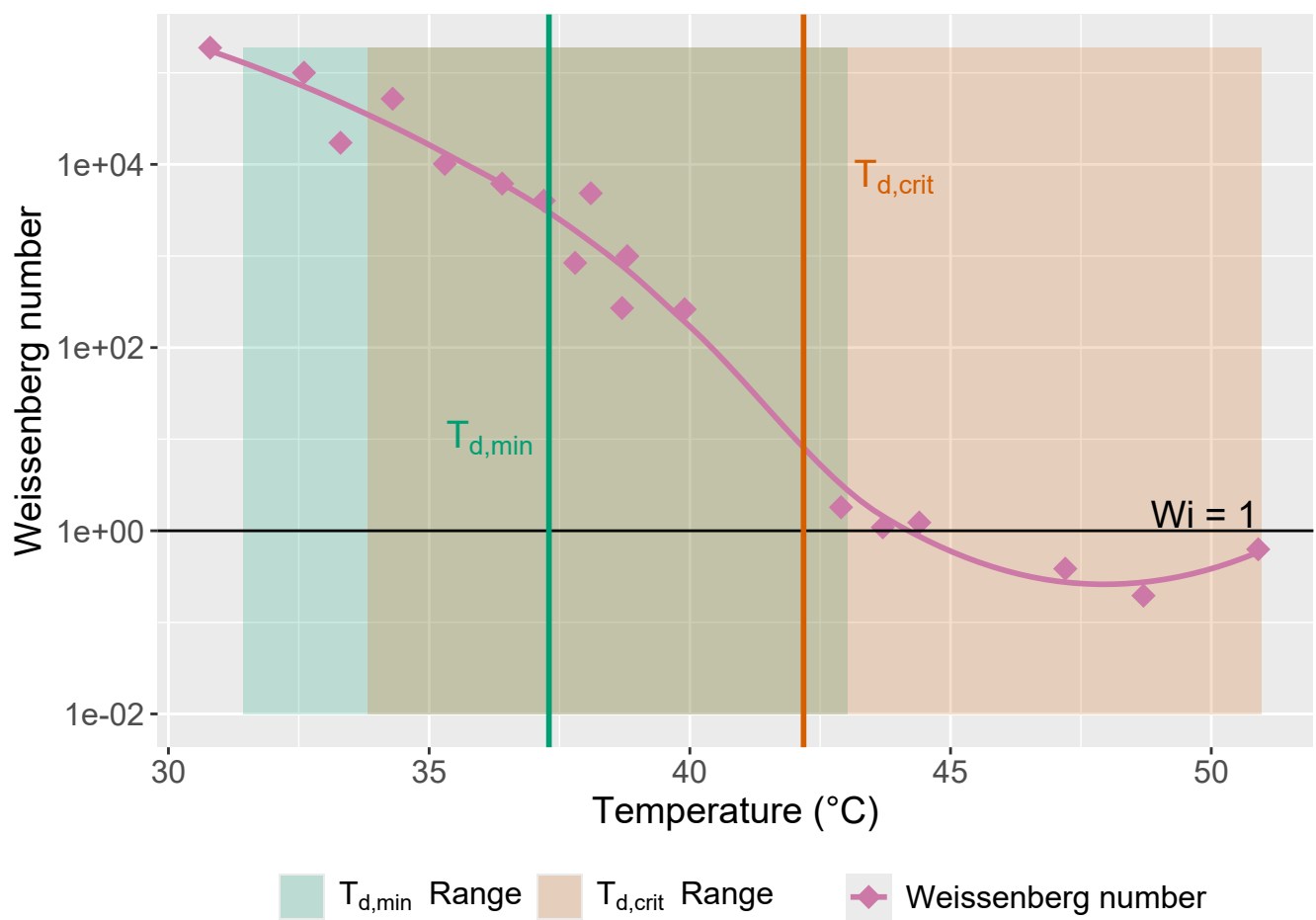

Supplement: Supplementary file 1 — Supplementary Information. [file 41598_2026_50635_MOESM1_ESM.zip › fig_Rheology_Temperature_Plot_at_380_3_LVE_60_Wi.pdf]

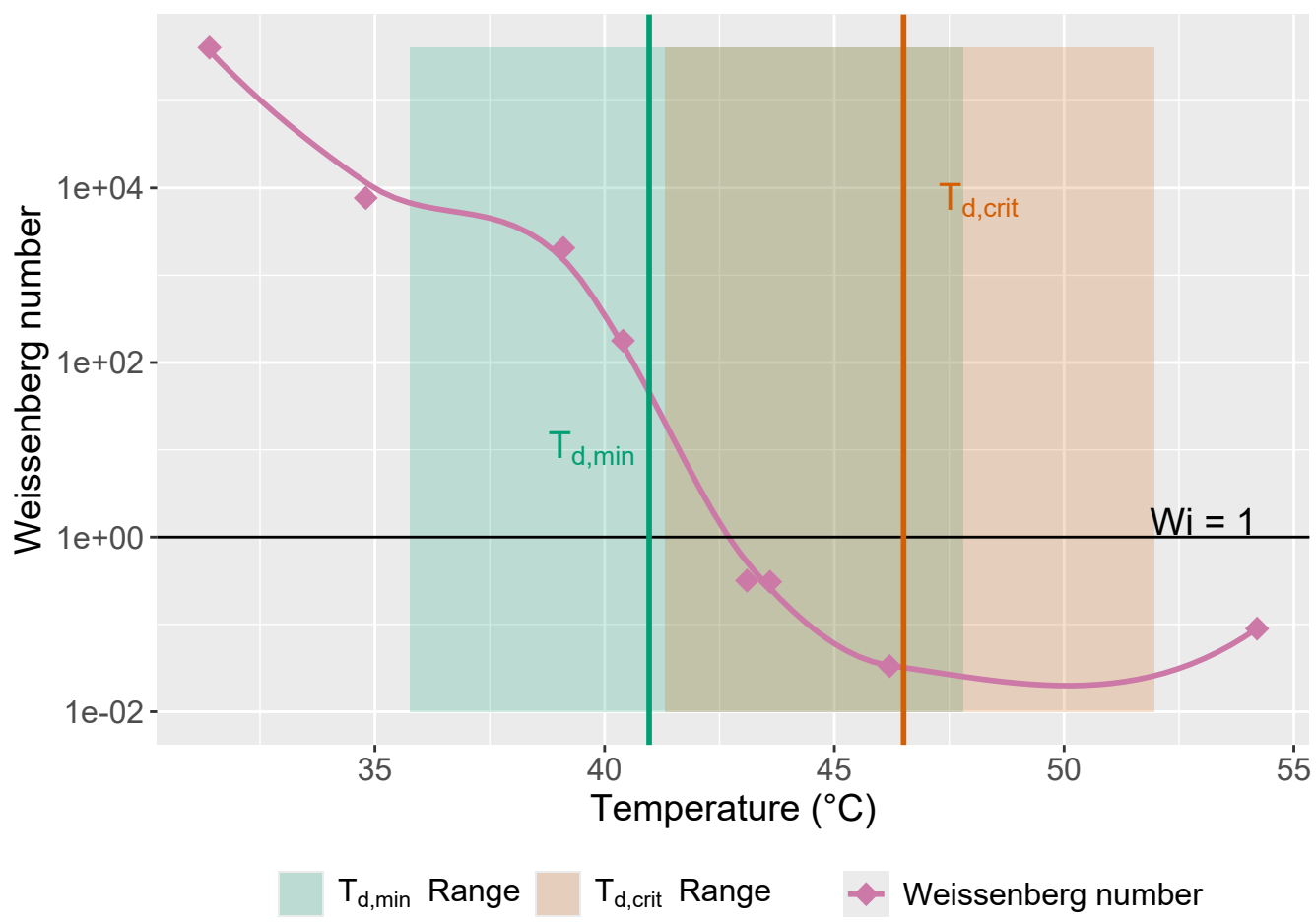

Supplement: Supplementary file 1 — Supplementary Information. [file 41598_2026_50635_MOESM1_ESM.zip › fig_Rheology_Temperature_Plot_at_380_9_LVE_60_Wi.pdf]

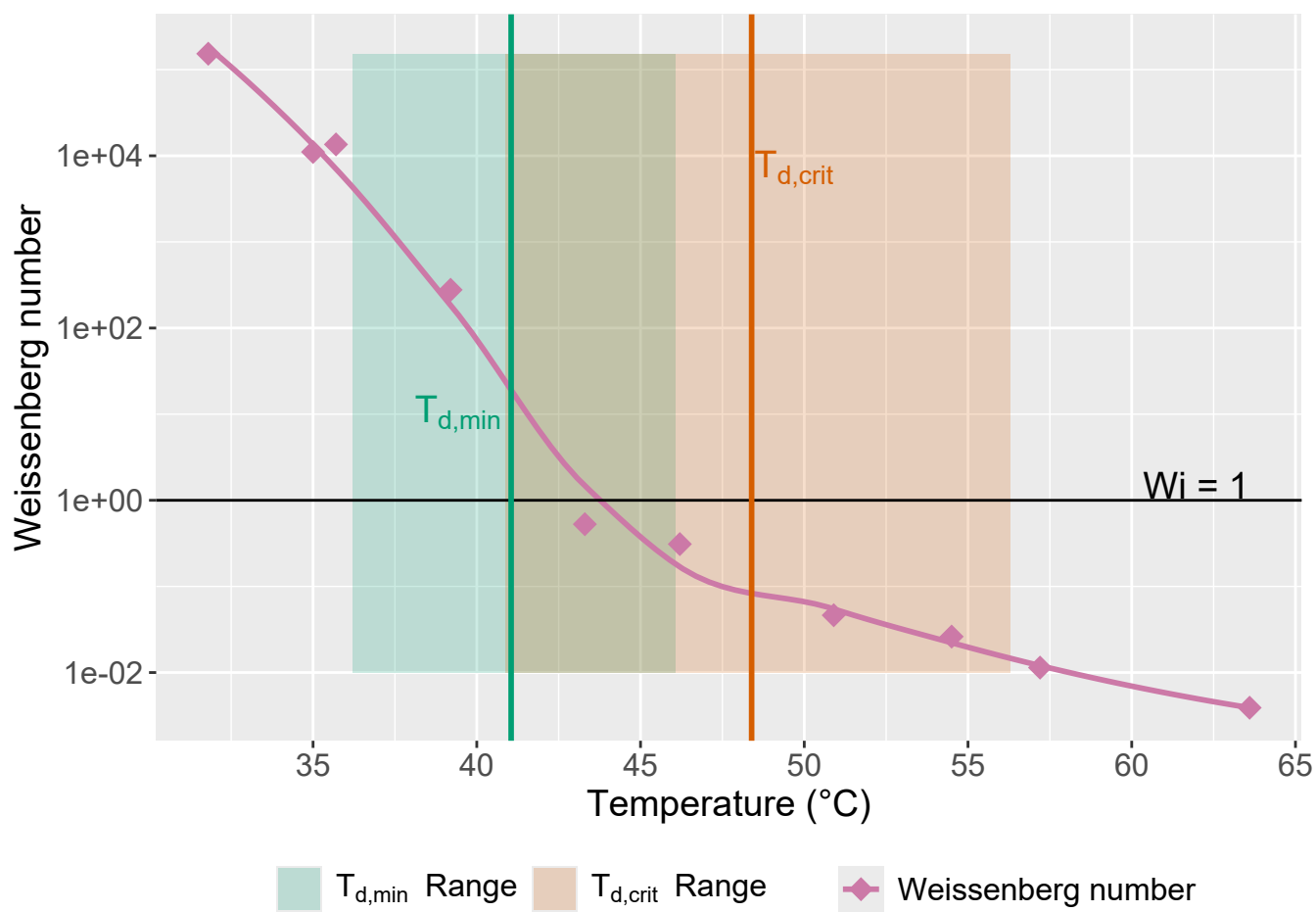

Supplement: Supplementary file 1 — Supplementary Information. [file 41598_2026_50635_MOESM1_ESM.zip › fig_Rheology_Temperature_Plot_at_380_12_LVE_60_Wi.pdf]

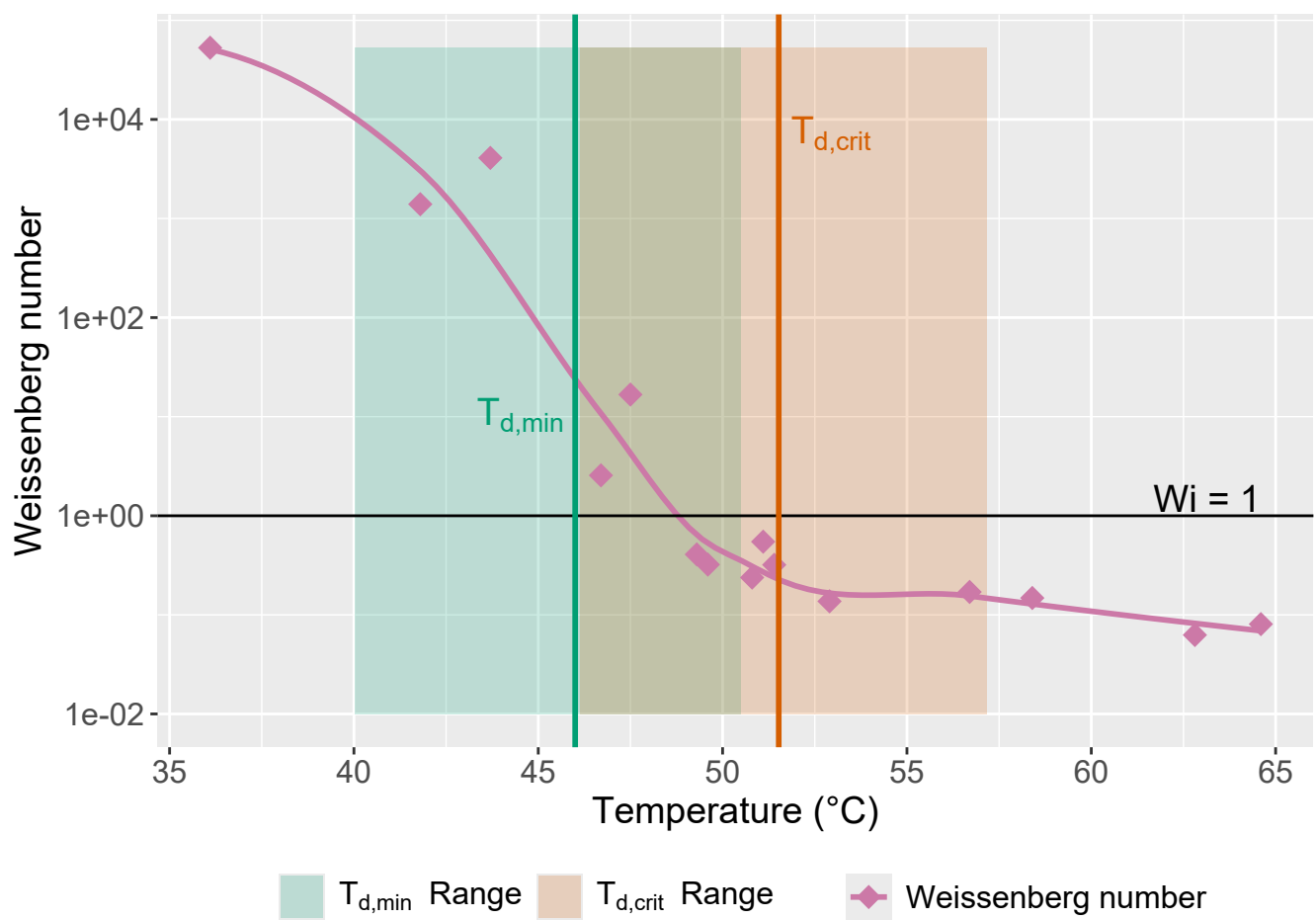

Supplement: Supplementary file 1 — Supplementary Information. [file 41598_2026_50635_MOESM1_ESM.zip › fig_Rheology_Temperature_Plot_at_380_15_LVE_60_Wi.pdf]

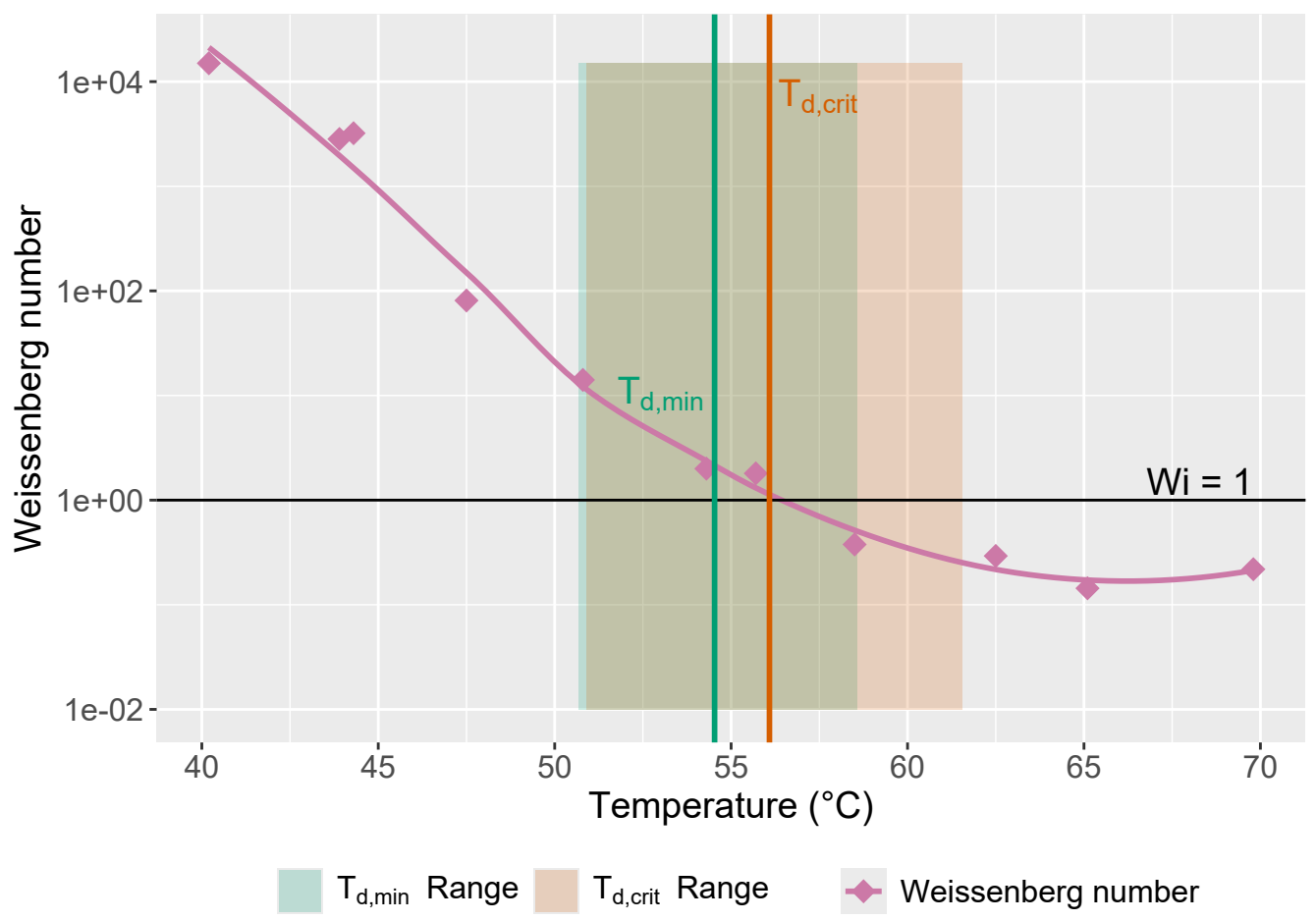

Supplement: Supplementary file 1 — Supplementary Information. [file 41598_2026_50635_MOESM1_ESM.zip › fig_Rheology_Temperature_Plot_at_380_18_LVE_60_Wi.pdf]
